# Correction: “We are pleading for the government to do more”: Road user perspectives on the magnitude, contributing factors, and potential solutions to road traffic injuries and deaths in Ghana

**DOI:** 10.1371/journal.pone.0315233

**Published:** 2024-12-04

**Authors:** Aldina Mesic, Barclay Stewart, Irene Opoku, Bradley H. Wagenaar, Bilal Andoh Mohammed, Sulemana Abdul Matinue, Manal Jmaileh, James Damsere-Derry, Adam Gyedu, Charles Mock, Angela Kitali, Daniel Hardy Wuaku, Martin Owusu Afram, Caryl Feldacker

The funding statement for this paper is incorrect. The correct funding statement is as follows: This work was supported by the Fogarty International Center of the National Institutes of Health under grants D43TW009345 (awarded to the Northern Pacific Global Health Fellows Program), D43TW007267, and R35-TW009345.

## References

[1] Mesic A, Stewart B, Opoku I, Wagenaar BH, Andoh Mohammed B, Abdul Matinue S, et al. (2024) “We are pleading for the government to do more”: Road user perspectives on the magnitude, contributing factors, and potential solutions to road traffic injuries and deaths in Ghana. PLoS ONE 19(5): e0300458. 10.1371/journal.pone.0300458 38787863 PMC11125548

