# Supplementary material for: “We are pleading for the government to do more”: Road user perspectives on the magnitude, contributing factors, and potential solutions to road traffic injuries and deaths in Ghana
Source: PLoS One. 2024 May 24;19(5):e0300458. doi: 10.1371/journal.pone.0300458 (PMC11125548; doi:10.1371/journal.pone.0300458)
Supplement: S1 File — (DOCX) [file pone.0300458.s001.docx]

**Supporting Information A.** Road user semi-structured interview guide.

| Introduction script    Good morning/afternoon. Can I have a few minutes of your time?  ● My name is X, and I am from the Kwame Nkrumah University of Science and Technology (TECH). We have been doing research with a university in the US on the causes and possible ways to reduce accidents in Ghana.  ● We will pay you 50 cedis for your time.  ● We want to know your thoughts, opinions, and experiences about the causes of accidents and deaths so we can plan projects with the government.  ● Your participation in this interview is voluntary, and you do not need to answer any questions that you do not want to.  ● Please do not be shy. It won't take long – about 20 minutes.  ● I would like to record our discussion so that we do not miss anything you say. Our discussion is completely confidential, we will not include your name with the recording, and we will not share your views with anyone outside of the team.  o Is it okay to record our conversation?  ● Now, we will go through a consent form together for you to sign.  ● Can you please tell me your:  o Age  o Occupation  ● Do you have any questions before we start? |
| --- |

| Screening question  ● How do you usually get around? How do you get to work? Do you use this road often?  ● For example  o walking, public transport (trotros), motorcycles, cars, taxis, trucks, riding a bike, tricycles (i.e., pragya) |
| --- |

| General and pre-event phase questions  ● How would you describe this area to others? Is this road busy?  ● How big of a problem do you think accidents are here?  o What do you think causes accidents here?  ▪ Road conditions (such as potholes, lack of sidewalks), abandoned/broken down vehicles, overspeeding, wrong overtaking, traffic  o What do you think decreases the risk of an accident?  o Are there some people who are more likely to get into an accident (for example, children, hawkers)?  o Which age of children?  ● Sometimes, personal stories can make road traffic problems more real. However, we know this can be sensitive.  ● If you feel comfortable, can you share a story from an accident with me? Your own or someone else you know?  ● Can you tell me of a story about a child getting in an accident on the roads, if you have one?  ● Now, let’s talk now about the police and their role.  ● What do you think about the police’s enforcement of laws now?  o For example, speed, motorcycle helmets, unlicensed driving, broken vehicles  o Do you think this affects collisions?  ● If you had the power, what would you do to change the situation here? |
| --- |

| Event questions  Once an accident does happen,  ● What do you think causes people to die or get hurt, compared to just getting into a collision without getting hurt?  o For example, what about the condition of the vehicle or trotro makes it more likely for a severe injury or death?  ▪ Like seat belts not working in cars/trotros, cars being old and not having airbags, position of seats, crowding  o Generally, which people typically get injured or die in an accident?  ▪ For example, pedestrians, children, motorcyclists, bicyclists, and hawkers  ▪ Those without a helmet, those who do not use seat belts  o What about the environment (such as the roads) makes it more likely for a severe injury or death?  ▪ For example, abandoned/broken down vehicles on the road, lack of sidewalks, potholes, traffic volume on roads  ● What can be done to reduce the number of severe injuries and deaths here? |
| --- |

| Post-event questions  ● When people get into an accident or get hurt, what happens?  o For example, do people call the police? Do people come help? Does an ambulance come? Tell me about what happened.  ● When you call an ambulance, do they come?  o How long would an ambulance take to arrive?  ● Who gets an ambulance, and who doesn't?  o For example, does it depend on if you are in an urban or rural area? Or the conditions of the road? Or if it's a major road and it causes congestion?  ● If you had the power, what would you do to improve care after an accident?  o For example, increasing number of ambulances, training people around in first aid |
| --- |

| High-level questions – Intervention characteristics  ● In your opinion, how much of a problem are accidents in Ghana?  ● Does the government consider your views when they make decisions on road safety?  ● What is the government currently doing to reduce accidents?  ▪ For example, speed bumps, enforcement by police, pedestrian bridges, education campaigns  ▪ Have you heard of those?  ▪ Have you seen those?  ● Why do you think the government chooses these?  ● Are they considered better?  ● Are they cheaper? Do you think the government considers cost when they pick what to do?  ● Where do ideas about road safety come from?  o Do you think the government looks to other countries?  o Or at research?  ● We know other countries use enforcement cameras, where people get a fine immediately if they speed or run a red light – do you think we can do such a thing in Ghana?  ● Why?  ● What mark will you give the government on a scale of 1-10, with 10 being the best? Why that mark? |
| --- |
| Closing question    Finally, our last question for you is:  ● If you had the power, what would you do to reduce accidents, injuries, and deaths on the roads nationally?  o What would you do for pedestrians?  o What about motorcyclists?  o What about children?  ● Is there anything else about collisions, injuries, or deaths on the roads that we haven’t discussed today that you would like to tell me?  ● Thank you for your time and participation in this important work. |

**Supporting Information B. Consolidated criteria for reporting qualitative studies (COREQ): 32-item checklist**

Adapted from:

Tong A, Sainsbury P, Craig J. Consolidated criteria for reporting qualitative research (COREQ): a 32-item checklist for interviews and focus groups. International journal for quality in health care. 2007 Dec 1;19(6):349-57.

| Item and guiding questions  Guide questions | | Response |
| --- | --- | --- |
| Personal characteristics | | |
| 1. Interviewer/facilitator   Which author/s conducted the interview or focus group? | | BAM and SAM conducted the interview. IO was the field supervisor. |
| 1. Credentials   What were the researcher’s credentials? E.g. PhD, MD | | The lead researcher has a MPH and was completing this work as part of her PhD. |
| 1. Occupation   What was their occupation at the time of the study? | | The lead researcher was a fellow in a PhD program. One data collector was a MPH student, whereas another worked as clinical research staff at a Ghanaian hospital. |
| 1. Gender   Was the researcher male or female? | | The researcher was female, while the interviewers were both male. |
| 1. Experience and training   What experience or training did the researcher have? | | Both interviewers were trained in qualitative methods and had prior experience collecting and analyzing data. The lead researcher has years of experience working on mixed-methods studies in sub-Saharan Africa. |
| Relationship with participants | | |
| 1. Relationship established   Was a relationship established prior to study commencement? | | No, this was not part of the study. |
| 1. Participant knowledge of the interviewer   What did the participants know about the researcher? e.g. personal goals, reasons for doing the research | | We informed participants that the Kwame Nkrumah University of Science and Technology was collaborating with a university in the United States on ways to reduce road traffic crashes in Ghana. |
| 1. Interviewer characteristics   What characteristics were reported about the interviewer/facilitator? e.g. Bias, assumptions,  reasons and interests in the research topic | | No other information was provided. |
| 1. Methodological orientation and theory   What methodological orientation was stated to underpin the study? e.g. grounded theory,  discourse analysis, ethnography, phenomenology, content analysis | | We used an interpretative perspective to guide tis work. We use a mixed deductive-inductive approach. For the deductive, we used Haddon’s Framework and the Consolidated Framework for Implementation Research to guide content analysis. We used interpretative phenomenological analysis for the inductive approach. |
| Participant selection | | |
| 1. Sampling   How were participants selected? e.g. purposive, convenience, consecutive, snowball | | We purposively selected road users in 100 meter locations determined to be statistically significant clusters of injury severity on national roads. At these locations, we used a prior maximum variation sample to purposively select vulnerable and non-vulnerable road users of various types. |
| 1. Method of approach   How were participants approached? e.g. face-to-face, telephone, mail, email | | Participants were approached face-to-face and told about the objective of the study. |
| 1. Sample size   How many participants were in the study? | | A total of 24 participants were involved. |
| 1. Non-participation   How many people refused to participate or dropped out? Reasons? | | We did not have any participants refuse to participate. Given this study comprised of only one interview, drop out was not relevant. |
| Setting |  |  |
| 1. Setting of data collection   Where was the data collected? e.g. home, clinic, workplace | | The data was collected roadside. |
| 1. Presence of non-participants   Was anyone else present besides the participants and researchers? | | All of the locations were busy areas, either with pedestrians, cyclists, motorcyclists, and cars in urban areas, or with vehicles in the rural locations. Often, others were in the same area but the participants were taken aside to a location where the intervention could be conducted privately. |
| 1. Description of sample   What are the important characteristics of the sample? e.g. demographic data, date | | We conducted the study in November and December 2022. We have included the important characteristics including location, sex, age, road user type, and vehicle type in Table 1. |
| Data collection | | |
| 1. Interview guide   Were questions, prompts, guides provided by the authors? Was it pilot tested? | | We developed and used a semi-structured interview guide as part of this study. This is included in *the Supplementary Materials.* Four research staff members tested the guide internally and externally in four interviews in a busy location in Accra. |
| 1. Repeat interviews   Were repeat interviews carried out? If yes, how many? | | No repeat interviews were carried out. |
| 1. Audio/visual recording   Did the research use audio or visual recording to collect the data? | | We audio recorded each interview for transcription, translation, and quality checking. |
| 1. Field notes   Were field notes made during and/or after the interview or focus group? | | One interviewer took field notes before, during, and after the interview. |
| 1. Duration   What was the duration of the interviews or focus group? | | The interviews lasted between 13 to 60 minutes, with a mean of 25 minutes. |
| 1. Data saturation   Was data saturation discussed? | | Data saturation was discussed and a key part of deciding how many individuals should be interviewed as part of the study. |
| 1. Transcripts returned   Were transcripts returned to participants for comment and/or correction? | | This was not part of the research study. Participants were interviewed a single time roadside without any follow up. |
| Data Analysis | | |
| 1. Number of data coders   How many data coders coded the data? | | Two staff members were involved with coding the data and checking the codes. Other staff members reviewed the codes and respective quotes. |
| 1. Description of the coding tree   Did authors provide a description of the coding tree? | | We did not provide a description of the coding tree, but can upon request. The coding tree included themes that fit within from Haddon’s Framework and the Consolidated Framework for Implementation Research, and emerging themes from the inductive analysis. |
| 1. Derivation of themes   Were themes identified in advance or derived from the data? | | Themes were identified both in advanced (the deductive analysis) and from the data (the inductive analysis). Some high level codes, such as ‘human-level factors’ came directly from Haddon’s Framework, whereas others such as the specific factor, or emerging themes such as the role of economic status, were derived from the transcripts. |
| 1. Software   What software, if applicable, was used to manage the data? | | We used NVivo to manage the data. |
| 1. Participant checking   Did participants provide feedback on the findings? | | This was not part of our study. |
| Reporting | | |
| 1. Quotations presented   Were participant quotations presented to illustrate the themes / findings? Was each  quotation identified? e.g. participant number? | | We did present numerous participant quotations along with identifiers (e.g., transcript number). |
| 1. Data and findings consistent   Was there consistency between the data presented and the findings? | | We present findings and supporting data consistently. |
| 1. Clarity of major themes   Were major themes clearly presented in the findings? | | We present the major themes in clear sections. The major themes are that 1) road traffic collisions, injuries, and deaths are a major issue; 2) there are numerous contributing factors, many of which related to socioeconomic conditions; 3) there was moderate awareness of existing road safety measures but calls for more to be implemented and for existing ones to be adapted; 4) road users largely felt considered or involved in road safety decision-making. |
| 1. Clarity of minor themes   Is there a description of diverse cases or discussion of minor themes? | | Minor themes are described as part of the major theme subcategories. Details on contributing factors and proposed solutions (both minor theme categories) are presented in Table 2 with a description and illustrative quote. |
