# Supplementary material for: “We are pleading for the government to do more”: Road user perspectives on the magnitude, contributing factors, and potential solutions to road traffic injuries and deaths in Ghana
Source: PLoS One. 2024 May 24;19(5):e0300458. doi: 10.1371/journal.pone.0300458 (PMC11125548; doi:10.1371/journal.pone.0300458)
Supplement: S2 File — (ZIP) [file pone.0300458.s002.zip › Transcripts to share/Participant _121_vulnerable.docx]

**Participant Number: 121**

**Language: Dagbani**

**Type of hot spot: Rural**

**Sex: Male**

**Road user type: Motorcyclist**

Interviewer: my brother what work do you do, and do you use this road often?

- Participant: to be frank all the time I ride motor using this road, I am a butcher doing a motorcyclist business and all the time is my road,

Interviewer: So, all the time you are in this road?

- Participant: Anytime I am in this road,

Interviewer: Which places do you go?

- Participant: I do often transport meat from the butcher house to the next town, and sometimes I go as far as Tamale. I use road even up to Damango junction and Yapei, and all the time I sell meat

Interviewer: So, looking at this road is it busy?

- Participant: Yes all the time this road is busy, this road is always busy

Interviewer: So, if you check are accidents here rampant?

- Participant: Yes, the fall of vehicles here is accountable, motor people falls down, vehicles have been falling down and sometimes. A lot of vehicles here also loses their way and runs to the bush here often causing accidents.

Interviewer: So, what will make accidents here decrease or risk of accidents reduce?

- Participant: truly, this place to me I think the overtaking here are the causes of the accidents here, and there are a lot of sharp corners here, so if those thing like speeding, speeding if we reduce it would have prevented some of them.

Interviewer: Which people are mostly affected by accidents here, is children, passengers, hawkers?

- Participant: truly this place the people that are often knocked down by vehicles are the Fulani people, motor riders riding with speed, and overtaking cars. A car can be doing over taking and when you move on top of road from your house and it will cause an accident, that is what I have seen.

Interviewer: Have ever seen a child knocked down by a vehicle here?

- Participant: To be frank, in this road I haven’t seen a vehicle down a child, yes a vehicle hasn’t knock down a child here.

Interviewer: Have you ever seen someone knocked down by a vehicle here, or someone told you how an accident happened here that you would like to share with us?

- Participant: Yeah! True I have seen a vehicle that fell here, but I haven’t a vehicle that knocked down a child in this road. But I have seen a vehicle that fell here, and with the vehicle falling, the problem was the vehicle climbed the mini hill and was coming and the tire of the vehicle burst, it was a sprinter bus and the front tire burst, and I was coming from a junction, so when the front tire burst and I was coming riding motor from the junction, so at the time the front tire burst, I move out of the road in to the bush and the bus fell at the other side of the road, what I saw was a woman and her face side was bloodied, so I reported to police, the police were at Ntereso, so the police came sharp and pick some of the victims to the hospital Yapei.

Interviewer: Now let talk about the role of the police and enforcement of laws in the road, how do you see their work?

- Participant: To be frank, with regards to accidents, the police are not all that convincing in their work, there are not doing well, because accident can happen and you call them and all their mind in money, because what I am saying is if the police is sitting at the barrier and you come and inform him, they wont mind you, they will do as if he hasn’t seen you, sometimes until the situation becomes worst before they come and by that time maybe the victim may die or the condition becoming worst, the victim situation will reach what it wasn’t supposed to.

Interviewer: So do the police check over speeding, unlicensed driving, wearing of helmet and seat belts?

- Participant: They don’t check,

Interviewer: So they not checking does this affect number of accidents?

- Participant: Yes, it increases the number of accidents, because sometimes some accident and it impact can be reduce when you are wearing a helmet, but some accidents resulting to deaths is because of the absence of the helmet

Interviewer: If they give you power and you are capable what would you do in this road so that accidents will reduce?

- Participant: truly If I was given the power, and strength my problem would have been the sharp corners, and the overtaking, I would have done things for people to run from doing over taking here.

Interviewer: So, let me ask, you made mention of what do you think makes people get more injured whenever there is an accident, is it the how the seats are positioned, absences of seat belts, or what always make it affect a lot of people?

- Participant: Truth be told, anytime there is an accident especially the sprinter bus that got involved in an accident that I witnessed, the problem was two, first inside the sprinter there were no seat belts that is one and the second one the top speed at which the bus was going, it wasn’t supposed to go like that, that is the problem that are causing the accidents. Inside the bus, the seats were overcrowded so if the bus should involve in an accident those thing be dangerous for passengers.

Interviewer: So anytime a vehicle had an accident which people are mostly affected, is it children, motor rider, hawkers, which people are affected most?

- Participant: Truly, if a vehicle is involved in an accident, it affects those people inside the vehicle, to this our side, if a vehicle get involve in an accident those people inside the bus is affected most, especially drivers is drivers that vehicles kills. Is drivers that vehicles kills.

Interviewer: How about the road environment, which will make how the road is made also contribute to an accident, is it potholes or what?

- Participant: Truly our roads doesn’t have problem especially our Ntereso area, the road doent have a problem but the problem is it has a lot of corners, and the sharp corners is what is causing a lot of accidents.

Interviewer: So, what can we do so that accidents and deaths will reduce here in your opinion?

- Participant: In my opinion, If we are all patience, especially the drivers, motorist or bicycle riders if you are on the road and you ride small small, if you curve the corner and you go slowly and you will definitely reach.

Interviewer: So, looking the accident who made mention of, anytime there is an accident here what do you do, do you call the police, do you call ambulance, tell me what you do?

- Participant: Yay, they call the ambulance, is the ambulance that they call, but in case the ambulance is far, then we call police to come and pick the victims to the hospital.

Interviewer: So anytime they call the ambulance do they come, and how long do they take before they come?

- Participant: To be frankly, at the time the accident occurred, we just got a new ambulance but we haven’t seen the ambulance fault, anytime we call them they come fast,

Interviewer: Where is the ambulance?

- Participant: The ambulance is in Yapei

Interviewer: So, if you call the ambulance and there are coming to pick the victims, do they look at the caliber of person involved or they just come?

- Participant: Truly as for the ambulance, the moment you call them and tell them that an accident occurred here, they are listening to that, they don’t care about the ethnic background of victims is patient or victims they just coming, when they hear accident, they just come and pick the victims to the hospital

Interviewer: You made mentioned that you have one ambulance in Yapei right? So if an accident happen and the victims are many, and now we have one ambulance how can we handle it, in your opinion what can we do to send the victims to the hospital?

- Participant: Truly we are pleading, we have only one ambulance from Yapei-Kusauga Constituency it is only one, and Bupei has two, so be frank this our area before an accident occurs and Buipe own arrives it will delay. But Yapei is closer and Yapei has only one, so in case there three or four people that got injured and ambulance arrived it will take a lot of time, but truly sometimes we even use our own buses, we use some alhajis bus sometimes in addition to the ambulance to carry victims to the hospital,

Interviewer: So, if you have power you will increase the number of ambulances right?

- Participant: Truly if I have the power, I will increase the number of ambulances

Interviewer: Looking at Ghana, in your opinion are accidents and deaths in our road a problem?

- Participant: It is a big problem for Ghana,

Interviewer: Inaudible… why do you say so?

- Participant: why am I saying is a problem for Ghana, if you look at Ghana, we are not many and we will be dying again like this so that is a problem, We those living human being we are making country to be alive so if we all die then there will be no country that is why I said is a problem for Ghana

Interviewer: So, looking at government do they come to listen to your concerns, do they come to ask what do your need regarding road safety, or they just come and do whatever they want and go?

- Participant: Truly government doesn’t come and ask about the thing we want, they will come and do whatever they feel like doing and do and then go, even the time that we wanted speed rump we did not get before we got it we threaten them that we are going to divide the road in to two to the extend they brought police and military to come and catch that we don’t have the right and we told them the reason why we are doing that before they came and did the speed rump for us.

Interviewer: So, what and what do you see government do here and other places to reduce the accidents, do they do speed rumps, what is government doing?

- Participant: Truly here government is not doing anything to prevent road accidents, he is not doing anything

Interviewer: The speed rump you said government did for you people, if government is doing speed rumps and other things do they look at the amount of money used or they just work, maybe sometimes some of these speed rumps are costly, or so in your opinion do they look at the money involve?

- Participant: {Inaudible}.. I would not be able to talk about those things but the speed rump I told you about that we wanted to divide the road into two before they came and did, that speed rump one MP did but he is no more that is the one in Ntereso, that MP is no more the name is Abu Kamara did the speed rump but he is no more, he said people should be patient, they told us to close the trench we dug in road and he said no problem and he made constructors came and do the speed rump, it after he did the speed rump and was going home and had an accident and died

Interviewer: So, let me ask you all the road safety things government is doing, where do government get their ideas from, do they go to a certain country and see what they are doing and they come back and do or they do their own research?

- Participant: In my thinking the government just look at what its counterparts government they are doing, and they also do and just do, because how it is supposed to be done is not done that way. They are doing let me also do that is what government does.

Interviewer: Looking at some countries they have speed cameras mounted on roadsides and they monitor those cameras, if any car runs over speed and that person is caught and person next time he wont do that if we bring such cameras to Ghana will it work?

- Participant: Yes, it will work,

Interviewer: Why do you say so?

- Participant: if they were doing something like that our accidents would have reduce, because looking at drivers they are stubborn especially some of them they take drugs, so if he take the drug he doesn’t know what is happening in his front and he carrying human lives. The government should also know that we the people/passengers who voted for you and you are at where you are if they do that it would have been nice to me, it would have been nice to me personally.

Interviewer: Looking at all the works government is doing, if they say you should mark government from 1 to 10 where will you give him, one means he has not done anything, 10 means he has done perfectly, which number would you give?

- Participant: Truly I will mark him 5

Interviewer: Why do you say five (5)

- Participant: first of all he has made us road, secondly he has done his best from ambulance for safety accident, thirdly he has been looking after Ghana but four is the reason why I am marking him four or five is how he has pick people around who talk about road safety issues {inaudible}.. that is why I have given him five.

Interviewer: This is all our questions, if they give you the power to work and reduce accidents in Ghana, what will you do pedestrians and those who crosses the road, motor riders, children going to school and uses the road, what will you do for them children, motor riders so that when those are done accidents will reduce?

- Participant: To be frank firstly school children, before you cross the road to school when we were in school our masters taught us before you cross the road you will look three time before you cross, that you will look at your red oh! right, your look second oh! left and then your right third before you cross. But motor accidents if we reduce our speed or vehicle, vehicle every vehicle and vehicles every vehicle is supposed to have seat belts and enough space {inaudible}… so that you can seat free. Motor people should be using helmets all the time otherwise in case of accident you can die and top speed we should avoid top speed that would have been better.

Interviewer: Thank you so much do you have anything to add to these conversations?

- Participant: Yes truly what I have to add is if the government would have look at the accidents area especially Nteresp area or Yapei-Kusaugu Constituency area, truly, just recently, yesterday motor accident killed one woman {inaudible}.. they just buried the person and I am going to Damango junction and it was accident and top speed, the woman close from the farm and wanted to cross and knocked down by a bicycle, so we are pleading with government they should try and look at how they can help us anything that can help us with regards to accidents we are pleading we would have been grateful.

Interviewer: Thank you so much.
