# Supplementary material for: “We are pleading for the government to do more”: Road user perspectives on the magnitude, contributing factors, and potential solutions to road traffic injuries and deaths in Ghana
Source: PLoS One. 2024 May 24;19(5):e0300458. doi: 10.1371/journal.pone.0300458 (PMC11125548; doi:10.1371/journal.pone.0300458)
Supplement: S2 File — (ZIP) [file pone.0300458.s002.zip › Transcripts to share/Participant_101_non_vulnerable.docx]

**Participant Number: 101**

**Language: Twi**

**Type of hot spot: Urban**

**Sex: Male**

**Road user type: Pedestrian and tro-tro user**

Interviewer: How do you get to work?

- Participant: Please I come with car.

Interviewer: Is it a car taxi uber or public transport(trotro)?

- Participant: Please it’s public transport.

Interviewer: Over the past ten years, this area has recorded one hundred and two accidents, out of that figure Twenty-Eight people have died. So, this is why we are here to find out the causes of these accidents. Can you describe the road or accidents in this area to us, how scary?

- Participant: Okay! What I can say about this is that vehicles travelling on this road should slow down. Although there is a bus stop here but some drivers will stop in the middle of the road to alight passengers. Instead of the drivers to entering the bus stop well to alight passengers, they will not. They prefer to stop on the road to alight passengers. There are also some people who, when crossing the road, do not look in any direction and will simply cross without taking caution. This can cause a car to mount the pavement, cross into another lane, and cause a crash. The government may need to deploy police officers to enforce pedestrian use of footbridges and to ensure drivers do not stop on the road but instead enter the bus stop properly. That is what I have to say.

Interviewer: What do you think decreases the risk of an accident?

- Participant: When cars are approaching, drivers should enter the bus stop and park appropriately to avoid causing accidents. Similarly, pedestrians should use the footbridge instead of crossing the road, as this can also lead to accidents. That's all I have to say.

Interviewer: Are there some people who are more likely to get into an accident (for example: children, hawkers)?

- Participant: Yeah! Sometimes individuals who are intoxicated with alcohol may cross the road and be hit by a moving vehicle.

Interviewer: Does it invovle Adults or children?

- Participant: Sometimes adult drunkard. Sometimes too, a small child who is supposed to have climb up there [footbridge] but will not and rather cross the road here. This can cause accident.

Interviewer: How old are the children?

- Participant: Okay about seventeen to eighteen. [then not a child but an adult]

Interviewer: I want to know the ages of the children you were talking about.

- Participant: Some of them may be about ten years or fifteen years.

Interviewer: Sometimes personal stories can make road traffic problems more real. However, we know this can be sensitive. Have you ever witnessed any road accident here before?

- Participant: Yeah, I ‘ve seen about two or three.

Interviewer: Then share with us?

- Participant: Okay The first one was about A cargo truck carrying plantain from Kumasi to Accra and was traveling at high speed. When it arrived here, the driver's brakes failed, causing the truck to move from the inner lane to the outer lane and eventually crash into a parked car on the other side of the road. The truck then settled on the sunshade at the bus stop, resulting in significant damage to the vehicle's metal structure. A mobile money vendor who was nearby was lucky to escape death thanks to the intervention of God. The second incident also involves a bus parked by the side of the road one evening. A motorcycle was approaching from behind when a pedestrian began crossing the road without realizing the motorcycle was coming. Unfortunately, the motorcycle knocked down the pedestrian, causing severe injury to the pedestrian's eyebrows. The third one was a drunkard who was crossing the road without care and a car knock him down.

Interviewer: Can you tell me of a story about a child getting in an accident on the roads, if you have one? Which of the three incidents you’ve narrated previously involves a child?

- Participant: That was a different instance, and with that, the child was knocked down by a vehicle.

Interviewer: How old was the boy?

- Participant: Thirteen

Interviewer: Now, let’s talk about the police and their role. What do you think about the police’s enforcement of laws now? For example, over speed, motorcycle helmets, unlicensed driving, broken vehicles. Do you think this affects crashes?

- Participant: Okay what will be better is that, the bus stops over here which has been closed for almost a year now, the police have to remove it from there. The police should enforce the law on pedestrians to use the footbridge. And the police don’t have to allow anyone to cross the road. Also, the police should enforce the law on the drivers to prevent them from parking on the road or dropping off and picking up passengers. They should enforce the law to ensure that drivers enter the bus stop properly.They don’t have to allow truck to park at the bus stop. The police and the government have to do something about it.

Interviewer: Let me help you a bit. What about over speeding?

- Participant: Over speeding too is among the cause of accident here. Last time some car veered off the road and then crashed on the sunshade at the bus stop. Just have look at the bus stop you will see that its metals have crumple. This happened here as a result of over speeding. He too said he failed brake.

Interviewer: By what means can the police help prevent road accident. Let me help you. Example over speeding riding without helmet, driving without license broken down vehicle. What can the police do about it.

- Participant: The police should check and arrest any moto rider without helmet. Defaulters should be stopped and detained and then be worn that from now on if you are seen ridding without helmet again, I will arrest you to jail. Also, the police should enforce the law on drivers driving without license and then tell them to do the right thing. Also bicycle too the riders don’t have to ride on the high way because is unsafe for them. Over here is not like abroad where the drivers have sympathy a bit for others. A Ghanaian driver will not have sympathy, he will knock you to death. Even moto riders are being knock to death by cars, how much more bicycle. So, the police have to be checking those things.

Interviewer: If you had the power, what would you do to change the situation here?

- Participant: If I were the government, I will instruct policemen to arrest drivers who drive without license and moto riders who ride without helmet. Also, to check over speeding. If I even become the government I will construct separate road for bicycle, moto bikes and cars. And will make police to be checking things.

Interviewer: Once an accident does happen, what do you think causes people to die or get hurt, compared to just getting into a crash without getting hurt? For example, what about the condition of the vehicle or trotro makes it more likely for a severe injury or death? Like seat belts not working in cars/trotros, cars being old and not having air bags, position of seats, crowding

- Participant: Some vehicles overspeed, which can cause accidents. Additionally, some drivers do not carefully enter intersections, which can also result in accidents. Furthermore, some pedestrians are not careful when crossing the road and may cross lackadaisically, which can be hazardous.

Interviewer: For example, what is the appearance of the car?, no seatbelt. Do you understand? I’m given you a clue so that you will understand what I mean.

- Participant: Some of the drivers do not put on their seatbelts. Also Some of the vehicles are very weak so if an accident occurs it’s easier for passengers to get hurt. Some of the car’s brake is air so any little abstraction then there’s a brake failure which causes accident.

Interviewer: Generally, which people typically to get injured or die in an accident? For example, pedestrians, children, motorcyclists, bicyclists, hawkers, those without a helmet, those who do not use seat belts.

- Participant: Ok! Some of the accident when it happened all the people in the vehicle die even it extend to pedestrians who are walking by the road side, hawkers by road side or anybody at all at that moment.

Interviewer: Does the accident affect children?

- Participant: Oh! Adult, old men children mix, all inclusive

Interviewer: What about hawker?

- Participant: It sometimes affects them too. At times even when you are walking by, the accident can affect you. For you meet your untimely death.

Interviewer: Let’s come to the next question. You said that when accident happens, it affects those by the road side, hawkers and children and grownups. What are the ages of the children?

- Participant: Some of the accident affect hawkers who are breastfeeding mothers and their babies sitting by the road side. All this as a result of bread and butter.

Interviewer: What about the environment (such as the roads) makes it more likely for a severe injury or death? For example, abandoned/broken down vehicles on the road, lack of sidewalks, potholes, traffic volume on roads.

- Participant: Some of the accident happened as result of a driver trying to swerve or save a moto rider from a crash then eventually accident occurs. Some drivers will say they have failed brake.

Interviewer: Just bring your attention to this side of the road. Focus here. Is the road good or not good?

- Participant: Oh, over here the road is good but the back there is the side that is not good.

Interviewer: What causes accident to happened here?

- Participant: some says they have failed break and then it happens just like that. Sometimes you will be there and then it just veered towards this area. Sometimes too accident happen as result of a moto bike Infront of a car and the driver trying to save that motor rider and then eventually accident happens like that.

Interviewer: What can be done to reduce the number of severe injuries and deaths here?

- Participant: They have to filled all potholes, the driver too they have to slow down their speed. To the drivers, before they move their car, they have to check their brake and other necessary things on their vehicles before they move.

Interviewer: When people get into an accident, or get hurt, what happens? For example, do people call the police? Do people come help? Does an ambulance come? Tell me about what happens. When you call an ambulance, do they come?

- Participant: I haven’t seen any ambulance here before after accident.

Interviewer: Do you call then?

- Participant: Yes, we do. Even If you call them, they will not come.

Interviewer: What about the police?

- Participant: As for the police they will come but it will be after someone has died.

Interviewer: Do people come to help?

- Participant: When it happened like that, we those who are around will pick a taxi and help convey the casualties to the nearby hospital may be before their people arrive.

Interviewer: If you had the power, what would you do to improve care after an accident? For example, increasing number of ambulances, training people around in first aid.

- Participant: We have to be careful. I will increase the number of ambulances so that when people call them, they will come fast to pick the casualties

Interviewer: Over the past ten years, our country has recorded seventy-eight thousand casualties and fourteen thousand deaths in an accident.For this reason, there is the need for us find out the cause of these death. In your opinion, how much of a problem are accidents in Ghana?What is the government currently doing to reduce accidents?

- Participant: Okay what the government should do is. I’ve said already, he has to deploy more police men on the road and also check over speeding drivers and pedestrians careless crossing of the road should be stopped. Some trucks over speed too much here, even their way of blowing horns will make you be confused to accident.

Interviewer: Does the government consider your views when they make decisions on road safety?

- Participant: Ok I don’t know whether he listen to us or not.

Interviewer: What is the government currently doing to reduce accidents? For example, speed bumps, enforcement by police, pedestrian bridges, education campaigns. Have you heard of those? Have you seen those?

- Participant: As for that no. I haven’t seen the government constructing any footbridge or pedestrian bridge etc. But he has to do all those things. He has to do speed rump. He has to do speed rump especially where school children normally cross the road.

Interviewer: What is the government currently doing to reduce accidents? or example, speed bumps, enforcement by police, pedestrian bridges, education campaigns. Have you heard of those?

- Participant: no I haven’t

Interviewer: Have you seen those?

- Participant: no I haven’t

Interviewer: So, you said you haven’t seen the government constructing foot bridge, speed rump, enforcement by police, education people on road safety etc. Why do you think the government chooses these? For example, speed bumps, enforcement by police, pedestrian bridges, education campaigns. Are they considered better? Are they cheaper? Do you think the government considers cost when they pick what to do?

- Participant: I don’t know.

Interviewer: Where do ideas about road safety come from? Do you think the government looks to other countries? Or at research?

- Participant: I think he got it from the research you are doing and he has been traveling too so I think he got the idea from there.

Interviewer: We know other countries use enforcement cameras, where people get a fine immediately if they speed or run a red light – do you think we can do such a thing in Ghana? Speed camera is type of camera build by the road side to check over speeding. In some countries these cameras are used there. do you think we can do such a thing in Ghana?

- Participant: Yes, it will help. Recently, I have seen the government constructing some camera poles at vantage point.

Interviewer: Why?

- Participant:: This is because if someone does something wrong, he can be captured and arrested Yes, it will really help. Even if any driver overspeed to hit and run, he’ll be seen and arrested.

Interviewer: What mark will you give the government on a scale of 1-10 with 10 being the best? Why that mark?

- Participant: I will give him one

Interviewer: Why?

- Participant: He hasn’t done enough to me on road safety.

Interviewer: Finally, our last question for you is: If you had the power, what would you do to reduce accidents, injuries, and deaths on the roads nationally? What would you do for pedestrians? What about motorcyclists? What about for children?

- Participant: You talked initially about speed cameras, so I think they should be installed on our roads. Also, drivers should slow down when driving. I will enforce the law so that pedestrians will use the footbridge. More speed bumps should be constructed. I will construct separate road for motor cyclist and bicycle users. I will advise pedestrians to be careful when walking on roadside

Interviewer: Is there anything else about crashes, injuries, or deaths on the roads that we haven’t discussed today that you would like to tell me?

- Participant: I would like to say that, drivers should be advised to check their vehicles to ensure that everything is intact, especially their brakes, before setting off. In the event that an accident occurs as a result of the driver's failure to do so, they should be arrested.

Interviewer: Thank you for your time and participation in this important work.
