# Supplementary material for: “We are pleading for the government to do more”: Road user perspectives on the magnitude, contributing factors, and potential solutions to road traffic injuries and deaths in Ghana
Source: PLoS One. 2024 May 24;19(5):e0300458. doi: 10.1371/journal.pone.0300458 (PMC11125548; doi:10.1371/journal.pone.0300458)
Supplement: S2 File — (ZIP) [file pone.0300458.s002.zip › Transcripts to share/Participant_102_non_vulnerable.docx]

**Participant Number: 102**

**Language: Twi**

**Type of hot spot: Urban**

**Sex: Male**

**Road user type: Mini bus rider / pedestrian**

Interviewer: How do you get to work?

- Participant My way of coming to work is, I stay at lapass.

Interviewer: So do you walk, public transport (trotros), motorcycles, cars, taxis, trucks, riding a bike, tricycles (i.e., pragya )

- Participant: Where I stayed, I don’t want to board a car so I pass a short way to here.

Interviewer: So, do you walk?

- Participant: Yes, I walk.

Interviewer: Do you use this road often?

- Participant: I use this road almost every day.

Interviewer: How would you describe this area to others? Is this road busy?

- Participant: Yes, very busy. Here and Achimota is very busy

Interviewer: How big of a problem do you think accidents are here?

- Participant: Accident about cars is not many but for the motor riders almost every day the riders knock pedestrians here.

Interviewer: Over the past ten years this area has recorded one ninety- two accidents, out of that figure twenty-eight people have died. So, this is why we are here to find out the cause of these accidents. What do you think causes accidents here?

- Participant Number one, the main reason for the construction of the foot bridge is for the pedestrians to use so that they will not cross the road. But due to our stubbornness and lazy we don’t use it. They prefer to cross the road instead using of the footbridge. So, it causes accident. Some of the pedestrians too when they are crossing the road, they will look at anywhere and then cross the road, eventually there is a knock down. But the good thing to have been done is to use the foot bridge so that there will not be any knock down. But they wouldn’t. This is some of the reasons motor riders knock pedestrian. Also, now there is no bus stop here anymore. When you are coming from Kumasi to Accra here. Bus don’t stop over here unless otherwise. But this is a place which is meant for bus to stop for passengers to alight. But cars don’t stop here. We have made demonstration; market women too have demonstrated but it did not work. Now they have blocked the bus stop with stones. Have you seen this? this concrete bar and others. So, what the driver do is to alight the passengers in the middle of road which sometimes causes accident in this area. When this happens like that a pedestrian crossing the road cannot see incoming vehicle and is being knock down by any vehicle.

Interviewer: What about Road conditions (such as potholes, lack of sidewalks), abandoned/broken down vehicles, over speeding, wrong overtaking, traffic.

- Participant: Over speeding too is a problem. All that I know is as a driver when you get to bus stop you have to slow down. But over speeding is over and ongoing.

Interviewer: What do you think decreases the risk of an accident?

- Participant: If accident can reduce then is the matter lies between the drivers and the pedestrians. If there is a police officer at this junction here, every blessed day in the morning enforcing the law there will be no accident.

Interviewer: Are there some people who are more likely to get into an accident (for example: children, hawkers)?

- Participant: Yes, we the hawkers who sell by the road side are those who mostly get into trouble. Just look, ahead of you is a car which crash some hawkers at the bus stop. But by the grace of God the fellow did not die. So, he said has fail break, so straight it knocks them about six people. Up to now some still have problem small, small. Also, those who normally sell in the traffic too it affects them.

Interviewer: Does it affect children?

- Participant: There is no child. But the last time, I witness an accident about a boy child. Who was crossing the road and suddenly was hit by a moving vehicle.

Interviewer: How old was he?

- Participant: About six years.

Interviewer: Sometimes personal stories can make road traffic problems more real. However, we know this can be sensitive. If you feel comfortable, can you share a story from an accident with me? Your own or someone else you know?

- Participant: Yes, I can, it all happened one afternoon when an elderly man of about fifty-three was crossing the road. He did not watch out carefully and was knock down by a car.

Interviewer: Can you tell me of a story about a child getting in an accident on the roads, if you have one?

Interviewer: Now, let’s talk about the police and their role.

Interviewer: What do you think about the police’s enforcement of laws now? For example, speed, motorcycle helmets, unlicensed driving, broken vehicles. Do you think this affects crashes?

- Participant: As I have already said, police those on the road. In Ghana our policemen are not many. If we have policemen on the road every morning there will be no accident. So, the policemen should come here to enforce the law by directing road traffic and also to enforce the law on pedestrians to use the footbridge.

Interviewer: what about over speeding?

- Participant: Over speeding too is going on.

Interviewer: Let me come in a bit. What about over speed, motorcycle helmets, unlicensed driving, broken vehicles

- Participant: Abandon vehicle is a major problem on our highway. They brought a law which says any abandon vehicle will be toe by a toeing car or track but it did not work. This causes a lot of accident. The last time there was an accident here. About last week, a driver abandoned his kia cargo car here in the middle of the road without a warning triangle. The driver said he had a problem. So, there was a saloon car coming towards that area. The saloon driver appears to the abandon vehicle on the spot. He decided to swerve and it then hit a moto rider causing accident in the area. Am the one who even use gentian violet to treat their wounds for them.

Interviewer: If you had the power, what would you do to change the situation here?

- Participant: What I will do is that, every blessed day latest by 5:30 to 6:00am a police officer will have to be here to ensure safety on the road. Especially the pedestrians crossing the road who happens to be the most cause of accident here. Because the drivers on the high way knows that there is no one there. Therefore, before he realized a pedestrian has cross him, causing accident. So, if am the one in power I will see to it that every blessed day latest by 5:30 to 6:00am a police officer will have to be here to ensure safety. If we are able to do that accident will not happen again.

Interviewer: Once an accident does happen, What do you think causes people to die or get hurt, compared to just getting into a crash without getting hurt? For example, what about the condition of the vehicle or trotro makes it more likely for a severe injury or death?Like seat belts not working in cars/trotros, cars being old and not having airbags, position of seats, crowding

- Participant: Some of the cars on the road are not good, talk of the tires, the seat and the entire body is not good. It does not deserve to be on the road at all. But because of economic hardship he’s still working with it. So, it is up to the police to check the car and inform the driver to do necessary maintenance on the car. Instead of the police arresting the drivers they will take a token from them and allowing them to go Scot-free. But the proper thing for them to have done is to arrest and seize the car. So as to avoid further accident.

Interviewer: Generally, which people typically to get injured or die in an accident? For example, pedestrians, children, motorcyclists, bicyclists, hawkers, those without a helmet, those who do not use seat belts.

- Participant: Over here I have not even seen young guy and girls been knock down by cars but the old men who are affected most. Sometime you will see an old man of about seventy years who is unable to walk properly but will be crossing the road. So, if there is a car on high speed who doesn’t know that there is someone crossing, he’ll definitely be knock down.

Interviewer: So, what about motor riders [who does not wear helmet] and hawkers?

- Participant: If a motor rider [okada] wear a helmet he protects himself but when he knocks down you will rather die. So, all this is an order from police service who has to control all these things, if not it will not work. Individual police men will come only to take money and go.

Interviewer: What about the environment (such as the roads) makes it more likely for a severe injury or death? For example, abandoned/broken down vehicles on the road, lack of sidewalks, potholes, traffic volume on roads

- Participant: Over here there is no problem with the road talk of bus stop is there for pedestrians, pedestrians walk way is there but the problem here is the crossing other than that there will be no knock down by cars.

Interviewer: What can be done to reduce the number of severe injuries and deaths here?

- Participant: The government has to stand its ground and make sure that every pedestrian uses the footbridge instead of crossing the highway. Please let me use a place like Madina as an example. Before the footbridge car use to knock people every day in and day out. But after the construction of the footbridge now it has reduced. Cars don’t knock down people because there is a police officer there who will not allow you to cross the road no matter how hard you try, you’ll not be allowed. So, these things they don’t care. If there is a police man here, he will enforce the law here by saying go back madam to use the footbridge. He will not allow you to cross the road for cars to knock you down but there is no police officer here nothing so every day people will cross the road. That’s , our problem.

Interviewer: When people get into an accident, or get hurt, what happens? For example, do people call the police? Do people come help? Does an ambulance come? Tell me about what happens.

- Participant: We the individuals over here when a car knockdown someone, people rush to the scene. if is a life body, we help them, but for dead bodies we detain for the arrival of the police. If is a life body we rush them to Achimota hospital.
- Participant: Yes, we call the police and they come.

Interviewer: When you call an ambulance, do they come?

- Participant: Master they wouldn’t come! Even if you call them, they would not come. As for me, I have not seen that a car has knock down someone for an ambulance to report to the scene before. I have not seen some before at overhead here before. Taxi drivers over here who convey the casualties to the hospital. There is no ambulance who come to Achimota here to say a car has knock somebody so am here to convey the injured peoples to hospital, never!

Interviewer: If you had the power, what would you do to improve care after an accident? For example, increasing number of ambulances, training people around in first aid

- Participant: Just as I said at the high way where accident is prone there should be one ambulance around. Like sport stadium where they have a standby ambulance, where something happens ambulance then responds. So, the road side which is busy they have to put one ambulance there. If it is there for 24/7. When something happens, driver is there to take you away. In the ambulance is first aid so anything at all that person can be save.

Interviewer: So, what about educating people around on the first aid?

- Participant: If we around have education on first aid, whenever there is an accident, we can help save life. there are some thing doctors do to revive people I will make sure in every area some will be there. Small clinic like chip compound, I will prove some at hot spot zones.

Interviewer: So, if you the government what are you going to do for people selling around like here?

- Participant: I will educate them all. People selling don’t have any knowledge about first aid so, the government will select people to come and educate the them. We will also educate the general public so that pedestrian crossing the road will be careful.

Interviewer: Over the past ten years, our country has recorded seventy-eight thousand accident and fourteen thousand deaths. For this reason, there is the need for us to find out the cause of these death. If you are the government, what are you going to do to reduce road accident.

- Participant: Government will come and talk but the matter depends on the drivers and good road. If we have good road there will be no accident and if the road is bad with a pothole’s accident will occur. If am the government I will the make sure all the roads are genuine. Cars on the road, are cars with a good tire. I will task some people to be checking those things. The person driving the cars does he have good training on how to drive? Where to stop and where not to stop. All these training the government has to bring it onboard for them to learn before they get on road to drive as a driver. If the person has all this knowledge when he gets on road to drive all these will not happen.

Interviewer: Does the government consider your views when they make decisions on road safety?

- Participant: NO

Interviewer: What is the government currently doing to reduce accidents? For example, speed bumps, enforcement by police, pedestrian bridges, education campaigns

- Participant: Over here in Ghana they will just say it but when they get the power, they don’t do it. when they are making their campaign and their rally, they have what to say but when they get the power, they don’t it. There are a lot of issues that need to be done right to help us but they will not.

Interviewer: So, haven’t you seen the government constructing foot bridge?

- Participant: They have done it. The one at Madina people lost their live before doing it.

Interviewer: Have you heard the government constructing footbridge elsewhere or education people on road safety.

- Participant: they do it. I sometimes hear it on television or on radio. But they should come to the field like here. This is the issue; this is the field let the police officer to come here. If I am here to cross the road and I see police officer here I be frightening and would not cross.

Interviewer: Why do you think the government chooses these speed bumps, enforcement by police, pedestrian bridges, education campaigns? Are they considered better? Are they cheaper? Do you think the government considers cost when they pick what to do?

- Participant: Yes, It’s better for the government to that. My brother. It necessary for them to do it. There are a lot of roads that need foot bridge but they will not do it.

Interviewer: Where do ideas about road safety come from?

- Participant: The knowledge is from us, what is happening to us is what we tell government. At first, I used to stay at La-Pass. Over there, is a footbridge but it is far from where we normally cross. At lapass, a part from flight top there is no footbridge there till Abrantieε. Looking at the people within that distance it is very far for them to use the footbridge. So, all of us cross the road instead of the footbridge. If should be a footbridge at Nii Boye junction at Lapass perfect. If there should be a footbridge at Lass-Palmas perfect but in all these areas there is no footbridge meanwhile cars do knock people.

Interviewer: Where do ideas about road safety come from? Do you think the government looks to other countries? Or at research?

- Participant; Yes, from the research you are doing. It would have been better for government in every road that he constructs there should have been a footbridge attached to it so that pedestrians will utilize it to avoid accident.

Interviewer: We know other countries use enforcement cameras, where people get a fine immediately if they speed or run a red light – do you think we can do such a thing in Ghana? Why?

- Participant: Yes, it will help. Because it will capture over speeding cars. Some of the pedestrians are their own cause. Some knowing an approaching vehicle on high speed, they will suddenly cross the road causing an accident. My problem is I don’t want to witness the scene of a car knocking down someone. Because if a car knockdown someone you can’t beer the behavior of that patient. So, if a driver knows that there is a speed camera a head of him that will catch him, the speed will reduce but he knows nothing is there to check him.

Interviewer: What mark will you give the government on a scale of 1-10 with 10 being the best?

- Participant: I will give him 4.

Interviewer: Why that mark?

- Participant Nothing is going on. We are dying, Ghana we are dying; every day accident, every month accident, every year accident people are dying too much. Else where in abroad do people die like that. People don’t die like that because we don’t listen to advice. The truth is there but they wouldn’t do it. Thank you [but when people die right now, they will come for the dead body]

Interviewer: Finally, our last question for you is, If you had the power, what would you do to reduce accidents, injuries, and deaths on the roads nationally? What would you do for pedestrians? What about motorcyclists? What about for children?

- Participant: The government and the Ghana Private Road Transport Union (GPRTU) has to sit down. The government should put his proposal on the table that am a new government who has come. I want to reduce the accident in the country. From now on your cars in you station its age is old so you have to remove it from the road. The road is small so am going to expand the roads. I will construct road for moto cyclist, where people will pass [pedestrians walk way], aya lolo [metro transport] it was plan that their root will be different, bicycle and the root for trotro [public transport] will be different, but did not work. If the government was serious on those initiative people wouldn’t have die like that. What is aya lolo? Participant these government green, green busses.If you go to Accra Tesano to be precises it is there, they plan that it will get it separate root but it did not work as plan. So, if am the government on power if the road is three, I will make it six lanes. On the same six lane, I will make a place for people to pass on its other side. Someone selling on the pavement is not right for people to do that because it was meant for people to pass, but because of system and economic hardship people are here to trade on the pavement. So, every day we have problem. Some people tell us their mind that here is meant for pedestrians to walking but not to trade. So, if am the government on power I will do where people will trade and where people will walk for peace to prevail. By so doing there will be no accident on us.

Interview: Is there anything else about crashes, injuries, or deaths on the roads that we haven’t discussed today that you would like to tell me?

- Participant: Ooh we have said it all but for me what is most important is education. For some people when they hear education issues on radio, they hear it for granted but if they are at the place where they hear that this morning GPRTU and Ghana police have come here to educate us on road safety so that accident will not happen. Within one week people will begin to realized that no this is really serious so, from hence I will not cross the road but to use the foot bridge. There will be peace, accident will not happen again.

Interview: Thank you for your time and participation in this important work.
