# Supplementary material for: “We are pleading for the government to do more”: Road user perspectives on the magnitude, contributing factors, and potential solutions to road traffic injuries and deaths in Ghana
Source: PLoS One. 2024 May 24;19(5):e0300458. doi: 10.1371/journal.pone.0300458 (PMC11125548; doi:10.1371/journal.pone.0300458)
Supplement: S2 File — (ZIP) [file pone.0300458.s002.zip › Transcripts to share/Participant_103_vulnerable.docx]

**Participant Number: 103**

**Language: English**

**Type of hot spot: Urban**

**Sex: Male**

**Road user type: Motorcyclist**

Interviewer: How do you usually get around in this area and what form of means to you use in this road?

- Participant: Yes I used this road often and we always start work around 7am and I used my motorcycle around this Achimota highways all the times. And sometimes you come and just be at the roadside. If a customer comes that he’s going to this place, then you make an inquiries. Where are you going to? The person will say, for example, I’m going to circle. Then you say, “with Circle we have this amount of money we take”. So the person will say, I’ll accept or might say, “Oh no, you have to reduce it to this!” so after everything, if we come to a consensus, then we take off the journey. We always charge the person and the person will also give us money for the journey.

Interviewer: Okay, so is this road a very busy road and how would you describe this area?

- Participant: Yes, is very very busy

Interviewer: How do you see the problem of accidents here, are they frequent?

- Participant: Yes, there is a lot of accidents that occurs here in terms of we those who are riding and those who are also walking for example you see here is not good for people to be crossing, esp this lane because there are a lot of accidents in this place and it concerns a motor rider and a person crossing the road and you see if you are crossing as much is a high way, as they are crossing they don’t always get time to wait for the cars maybe if they are not all that busy before they go in, they just cross and all those things happen But we those who are riding and drivers we do have a lot of problems because the drivers complain that motor riders we are disturbing them. And the disturbances here are; 1. If a taxi driver is to pick somebody maybe the person is going to pick a taxi, but because of the motor, you see the motor, sometimes there is no traffic on it. So, the person will prefer the motor over the taxi driver. So due to that, they are always annoyed with us that we are taking their passengers away from them. An incident happened yesterday, where a taxi driver just knocked down a motor rider. When the motor rider was to complain they started to split saliva on him. Which is very, very bad. And you see all those things can lead to a lot of accidents. Simply because, as that process, they were on the motorway, It’s not that they stopped. They were moving. So if we are moving this person is talking, this person is talking, we might miss up the way.

Interviewer: So, the accidents you made mentioned of, which people are more likely to get into an accident? For example, is it children, hawkers, passengers crossing the highway or who?

- Participant: Okay, those involve is mostly children and the over age for example 50 and 60 years old, the energy use to cross the road sometimes is not there so due that accident normally happen to them.

Interviewer: When you said children like how old or age category of children are you talking about?

- Participant: okay, like 5 to 10 year old when I started this business this is the children I normally see.

Interviewer: So do you have people around 15, 16, 17 18 years. Have you noticed any accidents with such people?

- Participant: No. When I started this business I have never seen anything like that.

Interviewer: Can you tell us if you have any personal story of someone gotten involved in an accident that you witnessed or some one witnessed and told you?

- Participant: Yeah yeah…. Personally I have experience this thing though it was myself that I had accident I collided with one person he was crossing the way but the fault was from me simply because I was on one lane, I was on the wrong lane, I was on the other lane trying to cross the traffic to this lane. So due to that, the person too you see, as we are moving from this way going, the person wouldn’t be concentrating or watching this side. The person would be concentrating on where the cars are passing. So, when I was just on the way, I was also focused on one car that was coming, so I didn’t realize the person was crossing. The person also didn’t watch the left-hand side of it so due to that we collided. And I got some wounds but by God’s grace nothing happen to this day. Not only that the speed that the cars and the motors we use on this highway is very bad, esp, I always say that if we are approaching the bus stop we should always try to reduce the speed, due to that an incident happened last two weeks on this way the motorway there is this heavy motors it is very very heavy he was on overspeed and there was an old man I think he would be 64 years he was crossing and the motor knocked the person down but we thank God the man is still alive.

Interviewer: Okay, can you tell me the story about the child?

- Participant: Yes, that one the problem was from the mother because if you are crossing the road with your child you have make sure that you hold the person hand or you let the person be at the back of you, not even the back, at the side ways of you, so that, as much as you an elder you will turn left and right to see what’s going on before you cross. So that very day the mother left the child and cross when the mother cross the child was also eager to follow up and car knocked the child down.

Interviewer: Thank you now let talk about the police and their role. How do you see their enforcement of laws regarding speed, helmets and license checking and broken vehicles on the road?

- Participant: Yes, the police they do it is only the speeding that they don’t do concerning the speeding, as for helmet, if you are not wearing helmet you will have a problem with them, if you don’t have license or you have not registered you will have a problem with them yes immediately they detect it they will take to the police station and if you go there you have to proof with your documents before they released your motor vehicle. But as for over speeding side I have not notice anything concerning the police towards it or putting measures to.

Interviewer: So, what the police are supposed to do and they are not doing do you think this affects the number of crashes here?

- Participant: Okay, it doesn’t reduce the number of crashes here, the reason why I said it doesn’t reduce is, it is only the helmet and other things license that they do check, you understand and it is only that they have something they called operation they always have a specific day that they use to check up. But on a highway, some of the police they are on the traffic, they regulate the traffic for us, even if there’s a lot of traffic on the way. Because of the light sometimes it depends on the other side of the road. So if the other side is busy they will always regulate the other side for movements to be free and that is how actually they do here.

Interviewer: So if you have the power, what would you do to reduce the number of road accidents here or what would you do to change the situation here?

- Participant: If I have my own power I would rather put some small speed rumps or some signs you see there are some speed rumps signs as a driver is getting to and see that sign automatically the speed reduce you see… even if they don’t put any speed rumps but they put those signs as much as the driver is speeding and getting to that sign and see this sign is there will be some kind…. of the speed will reduce.

Interviewer: So if there is an accident that happens here, what happens after the accident? For example does it lead to people getting injured or leads to death?

- Participant: Yes, there are a lot of injuries, if there is an accident. But we haven’t experienced death situations.

Interviewer; What do you think are the things that causes people to die or get injured pertaining to the condition of the car like presences or absences of seat belts, seat belts position, crowding or what?

- Participant: Yes, No, it was once a car got spoiled on the way and applied breaks at once so those were at the back actually there were a lot of collision or is crashes yes and some bodies were not all that…. But passengers inside mostly were safe.

Interviewer: What about seatbelts? Do they have seatbelts in the vehicle and do people wear them?

- Participant: Ok with the Trotro, it’s only those in front that have seatbelts but those at the back have no seat belts for them, we those at back in the trotros don’t have seat belts to wear, but in front have and you see because it is short distances sometimes we don’t always regard the seat belts yeah.

Interviewer: If people typically get into an accident, which category of people are mostly affected? For example, is it pedestrians, children, motorcyclist, or hawkers? Is it also those with helmets or without helmets or those who doesn’t use seat belts?

- Participant: Okay, people that are crossing the road, those that are always crossing the road

Interviewer: How about the road environment like potholes and others?

- Participant: Okay, yea there is some side ways that has potholes for example there is just one here it do disturb, some cars they will be coming as it is moving with the speed they don’t realize it by the time they realise it is over and also if it rains water always covers it and you don’t see the potholes so immediately the driver passes on it water always splashes all over so those who around always feel the pains that the dirty water is on them.

Interviewer: So do you have sometimes abandoned vehicles on the road here that leads to traffic and then leads to accidents?

- Participant: Yes, it always lead to traffic esp if…not abandoned vehicles rather but vehicles that got spoilt on the way. Because some of drivers they always try their possible best to move the vehicle away from the road but still is always difficult for them.

Interviewer: So, looking at this situation what do you think can be done to reduce the number of severities and deaths here?

- Participant: Left on to me we need some kind of speed rumps or there is some rope, I don’t know what name they call it but there is rope it is always heavy so if they put it across it helps.

Interviewer: Whenever an accident occurs here what happens? Do you call the police? Do people come to help? Does an ambulance come? Tell me what happens?

- Participant: Yeah, if there is an accident the first thing we always do we those around we always try to rescue the one involved the victims so if we get into it we then call the police some of them the police are not far from us because every traffic light there is a police there so they always come those things for example if it is a motor they will just pick the motor to the police station and if it is a car too they will also do that and then follow up. For example, One time an accident occurred where a motor collided with this heavy truck. So the motor driver was lucky. That very day the police men were there and one of them escorted the victim and the driver to the hospital. So that very day I was happy about what the policeman did.

Interviewer: Do they call the ambulance? Do the ambulance come and how long does it take for them to come?

- Participant: The ambulance side I have never seen that if not a very heavy accident that they do but those minor accidents I have never seen them call an ambulance but if maybe there are some two cars that collided and they is a lot of injuries and deaths and they involve the ambulance service.

Interviewer: If you call the ambulance, do they just come because you have called them, or they are looking at the caliber of the one that call them or the vehicle that call them or they just come?

- Participant: If it is a urban place they are always quick and faster but rural place sometimes it take time simply because I experienced that one time but by the time I was in Western region.

Interviewer: If you had the power, what would you do to improve care after an accident has occurred? Will you be increasing the number of ambulances or training more staff to give first aid?

- Participant: Yes, if I have the power what I would do is the ambulance should always be ready for any accident that occurs maybe they need immediate treatment you understand and but maybe you called them and they will delay there is no first aid they do give them before the ambulance will come, you see we have some hospitals that are closer to this place so immediately there is an accident they will just organized to pick the victims to the hospital. So due to that, those types of first aid, or somebody, maybe in an office has seen that this has happened so let me send first aid. No, I have not experienced that.

Interviewer: In your opinion and your own estimation are accidents a much problem in Ghana?

- Participant: Yeah yeah… it is a very big problem and you see if…. The reason why I said it a problem is if an accident occur and you are not of good condition for example the government don’t do anything to you again for example you had an accident and your leg got broken or they cut off your leg or your arm you have now become a disable person and at that point instead of you to... this is now the duty of ~~productive it is now~~ the social welfare to take over and to take care of you by even paying your hospital bills and other things but sometimes they don’t.

Interviewer: So, looking at all these, do government consider your views when they make decisions on road safety.

- Participant: Okay, I have never experienced them moving around taking decisions from we those who are motor riders or taking decisions from the vulnerable people and you see mostly ladies are vulnerable they do say it and it is true you understand aaahaa!... sometimes instead them to move round and take decisions or information or opinions from we those who are riding I have never experience that.

Interviewer: For the past 10 years, we have had 78,000 number of crashes in Ghana, and out of it, we have 14,000 people dying. So I just want to find out from you, what do you think the government is doing to reduce these accidents?

- Participant: OK. To improve…the government…right? I can’t speak for the government. But what I am thinking is, they should rather concentrate on the speedbumps and working on the roads. The road too sometimes disturbs, especially if it rains. I can testify last week Friday, there was a lot of traffic, I don’t know it looks like something happened. So the traffic even went up to 12:00 AM. And they were still cars trying to get to their own places and it was difficult for them. Because it rained that very day heavily. So sometimes, if it rains, it brings a lot of problems to the riders and the drivers.

Interviewer: And you mention speedbumps on the road?

- Participant: Speedbumps on the road, yeah, yeah, yeah, yeah, yeah. If there’s a lot of speedbumps, it will help to improve or to prevent, or to reduce accidents.

Interviewer: And then do consider those speedbumps to be kind of better?

- Participant: Yeah, they have a different type of speedbumps. But me, I don’t like the steepy ones. Yeah, there’s some speedbumps that are always nice. You see, they will do it in a safe form. So as we are claiming, we are climbing it slowly. But there are some, as you are to climb it, it’s always difficult.

Interviewer: Do you think government considers cost or what? So, you said some of the speed rump here are not good, and what do you think government decides to do speed ramps in some places and not all places?

- Participant: Yeah, I think is because of the cost because those things are not bigger as compare to normal ones. Those ones are just small small and I think those ones are less cost to the government that’s why maybe they prefer that.

Interviewer: So, this question just answer according to your ability, where do government get their ideas on road safety? Is it that they look to other countries or at research?

- Participant: Hmmm ok I think those information or ideas they get it from scholars especially those who are into road safety.

Interviewer: In some countries they use enforcement cameras to monitor the speed of vehicles on the road, and people get fine immediately if they over speed or run a red light- do you think we can do such a thing in Ghana?

- Participant: Yes, if there is a lot of them in Ghana it will help because certain things just happen even last… the day before yesterday. The driver speed over the traffic light and he move maybe 15 minutes and the police were having some directions to get the driver that he has by passed the traffic without obeying it so I think there were some sanctions to the driver and it is helping so if there a lot of cameras to detect those thing it will really help.

Interviewer: What mark will you give government on a scale of 1-10 with 10 being the best and 1 being the poorest? What mark will you give government?

- Participant: Okay, in Ghana here from 2010 to 2022 I think I will give 7, the reason is still much to be done in terms of safety. They should put much more in terms of road safety. Because if you said, they are on a higher point that it is okay for us in Ghana, it will be difficult ~~Because if we want to say we in higher in road safety point or is okay for us it will be difficult~~ because the speed and the road network is very bad yeah especially in the rural settings… in the rural settings is very bad in terms of the road network so due to that I cant rate Ghana road system as 10.

Interviewer: If you have your own power, what will you do to reduce accidents, injuries, and deaths on the roads in Ghana? What will you do for pedestrians, motorists and children?

- Participant: On to me, people that are crossing the road we should always have overheads, yah! We should have a lot of overheads for people and you see some people don’t prefer the overheads that is the reason why the accidents occur, they like short short things so if the government do it in such a way that it wouldn’t take long for them to cross and you see some of people are also weak, climbing the stairs to cross up is also difficult or even climbing the slopy overhead is difficult for some of them so due to that they always prefer to use the main road to cross. Because there is not this thing… our road network is not good left on to me the finance we put in to the road system is bad left on to me we should have more motorways just close to the main road so that the cars will be passing differently and the motors will be passing differently. Motors we are not all that plenty so if they are going to do it they shouldn’t expand it just make it like a foot this thing so that if this side is having one then the other side is having one it will help to reduce a lot of accidents. With the children, that one we should put the blame on their parents. Parents should try their possible best to take care of their children not to be crossing the road any how or if they are crossing they should be there to guide them.

Interviewer: Is there anything else that you want to add to this our conversation regarding crashes, injuries, and deaths on the road that we haven’t talked about today?

- Participant: Okay, what I have to say is you see all those riding left on to me they should have some law enforcement to put it on to them that as much as you are riding and you are getting to a bus terminal you should always put your speed down that is the reason why I was saying every bus terminal should have a speed rump two or three it will help and it will try to reduce the number of accidents.

Interviewer: Thank you for your time and participation in this important work, I thank you so much your patience.
