# Supplementary material for: “We are pleading for the government to do more”: Road user perspectives on the magnitude, contributing factors, and potential solutions to road traffic injuries and deaths in Ghana
Source: PLoS One. 2024 May 24;19(5):e0300458. doi: 10.1371/journal.pone.0300458 (PMC11125548; doi:10.1371/journal.pone.0300458)
Supplement: S2 File — (ZIP) [file pone.0300458.s002.zip › Transcripts to share/Participant_104_vulnerable.docx]

**Participant Number: 104**

**Language: Twi**

**Type of hot spot: Urban**

**Sex: Female**

**Road user type: Pedestrian**

Interviewer: How do you usually get around? How do you get to work? Do you use this road often? For example, walking, public transport (trotros), motorcycles, cars, taxis, trucks, riding a bike, tricycles (i.e., pragya)

- Participant: From my house to this place is not far so I walk.

Interviewer: Over the past ten years this area has recorded one hundred and two accidents, out of that figure twenty-four people have died. So, this is why we are here to find out the cause of these accidents. How would you describe this area to others? Is this road busy?

- Participant: Yes, but not much once in a while.

Interviewer: How big of a problem do you think accidents are here?

- Participant: Is a big problem to us. Some of the drivers drive carelessly without care and on high speed too. Also, the pedestrians when crossing the road instead of them to run they will not run but will be walking that is some of reasons for accident to occur in this area.

Interviewer: What do you think causes accidents here?

- Participant: Ok most of the accident is the fault of the drivers because they come with speed, so before the person will realize to run the car has already close up on him. The people too when they are crossing the road they don’t run. That is what causes accident.

Interviewer: Road conditions (such as potholes, lack of sidewalks), abandoned/broken down vehicles, over speeding, wrong overtaking, traffic

- Participant Please yes, especially when a car breaks down in the middle of road and you are crossing the road and you don’t watch your back well or get closer to the abandoned car well before you make your intention to cross the road there will a car coming because it has blocked your view. So, if there exist an abandon car on the road it is likely that car can knock you down, because it has blocked your view. So, if you don’t take care that one too can cause accident.

Interview: What do you think decreases the risk of an accident?

- Participant Over here, ok, there is pedestrian bridge and also there exist a staircase that if you want cross to another lane, you can climb and go. To me it is the best way for pedestrians to be safe from accident.

Interviewer: Are there some people who are more likely to get into an accident (for example: children, hawkers)? Which age of children?

- Participant: Over here children mostly cross the road. So, children are the likely ones. Over here, just last month a motorcycle knocked down a child of about nine to ten years. Again, just exactly a week after that another baby was knocked by a motorcycle over here.

Interviewer: what those who sell along road side (hawkers)

- Participant: Those who sell here have never been involved in an accident before.

Interviewer: Sometimes personal stories can make road traffic problems more real. However, we know this can be sensitive. If you feel comfortable, can you share a story from an accident with me? Your own or someone else you know?

- Participant: O, several times.

Interviewer: can you tell us?

- Participant: Last two months a petrol tanker knocked down two people (pedestrians) just in front of me. It run over one of them to death instantly. But the other one who was taken to the hospital died in the hospital the next day. The incident happened on Wednesday but on that day the one who was overrun by the car died on the spot. But the lady among the two with severe injury died the next day which was Thursday at the hospital. And last month too, a motorcycle knocked down two people. And this month too, motorcycle knocked down someone here. So, for accident, I have seen a lot.

Interviewer: Can you tell me of a story about a child getting in an accident on the roads, if you have one?

- Participant: Yeah, that is nine to ten years, a little girl.

Interviewer: Now, let’s talk now about the police and their role. What do you think about the police’s enforcement of laws now? For example, speed, motorcycle helmets, unlicensed driving, broken vehicles. Do you think this affects crashes?

- Participant: Over here, talking about the police, to be frank they don’t come here to help people to cross. Unless may be after a car has knocked down someone and maybe they are passing by that you will see them helping out to convey the injured person to the hospital. Apart from that you will never see them on duty helping people to cross the road or protecting the pedestrian.

Interviewer: Example, over speed, motorcycle without helmets, unlicensed driving, broken vehicles. Do you think this affects crashes?

- Participant: Please yes

Interviewer: By what way does it affect crashes?

- Participant: Talk of the helmet, we know that helmet protects motor riders a little bit when they crash. Also, to the drivers, when you are driving at least they should wear their seat belts. When we talk of license, I know a lot of them don’t have license.

Interviewer: Over the past ten years this area has recorded one ninety- two accidents, out of that figure twenty-eight people have died. So, this is why we are here to find out the cause of these accidents. If you had the power, what would you do to change the situation here?

- Participant: To me, if you will cross the road, it’s better to climb the overheard (pedestrian bridge) that will help because if there is no overheard then I will say that there is no option but there is an overhead (pedestrian bridge) here so if and only if you will cross the road, the best is to climb it.

Interviewer: Once an accident does happen, what do you think causes people to die or get hurt, compared to just getting into a crash without getting hurt? For example, what about the condition of the vehicle or trotro makes it more likely for a severe injury or death? If there is a crash among car what causes people die or injured. Like, seat belts not working in cars/trotros, cars being old and not having air bags, position of seats, crowding.

- Participant: It may be that a glass will break to cut someone onboard, the car will be crumple to affect the passenger, maybe the affected area might not be good for that person, maybe if it affects the head, he might get internal bleeding that one too is part of the reason for which accident occur.

Interviewer: Let me come in a bit seat belt not working in cars/trotros, cars being old and not having air bags, position of seats, crowding seat

- Participant: Most of the accident are as a result of negligent of some driver because some of the drivers will not check their cars before they move it. Another reason is that some of the cars are very old and have weak body part. To the extent that even bare hands can break some of its part, not to talk of its seat or seat belt. In some cars, the seats are very close to each other so a little crash passengers die on the spot.

Interviewer: Generally, which people typically to get injured or die in an accident? For example, pedestrians, children, motorcyclists, bicyclists, hawkers, those without a helmet, those who do not use seat belts

- Participant: Most of them are Pedestrians ~~crossing~~

Interviewer: Were some children?

- Participant: Yeah, there were children among the casualties.

Interviewer: About what age?

- Participant: children of about nine, ten, thirteen, fourteen years.

Interviewer: What about the environment (such as the roads) makes it more likely for a severe injury or death? For example, abandoned/broken down vehicles on the road, lack of sidewalks, potholes, traffic volume on roads

- Participant: Ok, over here there are no potholes and the road is good too.

Interviewer: What can be done to reduce the number of severe injuries

- Participant: If we get government people like police here to see to the people crossing the road it would have been helpful. Maybe while the person is crossing the road and there is a car on the high-speed coming, the moment he sees a policeman he will reduce his speed for the people to cross. But he would not mind me if a normal person like me without a uniform stops him, he will not. Also, if you climb the overhead, accidents will reduce.

Interviewer: When people get into an accident, or get hurt, what happens? For example, do people call the police? Do people come help? Does an ambulance come? Tell me about what happens.

- Participant When accidents happen, it is those by the road side or the hawker who call a taxi to convey the injured person to the hospital. But as to call for police or ambulance here, I haven’t seen some before over here. The taxies, even when you stop some of them it is difficult. Even to get one who will stop for you in times like that is something else.

Interviewer: So, the police don’t come and ambulance too don’t come.

- Participant: Yes, they don’t come. For police, maybe only when they are passing by that they see to stop.

Interviewer: When you call an ambulance, do they come?

- Participant: NO, they don’t come. I’ve never seen any ambulance here after accident before.

Interviewer: Ok looking at this place what do you think can reduce the number of accidents, the nature of the car, the nature of the road. What do you think about the cars and the road when done will reduce accident. Over here at Achimota.

- Participant: If we get government appointee like police here to direct people to cross the road it would have help. May be while the person is crossing the road there may be a car coming if the police man stops him, he will stop but if a normal person like me, without uniform stops him, he wouldn’t stop. But police can make the driver reduce his speed for the people to cross the road. Or if you use the over head it will reduce the accident.

Interviewer: You said when you call ambulance they don’t come when you call police too, they don’t come. If you had power where the police are under your control ambulance is under your control, how to train people, giving knowledge to people around about road accident and care after accident. For example, increasing number of ambulances, training people around in first aid.

- Participant: Oooh, What I would have done is, if you are under my command, it’s a command, it will be forced on you to do it because it’s your duty to do it. So, if you don’t do it, I will sack you. Yeah.

Interviewer: What about the ambulance, increasing their numbers?

- Participant: Ambulance is there to carry sick people or accident casualties so knowing that be your duty you shouldn’t delay, if you delay it’s against the law.

Interviewer: Now that they don’t come if you have the power what would you do?

- Participant; I will sack them.

Interviewer: What about educating those around the road side?

- Participant: Ok, we those around here we work on our own but I will portion a blame on you a bit because if your friend has gotten in to a problem its your core duty to help that person because man ought to help one another. It’s not only government worker who ought to do that. So, you too if your brother is in trouble, you have to get up and help him.

Interviewer: In your opinion, how much of a problem is accidents in Ghana?

- Participant: hmm issues about accident hmm, it’s been occurring, It’s worse.

Interviewer: Accra, Takoradi, Kumasi, Tamale is accident a problem?

- Participant: It’s worse especially over here in Accra. Even lapas here if you find out from them you will hear more than mine. It’s not good, it’s very bad.

Interviewer: Does the government consider your views when they make decisions on road safety?

Participant: Hmm, as for them, if you say, you will wait for them to come before you act hm! Ha-ha then before they arrive that person would have been a dead body. Because for them hmmm.

Interviewer: If you voice out, do they listen?

- Participant; Even if they hear at all, their responds wouldn’t be fast as it’s supposed to be. Maybe what I know is whenever any incident happens since it’s your work and responsibility it has be rapid respond. But for them they are not good with that.

Interviewer: What is the government and road safety authority and those in charge of road transport what are currently doing to reduce accidents in Ghana? For example, speed bumps, enforcement by police, pedestrian bridges, education campaigns Have you heard of those?

- Participant: Yes, I have heard of it

Interviewer: Have you seen those?

- Participant: Yes

Interviewer: Where do ideas about road safety come from? Do you think the government looks to other countries? Or at research?

- Participant: Ok, that knowledge, they get it from another country, yeah because when they realize that this country whatever they are doing, maybe helpful in our country. They have to adopt that. So, most of the ideas they get it from different country.

Interviewer: Also, what do you think about research that we are doing?

- Participant: Yes

Interviewer: We know other countries use enforcement cameras, where people get a fine immediately if they speed or run a red light – do you think we can do such a thing in Ghana?

- Participant: Yes

Interviewer: Why?

- Participant: It will be helpful because if someone does something bad it will capture that person and trace to his arrest. I over heard that some is here but if indeed it is functional then the accident which have been happening here, they would have seen it. But it not working. so, if we get some here it will be helpful because if someone knock somebody down it would have help. Most drivers after knocking down people they don’t stop but run away so if the speed camera is here, it will capture their number plate and when we trace their number, we will see it.

Interviewer: Over the past ten years, our country has recorded seventy-eight thousand casualties and fourteen thousand deaths in an accident. For this reason, there is the need for us find out the cause of these death. What mark will you give the government on a scale of 1-10 with 10 being the best? Why that mark?

- Participant: I will give them one because they are not performing.

Interviewer: Finally, our last question for you is: If you had the power, what would you do to reduce accidents, injuries, and deaths on the roads nationally? What would you do for pedestrians? What about motorcyclists? What about for children?

- Participant: Ok, If I have power, I will stop people crossing the road.

Interviewer: What about moto riders?

- Participant: For moto riders without helmet will be arrested.

Interviewer: What about children?

- Participant: The children too I will make may be the police men to sack the children from crossing the road so that they will not be close to the road. Though some of the children are very stubborn if you don’t do that, they will cross the road, they can pass behind you, even after you have sack them, they can pass behind you to cross the road. So, if there is someone like police man here to sack those stubborn children from crossing the road.

Interviewer: Is there anything else about crashes, injuries, or deaths on the roads that we haven’t discussed today that you would like to tell me?

- Participant: NO, THANK YOU.

Interviewer: Thank you for your time and participation in this important work
