# Supplementary material for: “We are pleading for the government to do more”: Road user perspectives on the magnitude, contributing factors, and potential solutions to road traffic injuries and deaths in Ghana
Source: PLoS One. 2024 May 24;19(5):e0300458. doi: 10.1371/journal.pone.0300458 (PMC11125548; doi:10.1371/journal.pone.0300458)
Supplement: S2 File — (ZIP) [file pone.0300458.s002.zip › Transcripts to share/Participant_105_non_vulnerable.docx]

**Participant Number: 105**

**Language: Twi**

**Type of hot spot: Urban**

**Sex: Male**

**Road user type: Driver**

**PARTICIPANT#105**

Interviewer: How do you get to work?

- Participant: If I get up in the morning not less than 5:00 I have to be here.

Interviewer: Do you come with a taxi, public transport (trotros), motorcycles, cars, trucks, riding a bike, tricycles (i.e., pragya)?

- Participant: I board a car.

Interviewer: How would you describe this area to others? Is this road busy?

- Participant: Yes, very busy.

Interviewer: How big of a problem do you think accidents are here?

- Participant: Over here in our station we move from Accra to Kumasi. If we drive towards Kumasi roads sometimes accident occur, and every accident that occurs are very serious. some of the accident fatal, some loses their life, some get injured and their injury too is serious.

Interviewer: What do you think causes accidents here? Road conditions (such as potholes, lack of sidewalks), abandoned/broken down vehicles, over speeding, wrong overtaking, traffic.

- Participant:: Over here, since the roads slopes it’s the public transport that fails brake and will crashes the pavement. Most of the accident that happens on the road is the fault of the drivers who don’t seek to proper maintenance of their cars and not being conscious on the road. Also, some of the accidents are as a result of potholes both drivers trying to dodge then eventually they crash. Some accident is cause by a big pothole on a high when the car falls in it and then it veers off the road, then eventually crash with someone or fall off the road.

Interviewer: What do you think decreases the risk of an accident?

- Participant: What we have do is to be patient and conscious on the road. Over here is a roundabout, if someone is moving you ought to be patient for him to move, the one in the roundabout go before you the one joining the cue to join. Sometimes we don’t get that patience, everyone is in a rush especially in the morning. So, that causes accident here.

Interviewer: Are there some people who are more likely to get into an accident (for example: children, hawkers)? Which age of children?

- Participant: ooh! Over here, because of rushing, it’s the drivers that has been hitting each other’s cars. Sometimes the car will hit others rare(bumper), someone too will scratch other sideways(fender), and so on. Car don’t frequently knock people(pedestrians) like that, it’s not many but sometimes it’s motor bike that may knock someone (pedestrians). Since I came I ‘ve never seen that a car has knock down someone over here before.

Interviewer: Sometimes personal stories can make road traffic problems more real. However, we know this can be sensitive. If you feel comfortable, can you share a story from an accident with me? Your own or someone else you know?

- Participant: sometimes, some usually happens because It’s about two years ago, some car like Nissan urvan (public transport) failed brake and then crash the roundabout for the car to summersaulted. People got injured but non died.

Interviewer: which age category of passengers that got injured

- Participant: ooooh! about two girls got injured and one man under the age of thirty-five to forty years.

Interviewer: Now, let’s talk now about the police and their role.

- Participant: Because of the morning and evening traffic jam, the police come around especially in the morning. They also come some times in the evening to regulate traffic intermittently. So, if every time police comes here, it helps reduce accident.

Interviewer: What do you think about the police’s enforcement of laws now? For example, speed, motorcycle helmets, unlicensed driving, broken vehicles. Do you think this affects crashes?

- Participant: Yes, they contribute to the numerous of accidents on our roads. Because in our country almost all the cars, trotro (public transport) and the rest about sixty to seventy (60-70%) of our cars on the road are old. The driver is hot and so we don’t do maintenances. The car owners will say to the drivers you just manage it small-small by time you realized there is a crash.

Interviewer: If you had the power, what would you do to change the situation here?

- Participant: I will bring a lot of police men here because at the round about the traffic always become intense. When it happens like that car coming from Nsawam towards the roundabout get stacked, so, the police have to regulate the traffic for it to be free from jam and also to enforce the law on the drivers.

Interviewer: Once an accident does happen, What do you think causes people to die or get hurt, compared to just getting into a crash without getting hurt? For example, what about the condition of the vehicle or trotro makes it more likely for a severe injury or death? Like seat belts not working in cars/trotros, cars being old and not having air bags, position of seats, crowding

- Participant: As you are saying seat belt is important but as for trotro, (public transport) passengers alight at a regular interval so that is why there is no seat belt. Talking about the cars, it is true that some of its seats are too close to each other. So once there is a crash some passenger might be frightened to move forward there by getting a slight cut on any part of their body part

Interviewer: Generally, which people typically to get injured or die in an accident? For example, pedestrians, children, motorcyclists, bicyclists, hawkers, those without a helmet, those who do not use seat belts

- Participant ooooh talk of accident we don’t really have any specific people to say adult or elderly people die or children die. Sometimes a child of about five years may die. Sometimes young male and young female.

Interviewer: Does it mostly affect motor riders without helmet, children, or we those walking by the road side, those who trade by the road side or those without the seat belt. which of them get injured or die in an accident.

- Participant: Since this place slopes and busy, when the car fails brake from up there, it makes the car knockdown women who trade around (hawkers) and the people crossing the road (pedestrians) may be knock down by that car. So, we don’t have any specific people who get affected by accident. Because last time, a car fails break and a man passing by who has gotten down from his bicycle pushing it across the zebra crossing suddenly over run by that car to his death at the zebra crossing while crossing the road. He died on the spot.

Interviewer: Over here has any child involve in an accident before

- Participant: O! NO

Interviewer: What about the environment (such as the roads) makes it more likely for a severe injury or death? For example, abandoned/broken down vehicles on the road, lack of sidewalks, potholes, traffic volume on roads. What about the road

- Participant: Over here our road is not good. If you are from Pokuasi to this roundabout there is a big hole here, especially at down when town is quiet about 4:00 to 6:00am. Some of the cars, when they get there they don’t know that there is any bump there, if you go there, you see it right now. So, the bumping sometimes veered them off the road towards the fuel station.

Interviewer: what about broken down vehicles?

- Participant: It’s true that abandoned cars too cause accident especially those without warning triangles. The reason is that if you are driving at night and you happen to reach at it on the spot hmmm it becomes a matter between you and your God. Our road too is not good. Just go and witness with your eyes over there. The zebra crossing for pedestrians has been block with barbwires. So, if pedestrian what to cross they have to cross through to the roundabout.

Interviewer: What can be done to reduce the number of severe injuries and deaths here?

- Participant: We have to get road signs on our roads and every driver drives with a road sign. Some of the road signs too, many of them are no more. When driving at night you can’t see ahead of you, and when driving whiles raining is worse. We have to get police men to enforce laws here.

Interviewer: When people get into an accident, or get hurt, what happens? For example, do people call the police? Do people come Help? Does an ambulance come? Tell me about what happens.

- Participant: When accident happens, we converge at the scene. some body will just pick up his phone and then call the police. When it’s serious we put them in a taxi or trotro and take them to the hospital. Sometime before the ambulance arrives, we have already taken all of them to the hospital.

Interviewer: When you call an ambulance, do they come?

- Participant: Yes, they come. Some of the police too will come for the dead bodies.

Interviewer: How long would an ambulance take to arrive?

- Participant: I don’t know the specific time.

Interviewer: Who gets an ambulance and who doesn’t? For example, does it depend on if you are in an urban or rural area? Or the conditions of the road? Or if it’s a major road and it causes congestion?

- Participant: No, no, no, they only do their job and go. Even some of the casualties we don’t know them but they care for them too.

Interviewer: If you had the power, what would you do to improve care after an accident? For example, increasing number of ambulances, training people around in first aid.

- Participant: Since this place is busy if we get ambulance as a stand by, it will help. So, I will bring one here. I will also educate the general public especially the people around here because we do not have any knowledge about emergency care. After accident how to care for the injured person while suffering is a problem. Whether to hold the hands, the legs, the head we don’t know, whichever place that we get then we hold to help that person, in short, the knowledge is completely not there. So, if we get someone who will educate us on that by teaching us that if you happen to meet an accident and someone is suffering hold his, hold his middle body or his leg all that we know is to hold him that’s all.

Interviewer: That why am saying if you have power what will you do?

- Participant: What I will do is, I will educate hit the ground, all is about education, we will learn, I will educate them that if something happens do it like this nor like that just do it right as it is.

Interviewer: In your opinion, how much of a problem are accidents in Ghana?

- Participant: The matter of accident is terrible and it on going canker and it’s occurring too much.

Interviewer: Does the government consider your views when they make decisions on road safety?

- Participant: The government in particular has people who works under him but sometimes they don’t listen to us; because from here to Kumasi the most part of the road is not good, a lot of speed bump which not good.

Interviewer: What is the government currently doing to reduce accidents? For example, speed bumps, enforcement by police, pedestrian bridges, education campaigns Have you heard of those? Have you seen those?

- Participant: Yes, I sometimes see road safety commission people around but it is not up to the task. They are active when getting closer to Christmas and Easter holidays. Meanwhile they are supposed to hit the ground in every two or three months through the year. They should come to the lorry station to educate us, they should come to the market to teach us, if you are crossing the road look here nor there. If you are in a car and the driver is over speeding find a polite way to talk to the driver that driver, we beg slow down for we shall soon get to our destination. So, therefore, they should hit the ground, go to the field to educate the general public on road safety.

Interviewer: Why do you think the government chooses these? Are they considered better? Are they cheaper? Do you think the government considers cost when they pick what to do? Where do ideas about road safety come from? Do you think the government looks to other countries?

- Participant: The government, the government since old days has road safety commission is there, we people who design and come out with the ideas about rode safety. The government has people who have been train on road safety issues. So, where government get his ideas, I don’t know.

Interviewer: So, from all that I said where do ideas about road safety come from? Is it from the research that we are doing? or from another country?

- Participant: I think may be as we are talking government listen. Sometimes they have been travelling a lot, the government travels so there is saying that someone learn wisdom from the other. May be as they travel to other countries, they learn from them too. Research too is part of their sources.

Interviewer: We know other countries use enforcement cameras, where people get a fine immediately if they speed or run a red light – do you think we can do such a thing in Ghana?

- Participant: Yes, it will help a lot.

Interviewer: Why?

- Participant: This will help because some passengers when onboard will be worrying drivers by yelling at them by saying we are late, be hurry to speed up. When they do the driver can prompt them that there is a speed camera there if I speed beyond the limit I will be arrested. So, the driver wouldn’t speed up and the passengers too wouldn’t trouble the driver by saying am in hurry so hurry up. This will caution drivers to be careful when they are speeding.

Interviewer: What mark will you give the government on a scale of 1-10 with 10 being the best? Why that mark?

- Participant: I will give him four because little has been done on rod safety. As am saying when it’s not Christmas you don’t see road safety around. No one come around to teach us so, I will give him four. Education on road safety is too low.

Interviewer: Finally, our last question for you is, If you had the power, what would you do to reduce accidents, injuries, and deaths on the roads nationally? What would you do for pedestrians? What about motorcyclists? What about for children?

- Participant: First of all, the children who has been crossing the road, I will send knowledge of road safety in the educational curriculum. Teach them only to walk on the pavement [pedestrian walk way] if possible, will fens the portion of road they ‘ve been crossing especially town or cities with barbwire. So that all will pass the proper place. Zebra crossing too we have to get someone there morning till evening running shift. Who will be stopping car for pedestrians to cross. Every car which goes on a long journey. I will put machine inside the car to limit their speed, if you speed beyond that limits it will not go. I will also fix cameras in town to check speed limit.

Interviewer: Is there anything else about crashes, injuries, or deaths on the roads that we haven’t discussed today that you would like to tell me?

- Participant: NO THANKS

Interviewer: Thank you for your time and participation in this important work.
