# Supplementary material for: “We are pleading for the government to do more”: Road user perspectives on the magnitude, contributing factors, and potential solutions to road traffic injuries and deaths in Ghana
Source: PLoS One. 2024 May 24;19(5):e0300458. doi: 10.1371/journal.pone.0300458 (PMC11125548; doi:10.1371/journal.pone.0300458)
Supplement: S2 File — (ZIP) [file pone.0300458.s002.zip › Transcripts to share/Participant_106_non_vulnerable.docx]

**Participant Number: 106**

**Language: Twi**

**Type of hot spot: Urban**

**Sex: Male**

**Road user type: Driver**

Interviewer: Do you use this road often?

- Participant: Yes

Interviewer: Then tell me how you usually get to work? For example, walking, public transport (trotros), motorcycles, cars, taxis, trucks, riding a bike, tricycles (i.e., pragya)

- Participant: Ok! I’m a taxi driver here and I come to work with my Taxi.

Interviewer: How would you describe this area to others? Is this road busy?

- Participant: Here is Ofankor Barrier and very busy place. In the roundabout when you come here our taxi rank is Adom Korle Taxi Rank, so that’s it.

Interviewer: How big of a problem do you think accidents are here?

- Participant: ooh! Accidents don’t frequently happen here.

Interviewer: Is it fatal?

- Participant: NO, But when it rains, this place becomes flooded and this brings about huge road traffic over here. That is the problem.

Interviewer: What do you think causes accidents here? Road conditions (such as potholes, lack of sidewalks), abandoned/broken down vehicles, over speeding, wrong overtaking, traffic.

- Participant: And we don’t have pedestrian walkway over here and our road is not all that good. So mostly that is what causes accident here.

Interviewer: What do you think decreases the risk of an accident here?

- Participant: Normally if we (talking as a driver) exercise patience and understand each other as a driver that’s what will minimize accidents. But if misunderstanding sets in mostly that is what causes accident here. Over here mostly the accident doesn’t often happen like that but once in a while then one happens. Also, the motor riders when they wear their helmet, it reduces the risk of an accident. Have you seen the roundabout over there when a car is coming from the direction of the filling station and the other too is coming from Kumasi Road discerning towards the roundabout without patience and understanding from the drivers then accident will occur. That is where accident normally occur.

Interviewer: Does the accident normally affect children or hawkers?

- Participant: Ooh No! the accident is between vehicles.

Interviewer: Sometimes they say seeing is believing. Have you witnessed an accident scene before?

- Participant: Yes

Interviewer: Can you share with me? Your own or someone else you know?

- Participant: Ooh yeah, just last week a Benz and saloon car got crashed. The Benz was from the Kumasi Road towards the roundabout while the other saloon car too was from the other side of the roundabout. Even the Benz saloon tried to swerve the other saloon car but unfortunately due to impatience, the other saloon could not stop for the Benz to pass then the rear bumper of the Benz hit the front bumper of the saloon car to its removal. The Benz car did not even stop and then drove away.

Interviewer: On your own have experience any accident before?

- Participant: As a driver yes, but it did not happen here. It happens at East Lagon Ability Square. Last two years, I was Uber driver.

Interviewer: Can you tell me of a story about a child getting in an accident on the roads, if you have one?

- Participant: NO, I haven’t witnessed any accident scene involving a child

Interviewer: Now, let’s talk now about the police and their role. What do you think about the police’s enforcement of laws now? For example, over speed, motorcycle helmets, unlicensed driving, broken vehicles. Do you think this affects crashes?

- Participant: Yeah, normally if you are a driver, and you say you want to drive then you should own a license. As a qualified driver you have to own a license. If you don’t have license, it means you are unqualify driver. If you drive without a driving license and something happens you should be arrested and deal with you by law. And at the same time when driving a car, you should have your safety like safety belt and if you are a motor rider you should ride with a helmet. And on broken vehicles, for instance, if I’m driving a car and there is a broken-down track ahead of me in a curve. And I don’t know that there is a broken-down track in a curve without a warning triangle or sign. At least by all means there will be an accident.

Interviewer: So, what should the police do about enforcing the law.

- Participant: The police should check that if you are a driver, you should own a driving license. The police should also check the motor riders on their helmet usage. Again, the police should check each vehicle on the road about their safety equipment like warning triangle, red cross box etc, before allowing the vehicle to pass.

Interviewer: Once an accident does happen, what do you think causes people to die or get injured. Example the condition of the vehicle (trotro), no seat belt, no air bag, seat close to each other. Does this contribute to severe injury or death.

- Participant: that one too is true because the cars we use in our country though am a driver especially because the cars we use in our country is not all that good. Because for cars, if you are onboard your safety should be guaranteed. There should never be case where the seat of a car be closer to each other. No seat belt on and other safety fitting are all lost, is not fine. Some of the cars that we use in the country is evidence on its own, some of the car is too old and must be eliminated from the system but still found in the system. Do you understand, something like that, when the car becomes too old not all the part is correct on the car. So, when there happened to be an accident may be seat belt is spoilt or not available, airbag faulty and the rest of protective items are faulty. When there is an accident since all those things that protect the passengers are not available the accident will be fatal. Therefore, not all accident should come with serious injury or death. So, I think some of the old cars which are too old must be remove from our system. This will help reduce accident on our road. In Ghana some of the cars that we use here are not helpful to us at all.

Interviewer: Generally, which people typically get injured or die in an accident? For example, pedestrians, children, motorcyclists, bicyclists, hawkers, those without a helmet, those who do not use seat belts.

- Participant: Mm, its mostly the children, old women like our grandmothers and grandfathers because when the car is about to get accident they can’t run or they can’t run from the accident scene. so, someone like that when the car is coming, they don’t have any place to go. So, they will stand there. Like a child, when walking on the street is unmindful of himself, or something like that, he may be going to school, or something like that so, when accident happens, he who doesn’t care about thing end up being the one to get injured or die.

Interviewer: What about the environment such as the roads does it makes it more likely for a severe injury or death? For example, abandoned/broken down vehicles on the road, lack of sidewalks, potholes, traffic volume on roads, what about the condition of the road

- Participant: The road is good just that our traffic light and road signs is not all that good on our road. Also, about the potholes especially, sometimes when driving on the high way, whiles driving on, on aware you happen to meet a pothole. This causes accident especially in the night. So, we have to look at that aspect too in other to reduce accident.

Interviewer: what about broken down vehicle on the road

- Participant: on that issue, before man and God. If the car breaks down on the road without a warning sign that one too can cause accident because as we are going I don’t ahead of me that there is a broken-down car because as a driver if a car breaks down, we put a triangle on the car for others to know that the car is faulty or put on your hazard. As am coming I did not see any of those things, hazard too you didn’t on it. In such case I may think that the car is working so because I don’t know then eventually there is a crash. It then become a fatal accident. Therefore, the police have to watch out on those things.

Interviewer: What can be done to reduce the number of severe injuries and deaths here?

- Participant: We need traffic light to control traffic here and to reduce accident on the road.

Interviewer: The police should see to it that may be if there is a break-down vehicle on the road and the driver don’t have any warning sign.

- Participant: The people around should help push the car to proper place or the police should get a towing car to tow the car off the road. Or our traffic light and towing car should work 24/7 every day. The drivers should be careful on over speeding and the police should be checking the vehicle on the necessary safety items on the vehicle. If everything should be correct that will reduce accident. Our road too we should fill the potholes to avoid road accident. Moreover, we have to enforce road safety laws so that pedestrians and driver will be vigilant on the road and when driving too. Also, the police men should do their work well while on the road especially old care should remove from the road.

Interviewer: When people get into an accident, or get hurt, what happens? For example, do people call the police? Do people come help? Does an ambulance come? Tell me about what happens. Do the people call the police?

- Participant: Yes, It’s the people around the scene that call the ambulance and the police.

Interviewer: Does an ambulance come?

- Participant: Though, no big accident has ever happened here that will demand us to call the ambulance. So normally when accident occurs like that, maybe it is one among us who will pick his car put on the hazard light on to convey the injured person to nearby hospital. Before maybe they call the police to come.

Interviewer: Once again when you call the police do they come?

- Participant: Yes, they come.

Interviewer: When you call an ambulance, do they come?

- Participant: No, they don’t. I think there is no ambulance in this area. I don’t even know if there is any, because I haven’t seen ambulance after accident before.

Interviewer: OK, Just as you said you haven’t seen ambulance here or the police you have to walk to them so if you had the power, what would you do to improve care after an accident? For example, increasing number of ambulances, training people around in first aid.

- Participant: The work of the police is to protect us. So, if they have failed to do that. If I get power, I will make sure that they do what is expected of them. Also, I will make the police available every day on our road so that they will protect and enforce the road safety laws. Again, I will increase the number of ambulances over here. At least one ambulance will be station here, because when accident happens, it’s become an emergency so at once we have to take that person to hospital. Because if I say we should start first aid if care is not taken, we don’t know what will happen next, because when accident happens that person blood becomes hot and behavior changes. Which intern makes the person becomes high, which is difficult to handle. So, I will increase the number of ambulances so that at once when accident happens, quickly then we take them to hospital.

Interviewer: In your opinion, is accidents a problem in Ghana?

- Participant: Yes indeed, it’s a problem, a lot of accident are happening in the country and it’s not good at all.

Interviewer: Does the government consider your views when they make decisions on road safety?

- Participant: Normally I don’t know whether he listen or not, I can’t go and tell him to do this or that.

Interviewer: What is the government currently doing to reduce accidents? For example, speed bumps, law enforcement by police, pedestrian bridges, education campaigns Have you heard of those? Have you seen those?

- Participant: Yes, I’ve heard and seen some but I think they should build more. Especially at Agya Herbal a lot of people(pedestrians) crosses the road which causes more traffic jam.

Interviewer: So, at Agya Herbal does car knock people (pedestrians) over there?

- Participant: Yes,

Interviewer: children or older people?

- Participant: Older people

Interviewer: How old are they?

- Participant: I don’t know

Interviewer: We know other countries use enforcement cameras, where people get a fine immediately if they speed or run a red light – do you think we can do such a thing in Ghana?

- Participant: Yes

Interviewer: Why?

- Participant: This is because it will help arrest hit and run drivers and also help check over speeding and at the same time reduce road accident.

Interviewer: What mark will you give the government on a scale of 1-10 with 10 being the best?

- Participant: seven

Interviewer: Why that mark?

- Participant: Because I’ve seen some cameras around.

Interviewer: Finally, our last question for you is: If you had the power, what would you do to reduce accidents, injuries, and deaths on the roads nationally? Example the hawkers selling by the road side, motorcyclist who ride without helmet, children crossing the road and may be knock by cars. Tell me what would you do.

- Participant: I will construct separate pedestrians walk way for there to and from and Motorcyclist will have their way separate from the road. That is what I will do to reduce road accident.

Interviewer: Thank you Do you have anything to say in case we left something out.

- Participant: No thanks
