# Supplementary material for: “We are pleading for the government to do more”: Road user perspectives on the magnitude, contributing factors, and potential solutions to road traffic injuries and deaths in Ghana
Source: PLoS One. 2024 May 24;19(5):e0300458. doi: 10.1371/journal.pone.0300458 (PMC11125548; doi:10.1371/journal.pone.0300458)
Supplement: S2 File — (ZIP) [file pone.0300458.s002.zip › Transcripts to share/Participant_107_vulnerable.docx]

**Participant Number: 107**

**Language: Twi**

**Type of hot spot: Urban**

**Sex: Female**

**Road user type: Motorcycle**

Interviewer: How do you get to work? Do you use this road often? For example walking, public transport (trotros), motorcycles, cars, taxis, trucks, riding a bike, tricycles (i.e., pragya)

- Participant: I come to with motorcycle.

Interviewer: How would you describe this area to others? Is this road busy?

- Participant: Over Here is Ofankor barrier and is a busy road.

Interviewer: We have heard that accident is rampant here. Is it true?

- Participant: Yes, it is true even some has been happening before us to see.

Interviewer: Therefore, is accident a problem here?

- Participant: Yes

Interviewer: Then how big is it?

- Participant: Oh, it’s a problem, off late accident has become a problem which is difficult to solve. Not long ago, it’s about three weeks now we witness a fatal accident over here of which many lifes were lost. Every accident that occurs here are fatal and scarry.

Interviewer: What do you think causes accidents here? Is it the road conditions such as potholes, lack of sidewalks, abandoned/broken down vehicles, over speeding, wrong overtaking, traffic jam or what? Tell us.

- Participant: Of all that you said. The foremost is broken down vehicle. You know in Ghana when a car broke down on a road it takes a long time before it is removed or tawed from the road. When it delayed sometimes it can cause accident. Secondly, over speeding and overtaking is also among the causes of accident in the country. Some of the drivers don’t have patient when driving. Over speeding in the sense that some of the drivers want to reach their destination as fast as possible so that they can come for another passengers or load there by speeding abnormally so as to come for another load. Thirdly, Is the fault of our road. Some part of our road is not good at all for this reason drivers needs to be patient when driving on those roads in other to avoid accident. That is the three reasons I know causes accident here.

Interviewer: What do you think decreases the risk of an accident?

- Participant: First of all, is the road which they have started constructing it for us. We will have peace of mind if indeed they finished with the construction of the road. The road is part of the reason why accident occur here.

Interviewer: Are there some people who are more likely to get into an accident (for example: children, hawkers)?

- Participant: ooh! First off, all, it disturbs the hawker or traders here. that I have witness with my eyes are about the hawkers and the old men and women.

Interviewer: What about children?

- Participant: ooh! I don’t often see children injured in an accident. All the incident I have witness is about adult who often get injured.

Interviewer: what about hawker

- Participant: Ooh! In the case of hawkers, a lot of the accident has been involving them but no death or fatal injury recorded so far. Only minor injuries.

Interviewer: Have you witness or heard of any accident case in this area before?

- Participant: Yes, I myself have witness an accident scene with my naked eyes here before. I myself have seen a lot but not to say heard of it.

Interviewer: Ok! then tell me.

- Participant: What I saw with my eyes was about one tipper track driver who was discerning from the direction of the filling station. Which he eventually fails it brake and then overturn on to the ground spilling its load over the road. Even that the driver did not get any injury but was shaken. At the same time, another kia truck coming from the top there, fully loaded with alcoholic beverages. And then the track fail it brake and then run pass under the overhead bridge. Every one runaway and eventually got it way to stops here (under the overhead) where the woman is selling pineapple. But no one was injured.

Interviewer: Can you tell me of a story about a child getting in an accident on the roads, if you have one?

- Participant: No, I don’t have any.

Interviewer: Now, let’s talk now about the police and their role. What do you think about the police’s enforcement of laws now? For example, over speed, motorcycle helmets, unlicensed driving, broken vehicles. Do you think this affects crashes?

- Participant: Thank you, my brother. Yes, it affects crashes. The police have been doing their very best on this note to protect us. I don’t care if I’m a dispatch rider. Ghanaians, we are the stubborn ones be it drivers, dispatch rider, hawker by the road side. Once again Ghanaians we are the stubborn ones. We don’t follow orders. The police do their best to protect us.

Interviewer: Since we are stubborn. If you had the power, what would you do to change the situation here?

- Participant: If I have that power, I would move all the hawkers by the road side to a very safe place allocated to them. I will also maintain all the road with pot holes to it proper shape. And over speeding driver too I will put proper mechanism in place to check over speeding.

Interviewer: Once an accident does happen, what do you think causes people to die or get hurt, compared to just getting into a crash without getting hurt? For example, what about the condition of the vehicle or trotro makes it more likely for a severe injury or death? Like seat belts not working in cars/trotros, cars being old and not having air bags, position of seats, crowding

- Participant: Oh! No, that’s not the case, the above mention is not the cause of injuries and death on our roads. But on my opinion, the most cause of injuries and death on our roads are as a result of bad roads that causes accident to the death of people and injuries. Over speeding is among the cause of the accident, the reason been that if you are moving slowly and somebody is crossing, you will definitely see that person to be able to avoid any knock down. But some drivers don’t have patience, because of their in patience that causes most accident.

Interviewer: Like the car passing by and the passengers did not wear any seat belt or no seat belt in the car and there is a crash what do you think will happen.

- Participant: Oh! Most people will die and some too will be injured. So, what needs to be done is for every vehicle the passengers have to wear their seat belt. That is what will help us.

Interviewer: Which people typically get injured or die in an accident? For example, pedestrians, children, motorcyclists, bicyclists, hawkers, those without a helmet and those who do not use seat belts

- Participant: Ooh! It’s the hawkers and the pedestrians who crosses the road get injured than those in the car.

Interviewer: What about the environment such as the roads does it makes it more likely for a severe injury or death? For example, abandoned/broken down vehicles on the road, lack of sidewalks, potholes, traffic volume on roads.

- Participant: That’s true just as I have already said, broken down vehicles causes accident on the road especially on the high way. When a car breaks down on the road, it’s abandoned for say a day or two before it will be towed from the road. Sometimes it is even extent to a week or more before it will be towed. But if after break down the car is immediately towed from the road most accident would have been avoided.

Interviewer: What can be done to reduce the number of severe injuries and deaths here?

- Participant: The government should construct and maintain the road for us so as to avoid road accident. When the road becomes good there will be no accident, no injury nor death.

Interviewer: When people get into an accident, or get hurt, what happens? For example, do people call the police? Do people come help? Does an ambulance come? Tell me about what happens.

- Participant: Yes, they come.

Interviewer: Ok who calls the police nor they hear it and come?

- Participant: It is not the police that hear and come to the scene. At least what we see here is that we are closer to the police station. So, if anything happens, we are able to run fast to the station to call them.

Interviewer: When you call an ambulance, do they come?

- Participant: Please ambulance don’t come.

Interviewer: If you had the power, what would you do to improve care after an accident? For example, increasing number of ambulances, training people around in first aid.

- Participant: First of all, I will increase the number of ambulance and employed more drivers. If I get power, I have to be on my toes at least if something happens and you are been call you have to come fast. If you don’t come there’s law attach to it, you’ll be dealt with.

Interviewer: How much of a problem are accidents in Ghana?

- Participant: Hmmm in Ghana, what we have been witnessing with our eyes is many, at least about hundred people losses their life in only road accident every month.

Interviewer: Does the government consider your views when they make decisions on road safety?

- Participant: On television, they have been telecasting and broadcasting our message on radio so I believe the government see and hear it. But he chooses what to take and what to ignore.

Interviewer: What is the government currently doing to reduce accidents?

- Participant: The government that I have seen so far is doing a lot on road construction to reduce road accident. Example speed bump. Yeah, once he is constructing the road, he is definitely be doing all those things.

Interviewer: What about footbridges.

- Participant: Yeah, that one too he’s constructing some.

Interviewer: Ok! What about education campaigns like road safety department on street, radio and television training people on road safety issues. Have you heard of those?

- Participant: No please.

Have you seen them too?

- Participant: No please.

Interviewer: Why do you think the government chooses these? Are they considered better? Are they cheaper? Do you think the government considers cost when they pick what to do?

- Participant: Ooh! it’s not about being cheap but to the benefit of all. Also, it’s for us to safe from accident.

Interviewer: Where do ideas about road safety come from? Do you think the government looks to other countries? Or at research?

- Participant: Ooh no, it’s not from anywhere but from our own country.

Interviewer: We know other countries use enforcement cameras, where people get a fine immediately if they speed or run a red light – do you think we can do such a thing in Ghana?

- Participant: Yes

Interviewer: Why?

- Participant: This will really, help us. For instance, in the night where both of us are asleep it will help check over speeding drivers and during the day too especially the highway hit and run drivers can be captured and the law will deal with them. In fact, it’s my wish that this camera should be installed all over the country because it’s beneficial.

Interviewer: What mark will you give to the government on road safety and its department such as the Road Safety Department, Ghana High Way Authority and Ministry of Transport. On the scale of 1-10 with 10 being the best?

- Participant: Seven.

Interviewer: Why that mark?

- Participant: On my opinion I believe they have improved road safety issue though a lot needs to be done.

Interviewer: Finally, our last question for you is: If you had the power, what would you do to reduce accidents, injuries, and deaths on the roads nationally?What would you do for pedestrians?

- Participant: First of all, If I have the power the places where there is a footbridge, since some Ghanaians have proven to be stubborn, I will employ the service of the military men and police men to stand at a vantage point to enforce the road safety laws. They will make sure that pedestrians will not cross the road anyhow. Pedestrian shall also enforce to use their walk ways where necessary. Defaulter shall be jail for at least six months so that it will serve as a deterrence for others. By so doing accident will be minimized in the country.

Interviewer: What about motorcyclists?

- Participant: Motorcyclist too we don’t obey road traffic regulation thereby causing a lot of accident. Therefore, we will enforce the law on them. As I said defaulters should be jail for at least six months.

Interviewer: What about for children?

- Participant: For children they know nothing about the road safety laws. Therefore, I will add road safety laws to our educational curriculum so that it will enlightens their knowledge.

Interviewer: Is there anything else about crashes, injuries, or deaths on the roads that we haven’t discussed today that you would like to tell me?

- Participant: What I know is that if you are a driver and you are caught over speeding, you should be arrested and jail so that it will serve as a deterrence to others

Interviewer: Thank you for your time and participation in this important work.
