# Supplementary material for: “We are pleading for the government to do more”: Road user perspectives on the magnitude, contributing factors, and potential solutions to road traffic injuries and deaths in Ghana
Source: PLoS One. 2024 May 24;19(5):e0300458. doi: 10.1371/journal.pone.0300458 (PMC11125548; doi:10.1371/journal.pone.0300458)
Supplement: S2 File — (ZIP) [file pone.0300458.s002.zip › Transcripts to share/Participant_108_vulnerable.docx]

**Participant Number: 108**

**Language: Twi**

**Type of hot spot: Urban**

**Sex: F**

**Road user type: Pedestrian**

Interviewer: My sister, how do you get to work?

- Participant: I stay around this area so I walk to work every day.

Interviewer: How would you describe this area to others? Is this road busy?

- Participant: Here is Ofankor Barrier and it’s a busy road.

Interviewer: How big of a problem do you think accidents are here?

- Participant: Over here, because the police are careful here and the street-scent (is a type of community police service) works hand in hand with police, accident is not all that common here.

Interviewer: So, do you mean accident don’t occur here.

- Participant: Apart from neoplan area, over here no accident normally happens here.

Interviewer: where is neoplan area?

- Participant: I mean circle and its environment.

Interviewer: So, ever since you came here haven’t you heard of any accident before?

- Participant: Participant: I was a child when I heard of such.

Interviewer: How old were you then?

- Participant: Thirteen

Interviewer: Then tell us what happened.

- Participant: Back then it was a car crushing to another car. But no death or serious injury. I think that is not an accident.

Interviewer: Ah! All is part of an accidents; it is the reason why we are here. Therefore, what do you think causes accidents here? Road conditions (such as potholes, lack of sidewalks), abandoned/broken down vehicles, over speeding, wrong overtaking, traffic.

- Participant: Sometimes impatient from drivers causes accident here. Again, lack of traffic light here also contributes to accident and cars knocking down people.

Interviewer: What do you think decreases the risk of an accident?

- Participant: Credit to the police men and the street-scent who are always by the road side enforcing road safety laws. Sometimes even as late as 10pm they will still be around.

Interviewer: Are there some people who are more likely to get into an accident (for example: children, hawkers)?

- Participant: No, I have not witnessed any accident scene at this place before involving children and hawkers.

Interviewer: Sometimes personal stories can make road traffic problems more real. However, we know this can be sensitive. If you feel comfortable, can you share a story from an accident with me? Your own or someone else you know?

- Participant: Oh! I have been hearing some on radio and television every day.

Interviewer: were children part of the accident?

- Participant: Yes, at times, children are involved.

Interviewer: How old was those children?

- Participant: Some are newly born babies. I even heard some in Kumasi, many people got injured, some were beheaded. In that incident one of my friends were among those casualties. Sorry

Interviewer: Then tell us how it happens

- Participant: Once a friend of mine and her daughter were involved in an accident at Sofo-line a suburb of Kumasi where by an articulated track flat body run over them to their death on the sport. The track driver was under the influence of alcohol according to the police investigation and eye witnesses report. But when ask he said brake failure cause that.

Interviewer: How old was the child?

- Participant: Hmmm, a toddler.

Interviewer: Now, let’s talk now about the police and their role.

- Participant: Just as I said we need a traffic light to regulate the traffic here for us.

Interviewer: What do you think about the police’s enforcement of laws now? For example, speed, motorcycle helmets, unlicensed driving, broken down vehicles Do you think this affects crashes?

- Participant: Yes, it contributes to crashes. Especially, the motorcyclist if in case they are involve in an accident it will protect them from head injury. Also, the seat belt will protect passengers from fatal injury and sometimes even head injury too. So, the police should enforce these laws so that injuries in an accident will be reduce.

Interviewer: If you had the power, what would you do to change the situation here?

- Participant: I will construct traffic light here so that it will regulate traffic over here.

Interviewer: Once an accident does happen, what do you think causes people to die or get hurt. For example, the condition of the vehicle or trotro makes it more likely for a severe injury or death? Like seat belts not working in cars/trotros, cars being old and not having air bags, position of seats, crowding.Tell us something.

- Participant: Mmm! It contributes to accident because some of the cars are very weak. Sometimes their engine is not all that good. Therefore, if the police will help remove those old cars from the road it will help. Once I onboard a car from Ofankor, here to Circle, whiles onboard the seat knot removed from the seat and I nearly got injured. Also, for some their engines are too weak and will be polluting the environment. So, if all these cars are still in Accra the government has to do something about it. Remove them and replace with new ones. So that it will protect our life from further accident or death. All these are among the reasons for the numerous deaths in the country.

Interviewer: Generally, which people typically get injured or die in an accident? For example, pedestrians, children, motorcyclists, bicyclists, hawkers those without a helmet, those who do not use seat belts

- Participant: Over here, may be its those crossing the road. Since we the hawkers has been ask to move backwards to ensure our safety, any accident that will happened here we will be save from it.

Interviewer: What about the environment (such as the roads) makes it more likely for a severe injury or death? For example, abandoned/broken down vehicles on the road, lack of sidewalks, potholes, traffic volume on roads.

- Participant: Yes, especially if a car breaks down on the road, the driver may say I am going to call mechanic to come and fix my car. Whiles waiting, instead of the driver to put down warning triangle to alert the incoming vehicle so that accident can be avoided. They fail to do so. In such cases accident may occur.

Interviewer: What can be done to reduce the number of severe injuries and deaths here?

- Participant: Over here due to the vigilance of the police and how the road is there is no severe injuries and death in an accident her.

Interviewer: When people get into an accident, or get hurt, what happens? For example, do people call the police? Do people come help? Does an ambulance come? Tell me about what happens.

- Participant: Because it’s not serious we don’t call neither the police nor ambulance.

Interviewer: So, you when car knock someone the person just gets up and go. Is that what you mean?

- Participant: No not that but the accident normally happens between two cars. Like two private cars one following the other in a traffic and one will hit the others bumper. in such case they don’t involve the police but rather settle the matter themselves and move on.

Interviewer: If you had the power, what would you do to improve care after an accident? For example, increasing number of ambulances, training people around in first aid.

- Participant: Ah! well, I will educate the general public on road safety and construct traffic light here.

Interviewer: Over the past ten years, our country has recorded seventy-eight thousand casualties and fourteen thousand deaths in an accident. For this reason, there is the need for us find out the cause of these death. Is accident problem in Ghana?

- Participant: Yes, it’s a problem

Interviewer: Then how much is accident affection the country.

- Participant: Accident is affecting the country in so many ways. Many relatives and love ones have been lost through accident. Today in Ghana there is fear and panic especially if a relative tells you that am traveling. Then the entire family begins to put the destiny of that relatives in the hands of God for fear of not returning to them. That is the extent to which accident is affecting the country.

Interviewer: Does the government consider your views when they make decisions on road safety?

- Participant: Please I don’t know, I don’t actually know. By all means he listen to it.

Interviewer: What is the government currently doing to reduce accidents? For example, speed bumps, enforcement by police, pedestrian bridges, education campaigns Have you heard of those?

- Participant: Yes, I have heard of them on radio.

Interviewer: Have you seen those?

- Participant: Yes, but on television. I saw the government constructing something like footbridge, zebra crossing, speed bump and the rest.

Interviewer: Why do you think the government chooses these? For example, speed bumps, law enforcement by police, pedestrian bridges, education campaigns. Are they considered better?

- Participant: Please I don’t know.

Interviewer: Are they cheaper? Do you think the government considers cost when they pick what to do?

- Participant: Please I have no idea.

Interviewer: Where do the ideas about road safety come from? Do you think the government looks to other countries?

- Participant: Yes, since he has been traveling, he learns from them.

Interviewer: Or at research?

- Participant: Mmm, may be from this research the government can get some ideas from it.

Interviewer: We know other countries use enforcement cameras, where people get a fine immediately if they speed or run a red light – do you think we can do such a thing in Ghana?

- Participant: Yes, it will help us a lot.

Interviewer: Why?

- Participant: It will also check over speeding vehicle.

Interviewer: What mark will you give the government on a scale of 1-10 with 10 being the best?

- Participant: Five.

Interviewer: Why that mark?

- Participant: Because they are improving.

Interviewer: Finally, our last question for you is, if you had the power, what would you do to reduce accidents, injuries, and deaths on the roads nationally? What would you do for pedestrians?

- Participant: I will make the police to stand by any road side to enforce road safety laws. And also educate the general public on road safety.

Interviewer: What about motorcyclists?

- Participant: I will pass a law that moto cyclist and bicycle riders should wear their helmet. The case of bicycle riders is fairly good but for the moto rider they are those who suppose to wear their helmet because of their speed they can get accident at any time. So, if you wear helmet and may be accident happens it can protect your head.

Interviewer: What about for children?

- Participant: Since children are not mature people, I will pass a law with standard of car children must onboard and the strict on parent to check the type of car their children must onboard. so that accident involving children will be reduced. I will also educate children on road safety especially how to cross the road.

Interviewer: Is there anything else about crashes, injuries, or deaths on the roads that we haven’t discussed today that you would like to tell me?

- Participant: No please.

Interviewer: Thank you for your time and participation in this important work.
