# Supplementary material for: “We are pleading for the government to do more”: Road user perspectives on the magnitude, contributing factors, and potential solutions to road traffic injuries and deaths in Ghana
Source: PLoS One. 2024 May 24;19(5):e0300458. doi: 10.1371/journal.pone.0300458 (PMC11125548; doi:10.1371/journal.pone.0300458)
Supplement: S2 File — (ZIP) [file pone.0300458.s002.zip › Transcripts to share/Participant_109_non_vulnerable.docx]

**Participant Number: 109**

**Language: Twi**

**Type of hot spot: Urban**

**Sex: Male**

**Road user type: Truck driver**

Interviewer: Do you use this road often?

- Participant: Yes please.

Interviewer: What do you do here?

- Participant: I am driver.

Interviewer: How do you get to work? For example, walking, public transport (trotros), motorcycles, cars, taxis, trucks, riding a bike, tricycles (i.e. pragya).

- Participant: I drive my truck to work.

Interviewer: How would you describe this area to others?

- Participant: Here is Ofankor Barrier, the road to Agya Herbal.

Interviewer: Is this road busy

- Participant: Yes, very busy

Interviewer: How big of a problem do you think accidents are here?

- Participant: Issues of accident is a big problem here. Sometimes due to the road nature the drivers descend with a though speed. When they reach at the entrance of the overhead deciding to branch downwards or go straight becomes a problem. This sometimes causes accident here. Not long ago, say about two weeks now. Some happened just at the entrance of the overhead and two people lost their life.

Interviewer: What do you think causes accidents here? Is it the road conditions such as potholes, lack of sidewalks, abandoned/broken down vehicles, over speeding, wrong overtaking, traffic.

- Participant: Most of the accident that occurs here are the fault of the drivers. What happens is that they mostly fall asleep and then lose control over the steering wheel. Also, some too fails brake and in the end causes accident. This is what I know causes accident here.

Interviewer: What do you think decreases the risk of an accident?

- Participant: What I think will decrease the risk of accident here is the construction of the new six dual lane road, when done I think will solve the issues of accident here.

Interviewer: Are there some people who are more likely to get into an accident (for example: children, hawkers)? Which age of children?

- Participant: Over here I have not seen any accident which involve any child. Mostly it’s those in the cars that get injured more, grownups and the aged people.

Interviewer: Sometimes personal stories can make road traffic problems more real. However, we know this can be sensitive. If you feel comfortable, can you share a story from an accident with me? Your own or someone else you know?

- Participant: Just as I said, about two weeks ago an articulated track crashed just at the entrance of the overhead. The driver and the community police directing traffic died on the sport. Again, about a month ago a car knocks a pedestrian crossing at the entrance of the overhead. He died on the sport. This accident occurred at the other side of the road from Agya Herbal towards Ofankor Barrier.

Interviewer: Can you tell me of a story about a child getting in an accident on the roads, if you have one?

- Participant: I have not heard of any accident involving children at this place before.

Interviewer: Now, let’s talk about the police and their role. What do you think about the police’s enforcement of laws now? For example, speed, motorcycle helmets, unlicensed driving, broken vehicles. Do you think this affects crashes?

- Participant: First of all, over speeding is among the causes of road accident especially from Agya herbal to this Ofankor Barrier. Most of the accident cases are cause by over speeding. So, if the police enforce the laws, it will minimize accident here. Secondly, the police should do their best to enforce the law here. During the day, they enforce the law very well and accident minimizes here. But in the night where they are gone is where most of the accident occurs. Also, at night when a car breaks down on the road without a warning sign and no reflectors. An incoming vehicle driver might not even see it. Not even when he has thrown his high light on, before he could realize there is a crashed. Therefore, breakdown vehicle sometimes causes road accident.

Interviewer: If you had the power, what would you do to change the situation here?

- Participant: If I have the power, I will construct a barrier at the Don Tei up there. So, that the police on duty will check any vehicle before it gets here. By so doing the speed of that vehicle will be limited before it gets here. Also, if a driver is sleeping, he will wake up. Even if you are speeding, you can’t speed much before you reached here. This will control the accident here.

Interviewer: Once an accident does happen, What do you think causes people to die or get hurt, compared to just getting into a crash without getting hurt? For example, what about the condition of the vehicle or trotro makes it more likely for a severe injury or death?Like seat belts not working in cars/trotros, cars being old and not having air bags, position of seats, crowding

- Participant: Accidents can occur in any vehicle, regardless of its age or condition, as all vehicles are man-made machines that are subject to failure even with regular maintenance.

Interviewer: Generally, which people typically to get injured or die in an accident? For example, pedestrians, children, motorcyclists, bicyclists, hawkers, those without a helmet, those who do not use seat belts

- Participant: Base on what I have witnessed it’s mostly the hawkers and the pedestrians.

Interviewer: What about the environment (such as the roads) makes it more likely for a severe injury or death? For example, abandoned/broken down vehicles on the road, lack of sidewalks, potholes, traffic volume on roads.

- Participant: The condition of the road too is a factor especially when you are driving from Don Tei to this place (Ofankor Barrier), the road is dual but when you reach here (Ofankor Barrier) it becomes four. Over there instead of them to open the road well so that each driver will know where to drive to, it not like that. So, when any strange or unfamiliar driver get there, they become confuse as to where to pass. This also can contribute to accident.

Interviewer: What can be done to reduce the number of severe injuries and deaths here?

- Participant: If we construct police barrier at Don Tei, it will check over speeding, drivers who sleep while driving and so on. By so doing it will reduce accident in the area.

Interviewer: When people get into an accident, or get hurt, what happens? For example, do people call the police? Tell me about what happens.

- Participant: Yes, people call the police. Before I continue let’s applaud the police men in this area. The accident which occurred the last time in this place, the police got there even before us.

Interviewer: Do people come to help?

- Participant: Yes, people assist in saving lives.

Interviewer: When you call an ambulance, do they come?

- Participant: Yes, they come.

Interviewer: Do people call them or they come by them self.

- Participant: People call them. As for me I have not called them before but people do. Sometimes when we get there, in seconds you could hear their siren

Interviewer: How long would an ambulance take to arrive?

- Participant: I haven’t called some before but immediately after accident before we get there the police will be there too. within a second an ambulance will follow. Sometime even fire service also come.

Interviewer: Who gets an ambulance and who doesn’t? For example, does it depend on if you are in an urban or rural area? Or the conditions of the road? Or if it’s a major road and it causes congestion?

- Participant: Ooh No, they don’t do that. But they don’t pick dead bodies.

Interviewer: If you had the power, what would you do to improve care after an accident? For example, increasing number of ambulances

- Participant: ooh! I will add another ambulance to the constituency. Already we have one here.

Interviewer: What about training people around in first aid.

- Participant: If I have power, I will make education on first aid becomes more especially in a place where there is no network reception so that if accident occur. Before the arrival of ambulance and the police the people in the area might have save some lives. Also, if any one gets involve in an accident and the person is not dead, they can save that person’s life. For this reason, I will make education to be more.

Interviewer: In your opinion, how much of a problem are accidents in Ghana?

- Participant: Hmmm it’s a very BIG problem.

Interviewer: Does the government consider your views when they make decisions on road safety?

- Participant: They don’t listen to us. If they were to be a listening government, they would have listened to our voice. Ever since they constructed Nsawam to Apagya dual road, accident on that stretch have stop. Therefore, if they were to be a listening government Kumasi to Accra Road which we say should be made dual because it’s the road with the highest accident record, they would have listened to us. If not, accident will not reduce, so far as it remains single road. Therefore, if indeed, they say they are listening government they should do it for us.

Interviewer: What is the government currently doing to reduce accidents? For example, speed bumps, pedestrian bridges, police enforcing the law, education campaigns. Have you heard of those? Have you seen those?

- Participant: The government don’t really care about us on road accident issues. The moment they get that post all that they care is about their pocket and their position. If they really care about us, they would have made most of the road dual. They have not made up their mind to help us. Also, apart from the police who are always enforcing the law the rest are doing nothing. As for them they will be on road all the time.

Interviewer: Why do you think the government chooses these? Example speed bumps, pedestrian bridges, police enforcing the law, education campaigns. Are they considered better? Are they cheaper? Do you think the government considers cost when they pick what to do?

- [No answer]

Interviewer: Where do ideas about road safety come from?

Interviewer: Do you think the government looks to other countries? Or at research?Participant:

- Participant: They sometimes look to other country and also from the research you are doing. But I would also like to add this. The footbridge has been constructed for pedestrian use at Pokuase but you always find them crossing the road and not using it. I would suggest that the government deploys soldiers on that stretch to discipline those who don’t use the footbridge. I believe this will help reduce accidents.

Interviewer: We know other countries use enforcement cameras, where people get a fine immediately if they speed or run a red light – do you think we can do such a thing in Ghana?

- Participant: Yes, it will help.

Interviewer: Why?

- Participant: This is because it will help track over speeding drivers.

Interviewer: What mark will you give the government on a scale of 1-10 with 10 being the best?

Participant: I will give them one

Interviewer: Why that mark?

- Participant: This because they have done little on road safety. The reason for this is that every year figures on accident keeps on increasing example, if this year we recorded 1000 accident cases next year will be maybe 1300 or 1500. Like that no decrement. They are those who come out with those figures. To be frank the government seems not to care about it. Nothing serious have been done about it.

Interviewer: Finally, our last question for you is, if you had the power, what would you do to reduce accidents, injuries, and deaths on the roads nationally? What would you do for pedestrians?

- Participant: Firstly, just as I already said any place that is unauthorize for people to cross but disobey to cross. I will make soldiers to stand there. If you are caught crossing from an unauthorize place they should lash your back with cane or they should make you clean gutters for one week. When you are caught they will find out where you live, early in the morning the police will come for you to go and clean the gutters. This will continue and monitored till one week before releasing you.

Interviewer: What about motorcyclists?

- Participant: For motor riders if you don’t have license, I will not allow you to ride motor. Because the boy uses it for stupid things. So, if you are caught riding without helmet, you too will do community service like cleaning of gutter. This will even make the city clean and clear all dirt from the city.

Interviewer: What about the children?

- Participant: The children who died on the road as a result of road accident. If it’s the drivers whose knowledge is low, I together with road safety department will give them education on road safety. Even if, the drivers will have to pack their car and go to school twice in every week as a refresher course it will help. Some drivers don’t even know the road signs. Like a sign telling driver to stop for people to pass they don’t know or at a point when he gets to roundabout for him to stop for others, they don’t have that knowledge. Majority of them don’t even know that here is one way. All this put together if I have that power, I will see to it that they will work with it.

Interviewer: Is there anything else about crashes, injuries, or deaths on the roads that we haven’t discussed today that you would like to tell me?

- Participant: Let me add this to it. On the issue of vehicles knocking down pedestrians especially where there is a footbridge like Pokuasi, Achimota and the rest. I have realized that Ghanaians we are too indiscipline for that reason, there should be military men in place to enforce the law in those areas. Defaulter has to be cane or force to do community service like cleaning the choke gutters and others. This will reduce death and injuries cause by knock down by cars. I would like to urge the police to ensure the proper enforcement of traffic laws on drivers, as sometimes they fail to fulfill their duties as law enforcement officers.

Interviewer: Thank you for your time and participation in this important work.
