# Supplementary material for: “We are pleading for the government to do more”: Road user perspectives on the magnitude, contributing factors, and potential solutions to road traffic injuries and deaths in Ghana
Source: PLoS One. 2024 May 24;19(5):e0300458. doi: 10.1371/journal.pone.0300458 (PMC11125548; doi:10.1371/journal.pone.0300458)
Supplement: S2 File — (ZIP) [file pone.0300458.s002.zip › Transcripts to share/Participant_110_non_vulnerable.docx]

**Participant Number: 110**

**Language: Twi**

**Type of hot spot: Urban**

**Sex: Male**

**Road user type: Commercial Driver**

Interviewer: How do you get usually to work? For example, walking, public transport (trotros), motorcycles, cars, taxis, trucks, riding a bike, tricycles (i.e., pragya)

- Participant: I am a dispatch rider so I use motor when coming to work

Interviewer: How would you describe this area to others? Is this road busy?

- Participant: Here is Ofankor Barrier and is a busy place.

Interviewer: How big of a problem do you think accidents are here?

- Participant: Accident is a problem to us.

Interviewer: What do you think causes accidents here? Road conditions (such as potholes, lack of sidewalks), abandoned/broken down vehicles, over speeding, wrong overtaking, traffic

- Participant: In short, when it rains this place become flooded. So am sure that is the cause of accident here.

Interviewer: What do you think decreases the risk of an accident?

- Participant: It is my wish that they should reconstruct the gutter here so that any time it rains there will be no flood. This when done will decrease accident. That is what I see it.

Interviewer: Are there some people who are more likely to get into an accident (for example: children, hawkers)?

- Participant: the children and the hawkers because when this place becomes flooded it create traffic jam. Sometimes because of the rain some people become afraid and began to panic so they hurriedly cross the road an eventually knockdown by car. Some even get injured as a result of that.

Interviewer: So those who get injured are children among?

- Participant: yes

Interviewer: what is their age?

- Participant: About to sixteen to seventeen.

Interviewer: Sometimes personal stories can make road traffic problems more real. However, we know this can be sensitive. If you feel comfortable, can you share a story from an accident with me? Your own or someone else you know?

- Participant: I frequently see some here. Some often happens at the top of the overheard. Motor accident often occurs under the overheard, at the crossing and over here too, cars often kill a lot of the okada riders (dispatch riders).

Interviewer: Can you tell me of a story about a child getting in an accident on the roads, if you have one?

- Participant: Please I don’t remember.

Interviewer: Now, let’s talk now about the police and their role. What do you think about the police’s enforcement of laws now? For example, speed, motorcycle helmets, unlicensed driving, broken vehicles Do you think this affects crashes?

- Participant: Ooh! the police are doing their work well. Over here if I say that the police are not doing their work well then am not speaking the truth. They even work very well at night. Sometime some of the drivers are very wicked. At times some drivers attempt to even knock a police man down. Sometimes drivers who drives without license out of fear and panic will crash into accident and worsen their case. Although I have praised the police. There’s still more to done on the field. Especially over speeding, unlicensed driving and checking of helmet. I believe if all is done accident will reduce. I wish the police should build a barrier here so that they will check overspending, those without driving license especially we the moto riders without helmet and other road safety measures.

Interviewer: If you had the power, what would you do to change the situation here?

- Participant: Please as for that one I can’t say anything about it.

Interviewer: Once an accident does happen, What do you think causes people to die or get hurt, compared to just getting into a crash without getting hurt? For example, what about the condition of the vehicle or trotro makes it more likely for a severe injury or death? Like seat belts not working in cars/trotros, cars being old and not having air bags, position of seats, crowding

- Participant: Sometimes the driver driving the car is intoxicated with alcohol whiles driving. Some too don’t have seatbelt, the passengers too are among the cause because some passengers will be yelling at the driver to speedup because they are in a hurry. Therefore, some of the accidents are the fault of the driver and the passengers.

Interviewer: Generally, which people typically get injured or die in an accident? For example, pedestrians, children, motorcyclists, bicyclists, hawkers, those without a helmet, those who do not use seat belts.

- Participant: What I have notice is the pedestrians, the passengers and the hawkers.

Interviewer: What about the environment (such as the roads) makes it more likely for a severe injury or death? For example, abandoned/broken down vehicles on the road, lack of sidewalks, potholes, traffic volume on roads.

- Participant: Over here the road is good but, there is small place that needs to be repatch. But if they should come to our aid by expanding the road it would have been better. Truth be told over here the road is not good.

Interviewer: What about abandoned/broken down vehicles on the road, lack of sidewalks, potholes, traffic volume on roads. Does it contribute accident?

- Participant: Answer as for me what I have notice is the traffic volume that causes accident here. There is no place for pedestrians to pass. In the evening it becomes difficult for people to cross, because when the traffic jams some of the car if you stop them to say maybe you want to cross, they will not stop top for people to cross the road. So, the least mistake that you commit you’ll be knock down. Because some of the drivers drive under the influence of alcohol.

Interviewer: What can be done to reduce the number of severe injuries and deaths here?

- Participant: Again, they should check the drivers and their cars some has faulty break, some don’t have license that bring police pressure on the driver for fear of been arrest. So the new trend is that for those drivers who don’t have license when they see the police they then dash them with a token for the police to over look things. So, I will plead with the police to put a stop to that and to do the bidding of the nation.

Interviewer: When people get into an accident, or get hurt, what happens? For example, do people call the police? Do people come help? Does an ambulance come? Tell me about what happens.

- Participant: Yes please. Also, it is the people who help the injured passengers.

Interviewer: When you call an ambulance, do they come?

- Participant: No please, they don’t.

Interviewer: If you had the power, what would you do to improve care after an accident? For example, increasing number of ambulances, training people around in first aid.

- Participant: I would pay attention to the Ambulance service and make sure they work or they attend to people when needed. I will also fix the road around this area and create a special road for the motor riders because we always see them standing in the middle of the road. Also with the pedestrian I will construct a walkway for them to reduce accidents.

Interviewer: In your opinion, how much of a problem are accidents in Ghana?

- Participant: Accident is a problem to the entire nation. Almost every day you hear of accident cases in the country. And just as I said early on, some of our roads are in bad condition. Also some of the drivers have too much to drink which causes accidents.

Interviewer: Does the government consider your views when they make decisions on road safety?

- Participant: What I know is that the government cannot listen to all the things that we say. Maybe some of the things that we say might be in the plans of the government but he has not done it yet.

Interviewer: What is the government currently doing to reduce accidents? For example, speed bumps, enforcement by police, pedestrian bridges, education campaigns. Have you heard of those?

- Participant: It seems government is doing a lot to reduce accidents like constructing new roads etc. But not all the roads have been fixed yet. For example with this road, we all don’t know why but there’s always a flood when it rains.I have not heard of any educational campaign on road safety.

Interviewer: Have you seen those?

- Participant: Yes, I have seen the police enforcing the law. I have also seen the construction of footbridge but not many.

Interviewer: Why do you think the government chooses these? For example, speed bumps, enforcement by police, pedestrian bridges, education campaigns. Are they considered better?

- Participant: Yes

Interviewer: Are they cheaper? Do you think the government considers cost when they pick what to do?

- Participant: No, I don’t think so.

Interviewer: Where do ideas about road safety come from? Do you think the government looks to other countries? Or at research?

- Participant: Yes, I think he travel to get that knowledge.

Interviewer: We know other countries use enforcement cameras, where people get a fine immediately if they speed or run a red light – do you think we can do such a thing in Ghana?

- Participant: I don’t know what a speed camera is but from the explanation you gave I believe when they bring some in the country it will help for its good idea.

Interviewer: Why?

- Participant: If they installed camera on the road, it will caution driver when to speed and when not to speed.

Interviewer: What mark will you give the government on a scale of 1-10 with 10 being the best?

- Participant: 10.

Interviewer: Why that mark?

- Participant: They have done well.

Interviewer: Finally, our last question for you is, if you had the power, what would you do to reduce accidents, injuries, and deaths on the roads nationally? What about motorcyclists?

- Participant: If I have power, I will make the okada riders to stop work for I have seen that its due to economic hardship that is making them do that. So, if I have power, I will create job for each of them. And for the accident too I will check the cause of those accident and make sure it’s solved so that everything to alright.

Interviewer: Is there anything else about crashes, injuries, or deaths on the roads that we haven’t discussed today that you would like to tell me?

- Participant: No thanks

Interviewer: Thank you for your time and participation in this important work.
