# Supplementary material for: “We are pleading for the government to do more”: Road user perspectives on the magnitude, contributing factors, and potential solutions to road traffic injuries and deaths in Ghana
Source: PLoS One. 2024 May 24;19(5):e0300458. doi: 10.1371/journal.pone.0300458 (PMC11125548; doi:10.1371/journal.pone.0300458)
Supplement: S2 File — (ZIP) [file pone.0300458.s002.zip › Transcripts to share/Participant_111_vulnerable.docx]

**Participant Number: 111**

**Language: Twi**

**Type of hot spot: Urban**

**Sex: Male**

**Road user type: Motorcyclist**

Interviewer: How do you get to work? For example walking, public transport (trotros), motorcycles, cars, taxis, trucks, riding a bike, tricycles (i.e., pragya)

- Participant: I use motorcycle

Interviewer: How would you describe this area to others as far as accident is concern? Is this accident common here?

- Participant: Yes, very common accident.

Interviewer: How big of a problem do you think accidents are here?

- Participant: Accident often occurs here. So, it’s a very big problem.

Interviewer: What do you think causes accidents here? Road conditions (such as potholes, lack of sidewalks), abandoned/broken down vehicles, over speeding, wrong overtaking, traffic.

- Participant: Over speeding. Over here the road is small, so if a driver is driving on though speed from Pokuasi and want to turn right and then descend downwards towards the filling station that is where the problem lies. Whiles on though speed he can’t descend easily like that and eventually loses control and will either hit some car or two tires, (a moto bike) or someone riding moto bike, you will hear ‘kpa sound’ then the rider is down. We have seen this several times. At times, a car will break down on the road for several days like two days, three days, four days even it sometimes extends beyond that. In some cases, it will remain there until a mechanic come to fix it. This in turn causes accident here. This, mostly happens on Fridays.

Interviewer: What do you think decreases the risk of an accident?

- Participant: That is what am saying, the road that is under construction when they finished accident will reduce. When they finished with construction the road will become four dual or six dual road.

Interviewer: Are there some people who are more likely to get into an accident (for example: children, hawkers)?

- Participant: Ever since I came here, I have witness only three people who has been knock down by cars while crossing the road.

Interviewer: were they children, hawkers or pedestrians?

- Participant: Mostly those in the car.

Interviewer: Are they children?

- Participant: No. An adult.

Interviewer: Sometimes personal stories can make road traffic problems more real. However, we know this can be sensitive. If you feel comfortable, can you share a story from an accident with me? Your own or someone else you know?

- Participant: What happened was that once a saloon car in the inner lane wanted to drive to the outer lane and descend downwards but I don’t know what happened all that I saw was a track hit the saloon and then another car too hit other like that. I think it’s due to the speed of those car that led to such accident. Always the cars are on high speed so little mistake then there is an accident.

Interviewer: Can you tell me of a story about a child getting in an accident on the roads, if you have one?

- Participant: Over here no, I haven’t seen some before.

Interviewer: Now, let’s talk now about the police and their role. What do you think about the police’s enforcement of laws now? For example, speed, motorcycle helmets, unlicensed driving, broken vehicles

- Participant: Any time there is an accident the police come around. The police do their work well. They check license and helmet too because they always arrest me on the issue of driving license and helmet. They always arrest me on those issues. They will fine me in addition because I use motorbike without a license and helmet. Hahahaa, (we both laugh). I have to speak the truth for they do their work well.

Interviewer: Do you think these affects crashes?

- Participant: You know accidents are caused by the mistakes of drivers or other road users. So I can’t really tell unless God.

Interviewer: If you had the power, what would you do to change the situation here?

- Participant: I will expand the road; I will set up police check point or barrier at Pokuasi to monitor drivers. I will also build road for moto cyclist and bicycle.

Interviewer: Once an accident does happen, what do you think causes people to die or get hurt, compared to just getting into a crash without getting hurt? For example, what about the condition of the vehicle or trotro makes it more likely for a severe injury or death? Like seat belts not working in cars/trotros, cars being old and not having air bags, position of seats, crowding

- Participant: This is true because some of the vehicles which has been passing here needs to be seized. Especially with some articulated track, their break is air and if they become old everything seizes to work properly. Some when they are descending down in such case a little obstruction then here come accident because for him, he doesn’t have a way of dodging. Again, sometimes those old tracks mostly fail break thereby causing fatal accident, injury and death.

Interviewer: Generally, which people typically to get injured or die in an accident? For example, pedestrians, children, motorcyclists, bicyclists, hawkers Those without a helmet, those who do not use seat belts.

- Participant: Those seated in the car. They I have seen that some get injured or die. But for hawkers no.

Interviewer: What about the environment (such as the roads) makes it more likely for a severe injury or death? For example, abandoned/broken down vehicles on the road, lack of sidewalks, potholes, traffic volume on roads.

- Participant: Yes, to begin with, the road its self is small over here and it needs to be expanded. This sometimes causes accident here. Secondary, I said this earlier, abandoned vehicle sometimes is the reason for some of the accident here. When the vehicle especially tracks break down here it’s abandoned for say three days, four days and even beyond. If abandoned, since the road is a high way and drivers drive with higher speed. Sometimes due to fatique some drivers crash to it. Lastly, as you can see, over here there is no side walk. This can make the life of pedestrians and moto riders very dangerous on the road. Also, the street light sometimes it can take four days before it’s powered on. This and other reasons make life difficult for pedestrians. Sometimes my heart even tremble for some moto riders’ pedestrians.

Interviewer: What can be done to reduce the number of severe injuries and deaths here?

- Participant: If we expand the road with side walk. And every vehicle which breakdown here is towed accident will reduce. If they build a barrier here to be checking the cars, And the street light when fixed accident cases will reduce. If we do that, I hope severe injury and dearth can be reduced.

Interviewer: When people get into an accident, or get hurt, what happens? For example, do people call the police? Do people come help? Does an ambulance come? Tell me about what happens.

- Participant: Any time there is an accident the police come from mile7 police station. I even know them. They come within 30 minutes.

Interviewer: When you call an ambulance, do they come?

- Participant: They come but sometimes when a car is passing by, we will then stop the car and convey the injured person to the hospital. Mostly, they come at dawn or in the night when an accident occur.

Interviewer: How long would an ambulance take to arrive?

Interviewer: Who gets an ambulance and who doesn’t? For example, does it depend on if you are in an urban or rural area? Or the conditions of the road? Or if it’s a major road and it causes congestion?

Interviewer: If you had the power, what would you do to improve care after an accident? For example, increasing number of ambulances, training people around in first aid

- Participant: I will increase the number of ambulances in the areas of hot sport. I will also train the general public on first aids and road safety.

Interviewer: In your opinion, how much of a problem are accidents in Ghana?

- Participant: Hmmm accident is a major problem here.

Interviewer: Does the government consider your views when they make decisions on road safety?

- Participant: I don’t think so.

Interviewer: What is the government currently doing to reduce accidents? For example, speed bumps, enforcement by police, pedestrian bridges, education campaigns

- Participant: I think the government is doing something but they can’t do everything.

Interviewer: Have you heard of those?

- Participant: I have heard of those except education campaigns.

Interviewer: Have you seen those?

- Participant: No

Interviewer: Why do you think the government chooses these? For example, speed bumps, enforcement by police, pedestrian bridges, education campaigns. Are they considered better?

- Participant: I don’t know.

Interviewer: Are they cheaper? Do you think the government considers cost when they pick what to do?

- Participant: I don’t know.

Interviewer: Where do ideas about road safety come from? Do you think the government looks to other countries?

- Participant: No idea.

Interviewer: Or at research?

- Participant: I don’t know because I did not go to school. Some things are for the elites.

Interviewer: We know other countries use enforcement cameras, where people get a fine immediately if they speed or run a red light – do you think we can do such a thing in Ghana?

- Participant: Yes

Interviewer: Why?

- Participant: This is because is like here, if one is installed it will check over speeding. And if someone does something wrong on the road the camera will capture it.
- Interviewer: What mark will you give the government on a scale of 1-10 with 10 being the best? Participant: 6.

Interviewer: Why that mark?

- Participant: This because the government has done well but we can’t say that he has done all that we need for us
