# Supplementary material for: “We are pleading for the government to do more”: Road user perspectives on the magnitude, contributing factors, and potential solutions to road traffic injuries and deaths in Ghana
Source: PLoS One. 2024 May 24;19(5):e0300458. doi: 10.1371/journal.pone.0300458 (PMC11125548; doi:10.1371/journal.pone.0300458)
Supplement: S2 File — (ZIP) [file pone.0300458.s002.zip › Transcripts to share/Participant_112_vulnerable.docx]

**Participant Number: 112**

**Language: Twi**

**Type of hot spot: Urban**

**Sex: Female**

**Road user type: Pedestrian (hawker)**

Interviewer: How do you get to work?

- Participant: Over here I sell pure water [sachet water]

Interviewer: For example walking, public transport (trotros), motorcycles, cars, taxis, trucks, riding a bike, tricycles (i.e., pragya)

- Participant: Walking.

Interviewer: How would you describe this area to others as far as accident is concern or car knocking people down? Is this road busy?

- Participant: Over here car knocking down people is not good at all.

Interviewer: Is it many or small?

- Participant: It’s too much.

Interviewer: How big of a problem do you think accidents are here?

- Participant: Problems about accident in the area is worse.

About two weeks ago my brother was knock down by a car. When we took him to Kwalabu, he died there.

Interviewer: What do you think causes accidents here? Road conditions (such as potholes, lack of sidewalks), abandoned/broken down vehicles, over speeding, wrong overtaking, traffic.

- Participant: Road condition. There is no sign post to guide stranger drivers to their root. Drivers who are new to the road have been asking us to direct them to their destination. We pled with the government to put up road signs to guide new or foreign drivers to their destination. I believe that will help reduce accidents.

Interviewer: What about over speeding.

- Participant: Some drivers when they are descending from up there. Instead of slowing down they wouldn’t they come with tough speed. When they are coming, they don’t know that where I have reached there is a pothole there so I have to slow down. So, sometimes when someone is going to Accra and other too is coming to ofankor barrier they then crash to accident.

Interviewer: What do you think decreases the risk of an accident?

- Participant: It is my plea that the signboard is of very important. And the road which is under construction to it expansion when they finish it will decrease accident a bit in this area.

Interviewer: Are there some people who are more likely to get into an accident (for example: children, hawkers)?

- Participant: Over here, when there an accident it does not affect the children but the adult. Also, when there is a traffic jam, we the hawkers are mostly knock by motor riders. The drivers always drive with care when there is a traffic jam. Again, the adult who are mostly knock down by cars, are those listening to call while crossing the road.

Interviewer: Which age of children?

- Participant: Over here children are not affected in an accident

Interviewer: Sometimes personal stories can make road traffic problems more real. However, we know this can be sensitive. If you feel comfortable, can you share a story from an accident with me? Your own or someone else you know?

- Participant: I have seen a lot of accident here. In January [2023] I will be five years over here ever since I started selling by the road side. Therefore, I have seen a lot of accident here. Somebody might be on his motor bike and all that you will see is a car will suddenly crash him to cause an accident. Also, two weeks ago just as I said my brother was knock down here. According to eye witness he had already finish crossing the road and due to wrong overtaken at wrong side of the road he was knock down by that car.

Interviewer: Can you tell me of a story about a child getting in an accident on the roads, if you have one?

- Participant: Over here, I haven’t seen that a car has knock down any children but sometimes it’s adult who has been knock down by car.

Interviewer: Now, let’s talk now about the police and their role. What do you think about the police’s enforcement of laws now? For example, speed, motorcycle helmets, unlicensed driving, broken vehicles Do you think this affects crashes?

- Participant: Ok if they bring the sign board here, that is the first one. The drivers who drive without license the police has to arrest them, and not to take bribe of ten cedis (10.00) and then leave them to go free. Also, I will plead with my brothers, moto rider that they should wear their helmet because it will protect them. Once I saw a motor rider crash with a car and he fell off from his moto bike. Had it not been the helmet by now he would have been a dead man. Again, some of the policemen don’t fulfill their duties assign to them. They come and all that they do is chat with each other and then go back to their office. They don’t help check pedestrian crossing the road and other traffic related issues. Lastly, I will plead with the police again to help us when any vehicle break down on the road, they should tow that vehicle with immediate effect. That one too will help reduce road accident.

Interviewer: if you had the power, what would you do to change the situation here?

- Participant: I will build a road sign along the road to caution driver on speed limit to say when you are on high ways your speed limit should be 30, when you get to town your speed limit should be say 15, And I will make police men to check that. Where we live when traffic jams the drivers some don’t follow the queue and will be over taking the other cars. we will plead with the government to bring police men there. We, the area guys men and women have turn ourselves into police men to be directing traffic in the evening. If you don’t join the cue and you over take ah! every day we’ve been breaking some people’s glasses, destroying people’s cars for we have told them that if you get there slow down, people live there, some sell by the road side so you should slow down with your speed. There is a speed limit sign board there which says you shouldn’t speed above (30) but for the driver we don’t know their mind set. So, we will plead with them when they get there, they should slow down their speed for us. over speeding kills and causes accident. Last time in the case of our brother who has already cross the road but because of over speeding and over taking we have lost our brother who will be buried on this Monday. So, we plead with the drivers and those who beat up drivers when they knock down some people. If you kill the driver the person for which reason you are beating him to death will not wakeup. So, we plead with them that it’s an accident so if a driver knocks down someone it’s an accident, they should not beat the driver to injured him but should report the case at the police station.

Interviewer: Once an accident does happen, What do you think causes people to die or get hurt, compared to just getting into a crash without getting hurt? For example, what about the condition of the vehicle or trotro makes it more likely for a severe injury or death? Like seat belts not working in cars/trotros, cars being old and not having air bags, position of seats, crowding

- Participant: We will plead with drivers to wear their seat belt because the manufacturer knows it’s importance that is why he fixed them in. We will also plead that when they are fixing the seat, they should position the seat with intervals. Some of the cars their seat is so close that when it falls in the pothole your knee will hit the back of other seat due to crowding and wrong position of seat. At sometimes too your head will hit the front seat. So, before you get to the house your whole body will be paining you. All that is due to lack of seat belt and crowding seat. Therefore, to me I think that is what causes people to get severe injury or die in an accident.

Interviewer: Generally, which people typically to get injured or die in an accident? For example, pedestrians, children, motorcyclists, bicyclists, hawkers, those without a helmet, those who do not use seat belts.

- Participant: Motor riders who do not wear helmet when the accident occurs, they normal get severe injuries and we those who sell in the traffic (the hawkers) we too get severe injuries. And the children too when they are knock down by car they get severe injuries. But for us who sell in the traffic we are mostly affected.

Interviewer: It seams you did not understand my question well what I mean is when the accident occurs which people for example, pedestrians, children, motorcyclists without a helmet, bicyclists, hawkers and those who do not wear seat belts. Choose one.

- Participant: Motor riders who do not wear helmet.

Interviewer: What about the environment (such as the roads) makes it more likely for a severe injury or death? For example, abandoned/broken down vehicles on the road, lack of sidewalks, potholes, traffic volume on roads.

- Participant: May be somebody might be new to the road and has never drive through this road before. His first day of driving through the road and doesn’t know there is a break down vehicle on the road and when a car break down on the road, we have a board (warning triangle) that we put down on the road for an approaching car coming light will reflect to alert the driver about the danger ahead so as to slow. But for some drivers when their car breaks down, they wouldn’t put down that sign board to alert drivers so unknown driver will come and crash it. This sometimes causes accident here.

Interviewer: What can be done to reduce the number of severe injuries and deaths here?

- - Participant: Oooohh! what we must do is that when crossing a road don’t make call. When crossing the road watch out carefully turn right and turn left before you cross. Some don’t watch out before they cross. So, we will plead with our brothers drivers that when they are driving and they reach town they should slow down. Moto riders should wear their helmet when riding. Again, moto rider should stop rough riding and ride with care.

Interviewer: When people get into an accident, or get hurt, what happens? For example, do people call the police? Do people come help? Does an ambulance come? Tell me about what happens.

- - Participant: Yes, it the people who called the police. But after their arrival they don’t do enough. It is us who will stop a taxi to convey the injured passengers to hospital. The challenge here is that some of the taxi drivers say they are afraid to stop because after helping that patient to the hospital. When they call any of their relatives they don’t come and they become stranded at the hospital. The last time a car knocks down our brother and we took the patient to 37 military hospitals, when we call the police and when they arrive if we had money, they would have followed us to the proper care of our brother but because we did not have money, they made our case to be a foolish case. Until DC from our home town came to our aid to put thing in other. So, the police when you call them, they come at their own will.

Interviewer: When you call an ambulance, do they come?

- - Participant: No, they don’t come.

Interviewer: If you had the power, what would you do to improve care after an accident? For example, increasing number of ambulances, training people around in first aid

- - Participant: If I had power, I would first of all speak with the police and the Ambulance service about how to go about with their work. I’ll discipline them if the need be to make sure they all work as they should.I will increase the number of ambulances in the country.

Interviewer: In your opinion, how much of a problem are accidents in Ghana?

- Participant: Accident is a very big problem to us. When accident occur before you its scarry but when it happens behind you the story is different.

Interviewer: Does the government consider your views when they make decisions on road safety?

- Participant: Yes

Interviewer: What is the government currently doing to reduce accidents? For example, speed bumps, enforcement by police, pedestrian bridges, education campaigns.

- Participant: The reason why I gave a positive answer to the previous question is because, there was an accident here the last time which caused a very long traffic that people were not able to go to work. Some reporters from UTV and other television station came around to interview us and I really poured my heart out to them concerning the accident. I explained to them the reasons for frequent accidents in the area and as you can see now, the road is being expanded to help reduce accidents. This actually proves to me that the government really listens to us.

Interviewer: Have you heard of those?

- Participant: Yes, I have heard of them.

Interviewer: Have you seen those?

- Participant: Yes, I have seen it

Interviewer: Why do you think the government chooses these? For example, speed bumps, enforcement by police, pedestrian bridges, education campaigns Are they considered better?

- Participant: Yes, they are.

Interviewer: Are they cheaper? Do you think the government considers cost when they pick what to do?

Participant: [no answer]

Interviewer: Where do ideas about road safety come from? Do you think the government looks to other countries? Or at research? Participant:

Participant: At research

Interviewer: We know other countries use enforcement cameras, where people get a fine immediately if they speed or run a red light – do you think we can do such a thing in Ghana?

- Participant: Yes, we can.

Interviewer: Why?

- Participant: It help check over speeding our road. Over speeding drivers can be arrested and fined.

Interviewer: What mark will you give the government on a scale of 1-10 with 10 being the best?

- Participant: 10

Interviewer: Why that mark?

- Participant: This is because he has done well.

Interviewer: Finally, our last question for you is: If you had the power, what would you do to reduce accidents, injuries, and deaths on the roads nationally?

Interviewer: What would you do for pedestrians?

- Participant: The first thing I will do is that anytime I construct a road I will build footbridge for the pedestrians. I will also construct pedestrian walk way by the road side and zebra crossing where necessary.

Interviewer: What about motorcyclists?

- Participant: [no response]

Interviewer: What about for children?

- Participant: [no response]

Interviewer: Is there anything else about crashes, injuries, or deaths on the roads that we haven’t discussed today that you would like to tell me?

- Participant: [no answer]

Interviewer: Thank you for your time and participation in this important work.
