# Supplementary material for: “We are pleading for the government to do more”: Road user perspectives on the magnitude, contributing factors, and potential solutions to road traffic injuries and deaths in Ghana
Source: PLoS One. 2024 May 24;19(5):e0300458. doi: 10.1371/journal.pone.0300458 (PMC11125548; doi:10.1371/journal.pone.0300458)
Supplement: S2 File — (ZIP) [file pone.0300458.s002.zip › Transcripts to share/Participant_113_vulnerable.docx]

**Participant Number: 113**

**Language: English**

**Type of hot spot: Rural**

**Sex: Male**

**Road user type: Pedestrian**

Interviewer: How do you usually get to work? do you use this road quite often?

- Participant: No I use this road every morning and evening,

Interviewer: So you use it and go to work? What again?

- Participant: I use it to go to work and farming, We use it for travelling, market and co

Interviewer: Okay, so How do you describe this area, is it a big area? is it like a busy area?

- Participant: Yes of course, is a busy area, sometimes hasn’t normally occur within this area

Interviewer: So how big is problem of accidents in this place?

- Participant: Well like flatten tire, our tire normally had a flat or something like mistakenly some of the drivers like sleeping. (inaudible)

Interviewer: What do you think can decrease the risk of accidents in this area?

- Participant: So decreasing of accidents is lets talk to the drives, like when you know that you are a sleeping you should going slow slow and over taking normally that is the important thing over taking it used to be causing accident for us here.

Interviewer: Ok, so Which people are more likely to be involved in an accident here? Is it children, or hawkers or people going to farm? Which people are likely to be knocked down?

- Participant: Traders,

Interviewer: Okay, have you seen any child knocked down here before?

- Participant: No

Interviewer: Okay, so Do you have any personal story that one day you witnessed someone being knocked down around this area that you can share with us?

- Participant: It was last year that we have seen one person the fellow from Burkina and then he was on top of animal truck and then he fall down and died. And that’s what I can only.

Interviewer: Okay, okay but you don’t have any experience of a child being knocked down?

- Participant: No.

Interviewer: Now let look at the police and their role in enforcing laws, How do you see their work here?

- Participant: As for the police patrol so nice, they are are doing well each and every time night, morning, afternoon I use to meet them here patrol,

Interviewer: Ok okay, do they check about speeding? Or wearing of helmets? Or license or unlicensed driving? And broken vehicles? Do they check about those things?

- Participant: Yeah, they are checking helmets, number plates, speeding, all, They have been checking and they have been catching people for helmets, those that are picking two two or once and they don’t have helmet they use to judge.

Interviewer: Ok, so do you think that one affects the number of accidents, if the police are doing their work the accident reduces or if they are not doing their work the accidents is not reducing?

- Participant: If there are doing the work the accident reduce.

Interviewer: If you have the power, what would you do about this place?

- Participant: Well if I get power I will let them increase the patroling, stoping the accidents and other things

Interviewer: So when you say you will make them increase the patrolling, you mean the police patrol, so then what would happen? What would the police do?

- Participant: they will be roming if anything badly in the road they will be stopping it.

Interviewer: Uh-hu, so speeding? Or

- Participant: Yes, If there is over speeding, ~~riding~~ without helmet or ahhaa those things… loading heavy [inaudible] people and the trucks.

Interviewer: So anytime there is an accident here, what happens? Like? If you get into a crash, okay, is it the condition of the vehicle or the tro tro makes people likely to be injured or makes people die, to die? What do you think happens?

- Participant: Sometimes mistakenly a flat, a tire can get flat, and then the driver will fall, and sometimes too, due to the speeding,

Interviewer: How about seatbelts not working or tro tros? Or car being old car?

- Participant: All these things include old cars, ahaa.. all include.

Interviewer: So generally, which people are always involved or get injured in an accident, in terms of is it people who are crossing this road or people riding motorcycles?

- Participant: No no, people riding vehicles.
- Participant: No, no, Those riding vehicles are those use to get accidents here, not motors and children crossing no

Interviewer: Ok so how about the environment? Do you think that potholes, speed ramps, and co, do you think they are working to reduce accidents? What do you think about those speed ramps?

- Participant: Yea, the speed rump is helping, the speed rump is helping cuz when you are top speed and you get to the speed rump you will reduce your speed and then and pass. Its helping. The speed ramps.

Interviewer: So what can be done to reduce those things? Like the speed ramps? Sometimes they are not done well, what can be done?

- Participant: Unless we talk to the road safety ~~commission~~ committees so that they will re-do or do new speed rumps for us.

Interviewer: Okay, so anytime there’s an accident in this place, what happens? do you call the police, do you call the ambulance, what happens? tell me

- Participant: That is the first thing we will call the police when the police came and then there is a high accident we used to call ambulance to pick them to hospitals.

Interviewer: So, when you call the ambulance do they come?

- Participant: Yea, they are coming

Interviewer: How long does it take for them to come?

- Participant: well if they call ambulance assuming here to Buipe less than four minutes they will be here?

Interviewer: So who gets an ambulance and who doesn’t get an ambulance? Like if the ambulance people we call them to come, when they are coming, do they look at the person before they will now come and the person is a big man or he is a rich man

- Participant: No the moment they just call they will come and pick and they will follow along

Interviewer: So, if you had the power, what would you do to help in that situation in terms of the ambulance people coming? And then training people to do first aid when there is an accident? What will you do if there is an accident? Do you want more ambulance people? Do you want more people to come and be given first aid to treat those people who have gotten injury?

- Participant: If I get power the first thing I will do is that I will talk to the ambulance anytime that they call them they should be in a hurry and after that I will talk to the hospitals the clinics anytime they brought the injuries they should be active and then for treatment.

Interviewer: Okay, so, in Ghana, how do you think the problem of accidents is? Is there a problem of accidents in Ghana?

- Participant: Yeah, so the problems in Ghana is we should stopping taking like a driver taking alcohol before driving sometimes some people are driver and you are smoking all other things are giving us accidents, so all those things we suppose to reduce or we should leave all as a driver.

Interviewer: And if government is making decisions on road safety, road safety, if the government is making decisions, do they consider your opinions? Do they come and ask about your opinions before they do anything here? Barrier or anything here?

- Participant: No, because I could remember Domeabra these accidents were many and then we were communities people were trying to put a speed ramp and it was it tend to a soldier chase, ahh

Interviewer: A soldier chase?

- Participant: Yes, that we didn’t informed and we were trying to put that speed rump, the soldiers came there and were beating people and then the stones and the gravel that they fetch to make that speed rump they let them commot everything in there. Uh huh.

Interviewer: So what is the government doing to reduce accidents in Ghana? What do you think? Are they doing speed ramps? And then, doing bridges? Have you seen those things? And have you heard of these?

- Participant: Yeah, yeah, I’m seeing them and I’m hearing too.

Interviewer: So why do you think the government be choosing speed ramps? Is it that they are cheap?

Interviewer: Or they are better or what do you think? Why do they pick those things? Do they look at the cost of it? If it is less cost then they will do it and if it is high cost they will not do it? What do you think?

- Participant: Well I could see that if it is high cost, anything I believe government will do

Interviewer: So, where do you think government get these ideas from – doing of speed ramps, bridges, where do they get ideas from, is it from different  ~~in terms of road safety? Do you think they look to other~~ countries or research?

- Participant: Yea, I will say from research and from different countries because we have been seeing some of them. Uh huh.

Interviewer: So we have seen that in some countries they have speed cameras so if a car is going the camera is on it so if it goes some limit then overspeed then the camera will capture the person and then they will arrest the person do you think we can do it in this place, and in Ghana?

- Participant: Yeah

Interviewer: And then why do you say yeah?

- Participant: Yeah, if we wish we can do it because now no there is nothing in the world that Ghana want to do it and cant if only they want they will do it, I know Ghana now ~~has gone~~ go far.

Interviewer: Ok, okay, What mark will you give this government from ~~on a scale of~~ 1-10, 1 is they haven’t done anything, 2 maybe they have done something small, up to 10. 10 is that they have done everything perfect, what number would you give them from 1 to 10?

- Participant: [laughs] I will give them five (5),

Interviewer: Why do you say 5?

- Participant: Haha, I say five because now the things are increasing

Interviewer: What things are increasing? Is it accidents?

- Participant: No, while [inaudible] is increasing, like during Christmas season like this in Ghana we used to experience accidents, uh ah there is so many things that go high high and high like those things doesn’t suppose to do that but now we are seeing it do that.

Interviewer: Okay, so, This is our last question if you have the power, what would you do to reduce accidents deaths in this road for people passing, crossing, for people riding motos, and then for children? What will you do?

- Participant: First of all if I get power, I will let the owner of the childrens they should take care of them very well, and then I will let the speed rumps everywhere needed speed rumps they should make sure that the speed ramps should be there and the driving, drives dinking or smoking and be driving I will try to stop it.

Interviewer: And then people crossing the road?

- Participant: People crossing the road I will tell them if you are to cross you should have time, check your left and right if there is car you should exercise patience until the car move before you get a way to cross.

Interviewer: So do you have anything to say about what happened here or anything about accidents that I haven’t asked you that you want to tell me

- Participant: Aaah… what I have to say is that I will beg so that from this community, the Sawaba to get two or one speed rump there aahaa…

Interviewer: Thank you for your time.
