# Supplementary material for: “We are pleading for the government to do more”: Road user perspectives on the magnitude, contributing factors, and potential solutions to road traffic injuries and deaths in Ghana
Source: PLoS One. 2024 May 24;19(5):e0300458. doi: 10.1371/journal.pone.0300458 (PMC11125548; doi:10.1371/journal.pone.0300458)
Supplement: S2 File — (ZIP) [file pone.0300458.s002.zip › Transcripts to share/Participant_114_vulnerable.docx]

**Participant Number: 114**

**Language: English**

**Type of hot spot: Urban**

**Sex: Male**

**Road user type: Motorcyclist**

Interviewer: Please thank you for your time can you tell me what kind of work do you do, and do you use this road often?

- Participant: Oh, this road is just my business trip road I have been going in it up and down always, I have been using it always up and down, everyday from my village I use this road to go to Buipe and come every day.

Interviewer: So how will you describe this road to people, I mean is this road a busy road?

- Participant: Oh, I actually you people came here and you can see this is the main road and all the time the road is just busy every time at all it doesn’t matter this day is not busy everyday the road is just busy like that.

Interviewer: Are accidents a problem here, or how do you see the issue of accidents in this road?

- Participant: Yea, as for the accidents deer…. That is the biggest problem that we have here because there accidents which has been happening here which some of the accidents you just cant or will not understand how the car managed to run down. On the there is one too oh you come and you see that this is the fault that is why you went and fell down

Interviewer: o the faults are what? Is it Potholes or broken down vehicles or over speeding or wrong overtaking? What are the problems here?

- Participant: To be frank, there are no potholes here. But, I think maybe it will be overspeeding or breaking of tyres or sleeping or drinking while driving or driving and making calls. I think that’s why they normally fall down here.

Interviewer: Ok, so what do you think can decrease the risk an accident here?

- Participant: Hmmm… I think if you talk to them like they should stop the over speeding I think it will be better because when you are over speeding and immediately you car went down or your tire just blast I think you will not get any control you will be busy before you realize you are down.

Interviewer: Which category of people are mostly affected during accidents here? Is it children, farmers going to farms or who tell me?

- Participant: Hmmm…. For that one even recently just near our village here Sawaba the car just run into a young boy just kicked him down just recently it will not be up to one month koraa… just here, about 15 years old boy.

Interviewer: Personal story will help us understand the situation better, if you feel comfortable can you share with me a story of an accident that you witnessed either someone or you yourself?

- Participant: Aaah for the accident we have been here and immediately when happened we always pick our motor bikes and run to the place try to save some of the people…… This accident I’m going to tell is very terrible because the car run into a flat tire and went and hit one of this poles the street light poles and the light come down to this thing…. So we have to call VRA to off the lights the main lights before people can get near to them and by the time we went near they do all died most of them went off and we call ambulance to come and pick them some go to Buipe and the rest some to Tamale direct.

Interviewer: How about a child, do you any story of a child?

- Participant: Yea, the guy was crossing to farm just cross the roadside to go to farm and the car I can say yeah crossing to the farm to do something for the house to feed himself and car just run into them, just killed him like that.

Interviewer: Now let talk about the police and their work, I can see they have a number of barriers here, what do you make of their work?

- Participant: The police as for them they have been patrolling, but the drivers as they know where the barriers are, wherever there is no barrier when they are coming they always gas before they reach the barrier and they slow down the barriers the police will give them way and they pass.

Interviewer: Do the police check about helmets, unlicensed drivers, over speeding and over loading?

- Participant: Eeerh… they always check, they always check about that things that normally happen even as you can see today is Monday when you go to the barrier you can see motors, they collect them and pack them until they bring their helmets and things before, they give them back.

Interviewer: So do you think all this affects crashes? What the police is doing?

- Participant: As for the police work, it does not affect anything. It does not affect the accident. But it’s just on the roadside moving. Because when they see that there is police here, before they get there they have to slow down. They will not speed there for them to see that they are speeding. What I am saying is that the police looking to make sure people are not overspeeding and those things we talked about, do those things decrease accidents here. Yea, it decreases accidents here why because they always during the night like this they always pick their car on the road side walking around for that all of them will be very careful running down slowly to where ever they are going.

Interviewer: If you have the power, what would you do to change the situation here?

- Participant: Eeerh…. If I get power deer.. the only thing I will add is they should try and give us more speed rumps to the nearing villages I think that will help for the over speeding and that kind of some petty petty accidents that has been happening.

Interviewer: When an accident occurs what happen? What do you think causes people to die or injured anytime an accident occurs, for example is it the condition of the vehicle that makes more likely for a severe injury or death?

- Participant: For that one some of the Trotros they always take over loading that over loading it can make accident occurs and car went down smashed each other to get each other wounds or some will go dead that one too is involved.

Interviewer: Generally, if people typically get into an accident, which category of people are mostly affected? For example, is it pedestrians, children, motorcyclist or hawkers? Is it also those with helmets or without helmets or those who doesn’t use seat belts?

- Participant: Yea, all of them, all of them normally get but it is not all the time all of them get accidents it normally happen once a while that is why I said earlier on if I got the power I will try my possible best to make them do the speed rumps so that it will help the communities to avoid that kind of accidents.

Interviewer: How about the road environment? For example, broken down vehicles on the road, potholes, lack of sidewalks and traffic volume?

- Participant: Yeah, if there is a road and there are potholes it is a must that there will be an accident because one car will come and slowdown and other car will come and hit the back. I think that one too they have to check on it to make sure all them are not like that.

Interviewer: What can be done looking at this situation what do you think can be done to reduce the number of severities and deaths here?

- Participant: Hmmm… that one deer… as at me like this the normal thing to done is to talk to the drivers and to the motor riders talk to each other to slow our speeding, to slow the speeding that is the most important thing happens here.

Interviewer: You talked about some accident scene that happened here about car crashing into the street light poles, so I want to find out from you any time there is an accident that occurs in this road what happens? Do you call the police? Do people come to help? Do you call ambulance? Tell me what happens?

- Participant: Yea, immediately we heard that there is an accident here even the car is there right now, immediately we heard there is an accident we quickly took our motor bikes run to there as we see how the people is we call the police, police came and there are people who stand at the road side stopping the cars and they will be going slowly before they will call the ambulance to also come.

Interviewer: Ok, so when you call an ambulance do they come and how long do they take before they come?

- Participant: The ambulance you know from here to Buipe is not far like that so they always take like 5 minutes or 6 minutes before they will get here.

Interviewer: If you call the ambulance, do they just come because you have called them, or they are looking at the caliber of the one that call them or the vehicle that call them or they just come?

- Participant: No, immediately you call the ambulance to come, we don’t check that things because we need to save a life, we need to save life so the ambulance immediately you give them a call they will come to the place wherever you give them the call.

Interviewer: So, if you had the power, what would you do to improve care after an accident has occurred? Will you be increasing the number of ambulances or training more staff to give first aid?

- Participant: That is the major thing that I will do, the ambulances I can say they are not enough for us here, the ambulances are not enough when I have the power I will make sure that I increase the number of ambulances to patrol the road very well for us, immediately there is an accident, I can see this one here we put some people down the ambulance went and came back before it took the other ones, and the police car took some also and go, I think that too is not fair if I get the power I will make sure I will add the ambulance so that everything go safely when they send them.

Interviewer: So, we are talking in general, in your opinion and your own estimation are accidents a much problem in Ghana?

- Participant: Yea, I think there are problems with accident in Ghana because our leaders they have to know that this is the road all of us are using and this is the road somebody is using to get something to feed him house so even this road there are some things they can do to avoid everything.

Interviewer: government is doing something about road safety do government consider your views and opinions when they make decisions on road safety?

- Participant: Yeah, I can say they do because as you people are here they do but eerh.. the problem is they will come and ask the questions and go at the last you will not see anything. They will come and ask you will answer them give them words and they will sent it and that will be all.

Interviewer: What do you think government is doing currently to reduce accidents in Ghana?

- Participant: I have witnessed speed rumps and bridges, they are doing speed rumps and bridges but I think the ones they are doing is not enough at least one village they will just go and do one speed rump and that will be all at least each village there is supposed to be two or three speed rumps before you cross or leave the village or where ever you come from in to a town is not suppose you will go and meet like Domeabra like this you will go there are two speed rumps there are supposed to be three, one at when entering, one at the middle, and one out.

Interviewer: What do you think government decides to do speed ramps in some places and not all places? Do you think government considers cost or what?

- Participant: For that deer.. I cannot say because of the money or is not because of the money because somebody might even give somebody the money go and do this job and he will go and do whatever he wants and go out with, before you realize whatever you say at least they will say he should do three speed rumps, four speed rumps then he will go and do three or four and go, that one I cannot say is be the government issue because he has given out to go and do the job.

Interviewer: Where do you think government get their ideas on road safety? Is it that they look to other countries or at research, do you have any knowledge regarding where all these ideas on road safety?

- Participant: I think they do their research, they do their research to also do their things all, and they also go to outside to see some of this things and they also come to Ghana do it.

Interviewer: In some countries they use enforcement cameras to monitor the speed of vehicles on the road, and people get fine immediately if they over speed or run a red light- do you think we can do such a thing in Ghana?

- Participant: Yes, Ghana we can do it, we can do it even when we do it that will help some kind of accidents that is normal happening, because you will see a young boy and they will give him a car to drive everything he knows it to over speed and go fast he does not think about the people he carry or the life he is taking over eerh, that thing if they say they will do it, they can do it.

Interviewer: So If you are going to rate Government, what mark will you give government on a scale of 1-10 with 10 being the best and 1 being the poorest? What mark will you give government?

- Participant: I think I will give the government five (5), because he has been doing the job but not completely, he has been doing but not complete.

Interviewer: This is our last question, if you have your own power, what will you do to reduce accidents, injuries, and deaths on the roads in Ghana? What will you do for pedestrians, motorists and children?

- Participant: Hmmm if I get power I will employ more securities to help the nation, the police I will tell them that immediately they stop a car they should the driver some advises that they should be going slowly because they are carrying lives, and the motor bikes they should try to hold helmets and that things to help them selves so that there will be no harm to them. Those riding the motors they are supposed to be holding helmets before they ride the motor, if you know you are going to a far place, not even a far place before you want to move your bike you have to check that oh am I holding helmet, am I wearing helmet or where am I going is it safe, or is it like something there, you have to check all those things before you move. For children crossing the road, that is why I talked about the speed rumps, you can say whenever they want to cross the road they should make sure they check before they cross. Some of them when they come they always cross, for the crossing if there is speed rump on the place whenever the car is coming either yes or no the car will slow down everything will slow down and everything will go off.

Interviewer: Is there anything else that you want to add to this our conversation regarding crashes, injuries, and deaths on the road that we haven’t talked about today?

- Participant: What I have said is ok for me that is what is in my heart, and I spoke out.

Interviewer: Thank you so much for talking to us, we have really appreciated your time and your effort.
