# Supplementary material for: “We are pleading for the government to do more”: Road user perspectives on the magnitude, contributing factors, and potential solutions to road traffic injuries and deaths in Ghana
Source: PLoS One. 2024 May 24;19(5):e0300458. doi: 10.1371/journal.pone.0300458 (PMC11125548; doi:10.1371/journal.pone.0300458)
Supplement: S2 File — (ZIP) [file pone.0300458.s002.zip › Transcripts to share/Participant_115_vulnerable.docx]

**Participant Number: 115**

**Language: Dagbani**

**Type of hot spot: Rural**

**Sex: Male**

**Road user type: Motorcyclist**

Interviewer: What form of means do you use here?

- Participant: I use tricycle on this road

Interviewer: To where?

- Participant: I use it to Buipe market and Yapei market

Interviewer: Looking at this place, is it busy all the time?

- Participant: yes, people passes here. It’s a busy road especially on market day and on Mondays, this road on Mondays looks too busy.

Interviewer: Mondays, what days are that?

- Participant: It is a Market day. Buipe market days

Interviewer: Looking at accidents and injuries on this our road~~s~~ is it a big problem?

- Participant: Yes, is a big problem, because on Mondays, you can be using this road and the big big trucks that plough this road can stop in the middle of the road ~~to due problem or~~ due to mechanical fault with the truck, then they will bring big big rocks to work on their truck then after repairs they just leave those rocks on the road and go, they don’t have time to remove those rocks from the road, then you can be using your tricycle coming and climb on it, our tricycle is not strong so if you are carrying people in your tricycle you all can fall on the ground leading to injuries and sometimes even deaths. Just last Monday here it occurs one of the big buses, blew a horn on a tricycle driver, then he panicked and veer off the road leading to accidents and some injuries. I have been seeing these.

Interviewer: So, accidents are a big issue here?

- Participant: Yes, accidents are a big thing here,

Interviewer: What causes it and why are you saying so?

- Participant: hmm what is causing it is there, in some places they need speed rumps and there are no speed rumps because of their absences it can be attributed to this problem. And another thing sometimes some buses carrying passengers have issues with their tire but they will say let me go and manage if I get money I will change the tire, front tire bursting in this place are many here those one are many here those ones are many.

Interviewer: If you see, what are we going to do to reduce the accidents, the motor accidents to reduce?

- Participant: hmm to me how we should look at it, they should be laws if you go to pick a passenger and the conductors people check your tires if your tires are not in good condition they should not allow you take people, you should be patient if you check your car and is in a good condition then you can carry passengers.

Interviewer: So here if accidents happen and people getting injuries which people are affected, children, or hawkers or which people motor riders?

- Participant: For these vehicles, the most affected ones are traders. those people that goes for trading on market days, after trading on the day is time to go home, now the buses has loaded their goods on the buses but there is not empty bus to use unless you join the same bus with your goods, so now the bus is carrying people at the same loaded goods and weight is more than the tires, this is what is frequent here and most of them dies, a lot of them dies here.

Interviewer: How about children, are children part?

- Participant: yes, children are part

Interviewer: Have you ever seen or witness some of the accident or have you ever been told about it, can you narrate to us how it happens?

- Participant: Yes I have seen, just recently here there was one bus that came from Sunyani, it was carrying a lot of market women and burst its front tire the people that we remove and they were dead were up 5 people we are not talking of people that follow and died at the hospital we don’t even know, but those we remove here there was a child among adults and all those that die were 5 them.

Interviewer: How old was the child, around what age 5 or 7 can you tell?

- Participant: looking at how the child was and to me as human, I can say he/she could have been up to 9 or 10 years of age.

Interviewer: Okay, have you seen a vehicle ~~car~~ knocked down a child here, and can you tell us something about that?

- Participant: Yes, it not up to a week, just recently a car knocks my sister daughters here and we send her to Tamale when we got Tamale, she died at the hospital, yes just recently it has not been while.

Interviewer: Now let look at the police and the work they do here, they are at the barriers, what do you think about their work regarding accidents, what are they doing regarding preventing of accidents and other things?

- Participant: Eiih! the way they are doing their work, we haven’t gone to school, we don’t know what they should do but the way they are doing their work I don’t think that is how they are supposed to work, a bus will be carrying an overload and will be by-pass a police check point you police you have to tell him the way he is going he should reduce, how he is going is not good, I don’t know they tell them or they don’t tell them it doesn’t look like they are carrying human being.

Interviewer: Looking at those situations the police not doing their work thus that affect the number of accidents here?

- Participant: Yes, this contribute because if I were a police officer, I will see some drivers and I know they have fall foul to the law, some drivers even drink and drive and the police can sense this just interacting with him and in that situation I will just cease the bus until the driver is now ok before I will let him go.

Interviewer: If you have the power and you are the one is in charge here what will you do?

- Participant: if I the power right now and I am the one who talks and is final I would just order a law on this road people using this road especially drivers if they pick rocks to work on the vehicle ~~buses~~ the police know them and have their ~~buses~~ vehicle numbers and refused to pick the rocks and motor rider or tricycle rider comes to climb it and it leads to accident first, whatever the accident cause I will let them call the said driver and cease his car until he pays for everything before we allow the person to use the road again. Next time he won’t do that again. If you use it and finish remove it from the road. If I have power that is what I would do first because it is causing us a lot.

Interviewer: When accident happens, what causes people to be more injured?

- Participant: If a car falls…truth be told what is making people to jet injuries I blame everything on the speed of the vehicle, sometimes we see people move in some speed and you wonder they are carrying human beings, and sometimes too some passengers too will be asking driver to speed more, meanwhile if he goes more speed you get accident many people will be affected.

Interviewer: How about no seat belts or seatbelts not working, or overcrowding in the cars old cars, that’s that affect the number of injuries?

- Participant: Those things causes a lot of accidents, what I was telling you concerning a vehicle that fell here the other day, when the bus feel some people were forcefully thrown through the window of the bus so if there were seat belts it would have prevented that, some child even went as far as hitting the electric pole on the side of the road leading to fractures and other damages. The seat belt could have prevented this, so there should a law on the seat belts and drivers made to adhere to it, if you are on the road as a driver without this seat belt law enforcement people should hold you.

Interviewer: Looking at if a car get accident, is it those people who walks around the road that are affected more, or children crossing or people selling foods on the road?

- Participant: Anytime time there is an accident like in this road, it affects a lot of people, if you witnessed an accident here, I tell you if you are not emotionally strong you won’t want to pass here again, you won’t be able to walk here and if you are passing you can’t go. It affects many people not a single person.

Interviewer: If you look how the road is made does it contribute to more accidents, like potholes does it add to the number of accidents?

- Participant: Yes some of them contribute, we all don’t hold machine the same way. The way someone can speed with motor and fall into a pothole if you are not careful you will fall down, just recently one motor rider after falling into a pothole and got sway off the road and went hit a tree on the roadside. Just last week road officials came and were clearing bushes and trees on the sideways of the road, we don’t know what happened and they stopped midway through.

Interviewer: So, looking at it what can we do to reduce the numbers here?

- Participant: what they will do to me oo any small village whether ~~4 0r 5~~ 3 or 4 houses and is closer to the road here it doesn’t matter how small the village is it should be given speed rumps, any car that is coming and get to the speed rump will know people passes here and reduce the speed of the vehicle, hmmm….people walk across the road here so the driver will have to slow down, this to me will reduce the number of accidents here

Interviewer: If an accident happens, what do they do, do you call police, do you call ambulance, the car you said fell down here what did you do?

- Participant: When the car fell first it was the police came, we move there, and we started begging any vehicle that comes to and take the injured people, some two Pickup cars stopped, and we pick those people who were still breathing and can be manage inside the pickup car, we were there until the whole place were cleared we did not see any ambulance. The ambulance they have people that called them and they go but we and the police are here every day. As for the police they don’t wait at all they will just rush and come, even the police pickup also picks some victims, it picked more up to 10 victims inside their car to the hospital.

Interviewer: If you call the ambulance, do they come and do they take time before they come?

- Participant: if you call the ambulance, sometimes they can tell you that they are in a different location, I don’t have their number, but people that calls them says when they called them, they will tell you they are somewhere.

Interviewer: Okay, so if you call an ambulance and it is coming to pick the victims, do they look at the strength of those involve before coming or they just come?

- Participant: I will say the ambulance people look at the person calling them before they attend to an accident victim, if you don’t have power and call the ambulance people you will suffer, if you call them and they see that you are someone who is capable to will come, they won’t tell you the ambulance is not available.

Interviewer: So, looking if you have power, what would you do to improve the ambulance situation regarding police coming, giving first aid and sending to hospital what will you do, which one will you tackle?

- Participant: if I have power I think I will help the police and increase the number of ambulances, when I told you about the accident that occurred here some few weeks ago……, see after that accident you cant even go near the victims, there were bees around them that fell from the tree all this gadget are needed to be provided by the government to the police and other people, then also clear some of the trees around here the road so that incase of accident the driver can manage small before eventually falling down.

Interviewer: Looking at Ghana, are accidents and injuries a problem in Ghana?

- Participant: This is a big problem in Ghana, you don’t know who will grow to become an important person in Ghana, and who is going to help Ghana in the future he can be a nobody today but somebody tomorrow, will you be fighting for those who have strength and leave the weaklings? If an accident happens and an ambulance comes to pick the person fast it will help, but if the person is left bleeding and before they eventually attend to the person, the person my die on the way or in the hospital.

Interviewer: So if government comes including road safety people doing this things do they consider your views when they make those decisions on road safety or they just come and do and go?

- Participant: They have to listen to you, just look at this road, most at times we have to come out and demonstrate and say we need speed rump here before we can see anything happening just this village in front of us, they came out and did their own speed rump twice and government destroyed it on those occasions then later government came and gave them a speed rump then accidents reduced in that place.

Interviewer: So, what is government doing to reduce accidents in the roads, do they do speed rumps, are police on the road ensuring regulations, what are you seeing?

- Participant: If it is somewhere but here is not there, there is a small corner here Monday or Tuesday and everyone is aware of it including Ghana if the police cannot {inaudible}… they should involve the chiefs around this areas to setup a committee and people that can work and reduce the number of accidents there, anytime a car is approaching that place in the night and sees a light he will become alarm because of how dangerous that place is.

Interviewer: Which corner? Where?

- Participant: Just this corner, the Sawaba corner, it is a very dangerous place even armed robbers knows that corner, so sometimes they do stand at the place in anticipation of an accident it is a problem here.

Interviewer: Okay, if you look at the speed rumps and the things government is doing if they are doing are they looking at the cost involved, or they are just doing and conscious of the money spent why is some speed rumps looking like that?

- Participant: Some of the speed rumps to be frank if I have power, and I gave someone work and the person do it this way, I won’t be happy I will never give such a person any work again, if government was doing that and refusing them work they would have been working and it will be lasting. The constructors just come and do cement alone and within 3 days time there are pothole in it. They are cheap things.

Interviewer: Looking at it that means they are low-cost materials?

- Participant: they are low-cost materials, the materials they are using is low cost.

Interviewer: Okay, where do you think government get these information and ideas and they will come and be doing speed rumps, overhead bridges where do they get the information from, is it that they go to some countries, or they do their research?

- Participant: In my thinking because every market day I uses this road from Npaha junction to Buipe if I say I haven’t pay I will pay tolls up to 45 cedis, I will pay up to 45 Cedis every market day, so my thinking was when they collect this monies they will use it and work on the road but we are not seeing that, that would have improve the road conditions me, I have not been to school but they always when one pays tax those monies goes to construct the road, so I don’t know the benefit of the monies we have been paying on the road.

Interviewer: If you see some countries, they use speed cameras, you know it right? And it capture the speed at which the driver is going, if the way he is driving the car is over speeding the police will know and punish him so that next time he will not do that again, can we do that in Ghana, if you say yes then why?

- Participant: That thing to me that thing would have work, why did I say so, in Ghana if you think you are a hard guy you can ~~there~~ not smoke India hemp in front of police, and he will see and leave you. Ghana can do it and that thing will guard this road, if someone do that and then they sent him home and tomorrow someone do that and they send him home you will see Ghana law will be working, but when someone do that and they arrest him and he gives one million two million and give and then they release the car for him and he will bring the car on top of the road and cause another problem.

Interviewer: If they tell you give government a mark from one to ten, and the one is he hasn’t done anything, two he has done something and up to ten and ten he has done perfectly, what number will you give?

- Participant: at this moment, I told you that me I am a farmer I haven’t been to school, but if I am going to give marks, I will give him out of ten I will give him nothing or 1.

Interviewer: Why do you say one?

- Participant: the reason why I said one, today I am a farmer and I am not well to-do and I go and call a car if he comes and finish ploughing he will tell me prices have gone up, then when I go to buy weedicide, he will tell me when he went to buy prices were high and sell for me high price, that is why I said I will give government one because I don’t eat anything from government.

Interviewer: Our questions are just finishing like that this is the last question, if you have power in Ghana, and you can do anything regarding accidents and accident will reduce what will you do regarding people crossing, people riding motors, what will you do for them to reduce accidents, even for children going to school and crossing roads what will you do for them?

- Participant: if I have power, just as I said if I have the power, you remember the speed cameras, first that is what I will do, then I will make sure all speed rumps are at where they are supposed to be, and then I will also pass a law in this road if anyone fall foul or go against it the law will deal with you. If you used a motor and commit a crime then you will be banned from using motor unless bicycle that is if I have power, if you do this to two or more people then people will learn. I think sometimes some people just want to come to the road and cause crime, then this law will work in Ghana.

Interviewer: How about kids on the road especially school going kids what will you do for them?

- Participant: those kids going to school I will make sure every school has a watchman… if the school kids close from school the watchman will ensure that they cross the road safely, and a law should be put around mothers leaving their kids on the road and kids who doesn’t still know anything shouldn’t be left around the road. If any mother cannot guide the child, then a caretaker should send the kid to school and after closing the watchman will make sure the kids cross the road and go home safely.

Interviewer: What do you have to add to this our conversation, our question are finish, what do you have to add regarding accidents here or Ghana in general.

- Participant: What I want to add, we are begging government just like what I said, they should let the law be working they shouldn’t do things anyhow, it shouldn’t be about this person is capable or influential they should let every law work, everyone should be treated equally before the law and if any one should commit ant road crime the law should deal with him.

Interviewer: Okay, thank you so much.
