# Supplementary material for: “We are pleading for the government to do more”: Road user perspectives on the magnitude, contributing factors, and potential solutions to road traffic injuries and deaths in Ghana
Source: PLoS One. 2024 May 24;19(5):e0300458. doi: 10.1371/journal.pone.0300458 (PMC11125548; doi:10.1371/journal.pone.0300458)
Supplement: S2 File — (ZIP) [file pone.0300458.s002.zip › Transcripts to share/Participant_116_vulnerable.docx]

**Participant Number: 116**

**Language: English**

**Type of hot spot: Rural**

**Sex: Male**

**Road user type: Tricycle driver**

Interviewer: Please can you tell me what kind of work do you do, and do you use this road often?

- Participant: I ride tricycle and I use this road often from here to Buipe most especially on Mondays and Sundays at times we use this road to Tamale to carry animals.

Interviewer: How will you describe this road to someone? Is this road a busy road?

- Participant: It is a busy road because it is a highway so there a lot of vehicles on this road and also motor bikes.

Interviewer: How do you see the issue of accidents in this road? Are accidents a problem here?

- Participant: Yes, accidents is a problem here in fact at times we the ~~motorcycle~~ Tricycle rides we do cause accidents and the vehicles too do cause accidents when you are riding a tricycle instead of being in your path you will rather being on the main road and when a vehicle is coming it can cause accident if you are not careful in the road.

Interviewer: Ok, so what do you think can decrease the risk of being involve in an accident?

- Participant: Education, education because as I’m saying as a rider instead of you to be in your lane as time koraa.. you will see somebody being on the middle of the road with a tricycle you can easily be crashed with a vehicle, you can easily be crashed and at times too you will see somebody leaving his path instead of being on this side when you are going. You are going to Tamale instead of you to be in your side and the other vehicle coming to also be on his side you will see him being on your lane that one too can cause accident, you can easily crashed .

Interviewer: Which category of people are mostly affected during accidents? Is it children, hawkers or who tell me?

- Participant: The children and riders here being a junction at times you will see people moving from Npaha side reaching the junction instead of applying breaks he won’t and he has crashed, when you climb straight without looking left or right you can easily be crashed with a vehicle.

Interviewer: Which age category of children have you witnessed?

- Participant: Hmmm…. At time you see children from 11 to 15 years old the school going time it does happen.

Interviewer: What I am going to ask you is very sensitive, or personal but it will help us understand the situation here, if you feel comfortable can you share with me a story of an accident that you witnessed either someone or you yourself?

- Participant: A lot, one day I was just sitting here. As I said earlier on, one day I was just sitting here somebody rode a motor bike from this side coming here. Immediately he reach here he did not look and a car moving here paii…. On the spot and there were two we carried them to Tamale one passed on and one had a broken leg, so I saw many.

Interviewer: Have you witnessed a vehicle knocked down a child that you are willing to tell us?

- Participant: Yeah… a car knocked a student here that was the reason why we cause commotion here and they gave us this speed rumps. It about two to three years today, just knocked the child under spot.

Interviewer: Now let talk about the police and their role. How do you see their enforcement of laws regarding speed, helmets and license checking and broken vehicles on the road?

- Participant: Actually, hmmm if we are to talk about the police, we will talk a lot because me being a tricycle operator what they do they normally demand is the money that they take, checking your over loading no, checking your tires no, what they do is just to take their 10 cedis and go away actually for police deer they have problem.

Interviewer: So do you think all this affects crashes? What the police is doing?

- Participant: Yeah it affect accidents because your over loading can cause an accident and at times if you see the way tricycles are loading, because if you over load they are supposed to tell you that you have over load whether you reduce the load or you move but they will not tell you they will only take the money and the way they are supposed to patronizing on this road but that is not what we are seeing.

Interviewer: If you have the power, what would you do to change the situation here?

- Participant: If I have power what I will do is to make sure the police people check on every moving vehicle or tricycle and also the over load is also important if you see some of the tricyles they are old if you load them more and the tires too are not good if you load them more it can cause an accident and also the drivers those who normally pass the junction, when you get to the junction make sure that you limit your speed because anything can just happen somebody can just crossed so that you limit your speed and make sure everything is good.

Interviewer: When an accident occurs what happen? What do you think causes people to die or injured anytime an accident occurs, for example is it the condition of the vehicle that makes more likely for a severe injury or death?

- Participant: In fact if you come to this area most vehicles are not in good shape, the truth is even when you are entering into a vehicle you can easily wound yourself if you are not careful the vehicles are old and they are also loading over instead of three they are loading four four at time five that one alone is even dangerous than getting an accident so if you load four or five immediately the car involve in any trouble you will easily die or involve in any critical situation

Interviewer: Generally, if people typically get into an accident, which category of people are mostly affected? For example, is it pedestrians, children, motorcyclist or hawkers? Is it also those with helmets or without helmets or those who doesn’t use seat belts?

- Participant: It depends on the accident maybe it may knock another car when it crashes with another car that accident will be different from when it is landing, you know when it crashed with another car does in front they normally get infected but when it lands on the other side too those who are sitting by the doors they normally get infected.

Interviewer: How about the road environment? For example, broken down vehicles on the road, potholes, lack of sidewalks and traffic volume?

- Participant: Yeah, actually we don’t have much potholes but what we normally encounter is drivers you know somebody vehicle may get problem they will go to the bush and bring a rock on the road they will repair and after finishing they will just go and leave it or at times when a tire blast you know the pieces they don’t remove them from the road a night like this when you are riding and you are not careful you can easily crash it. That one too causes accidents too.

Interviewer: What can be done looking at this situation what do you think can be done to reduce the number of severities and deaths here?

- Participant: Hmmm to reduce…we should make sure that when the law says take three take three and when the law says take four take four but taking above what is supposed to be taken is dangerous but because of the money they will force you to enter the car and even if you are in the car you will not feel comfortable not to talk of involving in an accident and dying.

Interviewer: You talked about some accident scene that you witnessed, so I want to find out from you any time there is an accident that occurs in this road what happens? Do you call the police? Do people come to help? Do you call ambulance? Tell me what happens?

- Participant: Yeah they call the police and they call the ambulance at times we call the police and the police will also call fire service or at times too we call fire service straight so that both of them will come including ambulance.

Interviewer: When you call an ambulance do they come and how long do they take to come?

- Participant: Aaah… at time they come faster because when you call them and tell them that accident just happen just now here within some forty five minutes depending on where the ambulance is. At times you call them and they will tell you that the ambulance is not within the district maybe it has gone for a certain work that one deer they will also call Yapei, you know one is stationed at Yapei it will come and that one also depend on the time that it will come calling Bupei you may not get and they will also call Yapei. If you call Bupei and there is an ambulance it does come early.

Interviewer: If you call the ambulance, do they just come because you have called them, or they are looking at the caliber of the one that call them or the vehicle that call them or they just come?

- Participant: Hmmm… they are just coming they don’t ask whether he is a native, he is rich, he is fair they just come. When you call them and tell them accident has happened, they just come.

Interviewer: So, if you had the power, what would you do to improve care after an accident has occurred? Will you be increasing the number of ambulances or training more staff to give first aid?

- Participant: Actually Ghana we have a problem like Buipe Being the District capital it is suppose to have even two ambulance at Buipe or more because one may go to a certain then there will be a stand by ambulance but as at now we don’t have them like that so when accident happen and you call and the one at Buipe is not there they have to call at another ambulance somewhere to come at times they do call at Kintampo, then ambulance will move from Kintampo to this place

Interviewer: So, we are talking in general, in your opinion and your own estimation are accidents a much problem in Ghana?

- Participant: There a problem because losing one life is not easy so at times too it depend on our carelessness you know Ghana we are careless for that one is a fact instead of doing this to protect the people what we need is the money we don’t care. This vehicle just standing here like this you see how many people you can count? there are many and they don’t mind whether the tires are good or not… they don’t mind whether the tires were good or not but we are suppose to be checking all this to reduce some of this things, so accidents is a big problem.

Interviewer: So, looking at all these, if government is doing something about road safety do government consider your views and opinions when they make decisions on road safety?

- Participant: Anyway anyway today is the first day I’m experiencing this and I’m very glad I’m very happy that it happen this way because you are not around this our area, as you are getting our views to get to know what is happening here. I’m very happy you are here but since I was born in this community I have never seen anybody come to have this interviews or conversations with the natives of this area.

Interviewer: What do you think government is doing currently to reduce accidents in Ghana?

- Participant: Okay okah they are doing good because the traffic in town and other places, speed rumps, I can see that nowadays speed rumps are many moving from Buipe to this area you can get not much, but here we have speed rump, Kabilpe we have speed rump, Buipe itself we have but at first hmmm.. cars are just moving straight and that one is very dangerous, so I think the government is doing good in order to reduce road accidents.

Interviewer: What do you think government decides to do speed ramps in some places and not all places? Do you think government considers cost or what?

- Participant: Hmmm.. is not that they are cheap because constructing a speed rump I think I can’t tell I don’t know how much is involve but I can tell is not easy because you will need a lot of money to it. Because you will buy some many materials to construct a speed rump. The government doing it I’m the the government is only doing it to protect the people in that particular area.

Interviewer: Where do you think government get their ideas on road safety? Is it that they look to other countries or at research, do you have any knowledge regarding where all these ideas on road safety?

- Participant: Yeah… research is one, they also travel to different countries to see what is going on there and have to implement them here in Ghana. You know as a main road like this, this thing this white lane they have given it but now it is fade off, instead of them to do it again it protect, when you are riding in the night this thing give you the signal when you are on you lane you know, when you are going above you lane you know. This one they fade off when you are going you are just going you don’t know where you are going.

Interviewer: In some countries they use enforcement cameras to monitor the speed of vehicles on the road, and people get fine immediately if they over speed or run a red light- do you think we can do such a thing in Ghana?

- Participant: We can do such a thing you know government may put effort to do this but we the native we are not correct that is why I am blaming the police, the police service they are suppose to do that because if you move speed limit or you are driving or riding and you are speeding the police have the right to tell you to limit your speed or they can even arrest you but Ghana they will not do that. Even if they arrest you koraa they will just take money and leave you.

Interviewer: So If you are going to rate Government, what mark will you give government on a scale of 1-10 with 10 being the best and 1 being the poorest? What mark will you give government?

- Participant: I will give them 7 because you know they are putting effort, but the effort is not enough. They are trying their best to do that, but you know as human activities is too dangerous most especially in Ghana is not easy to do things.

Interviewer: This is our last question, if you have your own power, what will you do to reduce accidents, injuries, and deaths on the roads in Ghana? What will you do for pedestrians, motorists and children?

- Participant: One, education, education is very important, two make sure that this lane I’m just talking of, make sure they if they are fading make sure they are always intact because to make sure people will know either this is where I should be or not that one too will reduce an accident but such items normally fade off. And the police too, please they should talk to the police if they work, they should not look at the money, they should do the work as prescribed to them. In fact, if I have power I will put check on them, if you move from here to Kabilpe, there is a police barrier here if you reach them they don’t mind what ever they are taking is the money they don’t consider whether you get accident on the way.

Interviewer: Is there anything else that you want to add to this our conversation regarding crashes, injuries, and deaths on the road that we haven’t talked about today?

- Participant: We are pleading to the government to do more although what he is doing is not enough but he is trying his best but he should make sure that do more for people to reduce the accidents. Those who are in charge should make sure that the vehicle that on the road are very accurate and functioning. You know some of the vehicles you will be in the vehicle, and it will be shaking so it is easy to get accident and most the drivers too you will see young boys they don’t license and they are driving on the road.

Interviewer: Thank you so much for talking to us, we have really appreciated your time and your effort. Thank you.
