# Supplementary material for: “We are pleading for the government to do more”: Road user perspectives on the magnitude, contributing factors, and potential solutions to road traffic injuries and deaths in Ghana
Source: PLoS One. 2024 May 24;19(5):e0300458. doi: 10.1371/journal.pone.0300458 (PMC11125548; doi:10.1371/journal.pone.0300458)
Supplement: S2 File — (ZIP) [file pone.0300458.s002.zip › Transcripts to share/Participant_117_vulnerable.docx]

**Participant Number: 117**

**Language: Twi**

**Type of hot spot: Urban**

**Sex: Male**

**Road user type: Tricycle passenger**

Interviewer: You said you are farmer and how do you get to work? Do you use this road often? For example walking, public transport (trotros), motorcycles, cars, taxis, trucks, riding a bike, tricycles (i.e., pragya)

- Participant: I go to work with aboboyaa (tricycle)

Interviewer: How would you describe this area to others as far accident is concern? Is this road busy?

- Participant: Yes, this road is busy with accident.

Interviewer: How big of a problem do you think accidents are here?

- Participant: O! Many accidents have been happing here but for animals (live stocks) uncountable. In the case of human beings, it may be that within a year car may knock down about two people. Accident case is disturbing us over here.

Interviewer: What do you think causes accidents here? Example Road conditions (such as potholes, lack of sidewalks), abandoned/broken down vehicles, over speeding, wrong overtaking, traffic.

- Participant: what causes accident here is that before we were in need of speed bump, because over here the road is smooth. Again, over here too we’ve been crossing the road to farm so it may sometimes cause accident here.

Interviewer: What about road conditions (such as potholes, lack of sidewalks), abandoned/broken down vehicles, over speeding, wrong overtaking, animal crossing does this contribute accident?

- Participant: Over speeding is the cause of accident here.

Interviewer: What do you think decreases the risk of an accident?

- Participant: If we get speed ramp here accident will reduce.

Interviewer: Are there some people who are more likely to get into an accident (for example: children, hawkers)?

- Participant: Hmm we those people around here when the accident occurs like that it worries us and we too don’t even know what to do so when you are going to farm we mostly take our children along to the farm. Because you don’t if you leave them behind something may happened to them.

Interviewer: So those who have been crossing here we have children and adult over here when the accident occurs does it affect children ?

- Participant: Children are mostly affected.

Interviewer: Which age of children?

- Participant: Children of about three years, four years and like one and half year’s kids can cross the road with their brothers holding their hands and then eventually all that you here are the sound of an accident

Interviewer: Have seen an accident before?

- Participant: I have seen a lot.

Interviewer: If you feel comfortable, can you share a story from an accident with me? Your own or someone else you know?

- Participant: A lot, I have seen a lot of accident.

Interviewer: Can you share with us

- Participant: Some time ago, we were here and they take motor king pass by this road and then eventually we heard ‘payang’ (sound of accident) three people kill by aboboyaa (goods carrying tricycle). A while back, we were in this area and witnessed a motor tricycle pass through this road. Later, we heard a loud sound of an accident, and unfortunately, three people lost their lives due to a collision with an aboboyaa (goods-carrying tricycle). (Rephrased)

Interviewer: Can you tell me of a story about a child getting in an accident on the roads, if you have one?

- Participant: Emm, just this year, this child by us and it’s not up to a month over here. This boy’s younger brother a car run over him. He died on the sport. (Referring to a boy standing by us)

Interviewer: Sorry for your lost. Now, let’s talk now about the police and their role. What do you think about the police’s enforcement of laws now? For example, speed, motorcycle helmets, unlicensed driving, broken vehicles. Do you think this affects crashes?

- Participant: Ok! If they do police barrier over here accidents will reduce.

Interviewer: What do you think about the police’s enforcement of laws now? For example, speed, motorcycle helmets, unlicensed driving, broken vehicles. Do you think this affects crashes?

- Participant: Just us you said we those who ride moto should wear our helmet. Over speeding cars, the police should be checking them and to enforce the law to reduce their speed. So, when they do that accident will reduce.

Interviewer: If you had the power, what would you do to change the situation here?

- Participant: There are two things involve. If we get police to be station here. Or if force our self to construct a speed bump here. Even the speed bump will be more helpful to us.

Interviewer: When people get into an accident, or get hurt, what happens? For example, do people call the police? Do people come help? Does an ambulance come? Tell me about what happens.

- Participant: Yes, we call the police and they come. But when aboboyaa knocks a pedestrian when you call the police they don’t come. The one who knock the children, will himself send the children to the hospital and will same time report himself to the police. We those who live around we come to help.

Interviewer: When you call an ambulance, do they come?

- Participant: Ambulance only come when there is a car crash but if aboboyaa (goods carrying tricycle) knocks a pedestrian they don’t come.

Interviewer: How long would an ambulance take to arrive?

- Participant: About thirty minutes.

Interviewer: Who gets an ambulance and who doesn’t?For example, does it depend on if you are in an urban or rural area? Or the conditions of the road? Or if it’s a major road and it causes congestion?

- Participant: No, they don’t ask.

Interviewer: If you had the power, what would you do to improve care after an accident?For example, I will make ambulance to help us to construct speed rump here to reduce accident.

- Participant: if have the power I will say that every parent to keep their children in house and not to allow them out near the street.

Interviewer: In your opinion, how much of a problem are accidents in Ghana?

- Participant: Accident is a very big problem here. People are dying because of accident.

Interviewer: Does the government consider your views when they make decisions on road safety?

- Participant: When we talk to the government he does not listen.

Interviewer: What is the government currently doing to reduce accidents? For example, speed bumps, enforcement by police, pedestrian bridges, education campaigns Have you heard of those?

- Participant: Yes, I’ve heard of it.

Interviewer: Have you seen those?

- Participant: Yes, I’ ve seen it before.

Interviewer: Why do you think the government chooses these? For example, speed bumps, enforcement by police, pedestrian bridges, education campaigns. Are they considered better?

- Participant: The government consider that to be better.

Interviewer: Are they cheaper? Do you think the government considers cost when they pick what to do?

- Participant: Yes, he does consider cost sometimes because some of the projects are expensive some too are not.

Interviewer: Where do ideas about road safety come from?Do you think the government looks to other countries? Or at research?

- Participant: it is from the research that we are doing.

Interviewer: We know other countries use enforcement cameras, where people get a fine immediately if they speed or run a red light – do you think we can do such a thing in Ghana?

- Participant: Yes.

Interviewer: Why?

- Participant: This is will help but for me what I want is if they construct the speed rump, it will help because it will get to a time these people might not be there but the cars will always be passing.

Interviewer: The cameras are fixed there even if there is no one there it will capture the car for law to deal with the driver. Do you think this will help?

- Participant: Yes, it will.

Interviewer: Why?

- Participant: Because it is able to capture the speed of cars so I think it will help.

Interviewer: What mark will you give the government on a scale of 1-10 with 10 being the best?

- Participant: Zero

Interviewer: Why that mark?

- Participant: This because over here, the government has done nothing here.

Interviewer: Finally, our last question for you is: If you had the power, what would you do to reduce accidents, injuries, and deaths on the roads nationally? What would you do for pedestrians?

- Participant: If I am the government what you said catches over speeding cars, I will put some here and town too so that when cars get there, they can’t speed up. I will also construct speed bump so that when cars get here or town, they will slow down their speed.

Interviewer: What about motorcyclists?

- Participant: I will make police men stand here to check helmet and to check unlicensed riders.

Interviewer: What about for children?

- Participant: I will charge all mothers to take care of their children well and also arrest mother who allows her minor child out to cross the road.

Interviewer: Is there anything else about crashes, injuries, or deaths on the roads that we haven’t discussed today that you would like to tell me?

- Participant: What I will like to add is that if the government can come out and help us build speedbump here. I will be happy.

Interviewer: Thank you for your time and participation in this important work.
