# Supplementary material for: “We are pleading for the government to do more”: Road user perspectives on the magnitude, contributing factors, and potential solutions to road traffic injuries and deaths in Ghana
Source: PLoS One. 2024 May 24;19(5):e0300458. doi: 10.1371/journal.pone.0300458 (PMC11125548; doi:10.1371/journal.pone.0300458)
Supplement: S2 File — (ZIP) [file pone.0300458.s002.zip › Transcripts to share/Participant_118_non_vulnerable.docx]

**Participant Number: 118**

**Language: Dagbani**

**Type of hot spot: Rural**

**Sex: Male**

**Road user type: Driver**

Interviewer: My brother what work do you do?

- Participant: I am a driver,

Interviewer: Do you used a vehicle in this road?

Participant: Yes, I use this road and I even I go as far as Accra, Kumasi Techiman and even I go to Npaha in addition

Interviewer: Looking at this road is it busy for drivers and passengers?

- Participant: Yeah, this road is a main road and all the time is busy, every time this road is busy.

Interviewer: Looking at accidents and injuries in this road, are they a problem here?

- Participant: To be frank this road is a problem and accidents are rampant and I think majority of the problem are from we the drivers, we the drivers how we speed here is too much, and because of the way we speed if anything should cross your way sudden you don’t have anything to do than to fall down, have you seen….and sometimes overtaking, as a driver you don’t over take in some places but if you overtake then an accident will happen, have you seen.. and sometimes too sleeping, sleeping can let you fall with your car…because if you are on the road and you feel sleepy and you don’t stop but forcing going that one can cause accident. And some corners you haven’t seen what is in front of you, but you are overtaking before you realized someone is coming then you crash each other yeah that is what is causing the accident.

Interviewer: So if you at this place what will reduce the number of accidents here in this road, what will make the risks of accidents reduce?

- Participant: You are asking what will reduce risk of accidents?

Interviewer: Yes,

- Participant: As I was saying over speeding, if a bus is going small small and anything crosses you on the way you know what to do and land safely without being involved in an accident….but if you are going very fast even if a goat crosses you then you will fall down, all this ones causes accidents. And some people you know some drivers take drugs, if they take those drugs and feels dizzy all these can cause accident on this road.

Interviewer: So if you look at this road which people are mostly affected by accidents, children or hawkers or which people?

- Participant: to be frank this road and accidents it happens to a lot of people, the elderly, children accidents are just accidents it affect a lot of people here….haven’t you reach Buipe? One of our buses that got involved in an accident is lying on the roadside. The night journeys, this bus was moving in the night and wanted to overtake another car and bus appear in their front and they got crashed, my car type nuu because you did reach Buipe you would have seen it.

Interviewer: What I am going to ask you is very sensitive, I want you to tell me have you ever witnessed an accident here or someone else witnessed and talked about it that you would like to tell us?

- Participant: Hmmm just myself, I fell with car, this place Kadereso me myself I was coming from Kumasi, Kadereso corner just at the corner my front tire burst, and the bus fell see…the bus fell with us see the healed wounds it is up to some 15 years now, so it me myself that was using the bus. So this road that care about child or elderly, children can got involved in an accident and the elderly too can get involved. That is it

Interviewer: Have you ever witnessed a child being knocked down by a vehicle on this road, have ever witnessed or they talked about it, can you tell us about?

- Participant: Yes just this town a child was knocked down by a moving bus here that incident brought this speed ramp here, just here and also Kabilpe Dagomba line that one was even an elderly, it was over speeding that killed the child, the bus was coming and the elder man was also crossing and the bus hit the man and the man died, that is Kabilpe do you know there?

Interviewer: The police and their work regarding this road how do you see it?

- Participant: Ok the police they are doing their work correct but the way they are doing the money is their target, their target is just the money but they are working correct because if you come to their barrier you know they will check your papers but if they check whatever it is they will collect money from you, that how they are but they are working correct. You know if you check a vehicle papers and everything is correct you don’t have to collect money from the driver? But they will still collect that is what is happening, and we are fighting with them, is not anything

Interviewer: So the police not doing their work does it affect the number of accidents, what they were suppose to do and they are not doing?

- Participant: you mean the police?

Interviewer: Yes

- Participant: Yes, no as for the police they don’t have any problem, they don’t have any problem in this road, aahh… their work is just the barrier they don’t have problem, is the MTTU people that we have problem with they always stop us, when you come and your papers are not good then they tell you pay this and pay that before you can go and you pay and go, but we and the police we don’t have problem, as for barrier we don’t have problem.

Interviewer: If you have power, what will you do regarding this road and accidents will reduce?

- Participant: Hmmm ok If I have the power, you know the accidents mostly occurs in the night here so if I have the power no bus will move in the night, you know the accidents happens here mostly in the night some people sleep easily and before you realized he has dozen off and move to different lane and hit an oncoming vehicle, so if I have the power by 8pm or around 7pm no vehicle should move unless tomorrow this would have reduce most of the accidents here.

Interviewer: If you look anytime a car falls, we don’t wish for that may God forbid, assuming your car get accident what are the things that make people get more injured, is it the seats, condition of the car or what, what causes more injuries when accident happens?

- Participant: if you look at a vehicle everything is metal, even the seats covered with cushion are all metals, if a vehicle is about to fall it is the metal that is going to injured people, recently this place, Npaha Vehicle, the vehicle is at this petrol station, recently it fell, and when it was falling you know the metals that is the one always killing passengers

Interviewer: How about the seat belts, and wearing of it, does it protect people from injuries and deaths?

- Participant: Yes, you know we those with old old vehicles they don’t have seat belts, if it the new vehicles, if the cars falls down the seat belts will protect you when you get an accident and maybe how you were suppose to get injured may reduce because of the seat belts, you won’t be injured like that, but our buses here that have seat belts even the driver that have a seat belt neither to talk of passenger. Have you seen all this no seat belts so if it is going to do accident the passengers will all be scattered, the metals inside the vehicle will injure you only those with luck will be save

Interviewer: Looking anytime a vehicle crashed and people get injured which people are mostly affected, is it the pedestrians, or children or motorists or people ridi.ng bicycle or hawkers that are mostly affected?

- Participant: inside the vehicle?

Interviewer: When an accident occurs which people are mostly affected, children, hawkers, or which people?

- Participant: Those people walking on the road side are not in the car, it is those people inside the vehicle that are going to get injured, like how Yapei those people selling at the road side, those people at this road side if a vehicle should come and fall down they don’t have any problem it is the people inside the vehicle that are in problem so that is it, if I have my own way no commercial vehicle will use the road in the night, it is the cause of all the accidents and not anything.

Interviewer: How about the way the road is made or constructed does it bring about accidents?

- Participant: No the way is made that is the correct way,

Interviewer: How about potholes?

- Participant: It is the potholes that is causing many troubles, sometimes you will be dodging the potholes and that causes accidents here, but if the road is fine, everyone is on his lane, like how this place is made if a vehicle approach here it will leave its lane and be dodging the potholes to balance and go and that one can cause accident.

Interviewer: So if you have power what will you do regarding this situation?

- Participant: If I have the power what I will do here is to come and work on the speed bumps correct and it will be correct, have you seen the Yapei bridge, one aspect is the bridge is corroded leaving the other way so if any vehicle comes everyone is avoiding the bad road and entering the good place and that one has caused a lot of accidents on top of the bridge. They have done the bridge and one part has spoiled so when the vehicles comes, everyone wants to pass the good place, no one wants to pass the other way that place a lot of accidents happens on the Yapei bridge they have done the speed rump and one side corroded if you are coming from this side they leave their road and balancing the other side have you seen that causes a lot of accidents.

Interviewer: So anytime there is an accident here, do you call the police, or ambulance, what do you do?

- Participant: Anytime an accident occurs then immediately the police comes, whether it is a small car, big one or anything, even if you don’t call them before you realized they will be there at the spot. If the number of people involve is more, we will call Buipe ambulance or Tamale and they will come and pick them and go to the hospital but if it is not severe it is our own vehicles we will use and carry them to the hospital

Interviewer: So if the ambulance is coming, are they just coming because you call them or they factor the one who call them or the victims involved or they are just coming because they called them?

- Participant: because they call them, any human being who got involved in an accident, even if you are not big person and they call them, a vehivle has fell and people are injured if they call they will come and pick them. Hmmm they will carry them to Buipe, Buipe but if it is major injuries they will carry them to Tamale. That’s how we work in this road.

Interviewer: If you have the power what will you do regarding accident and ambulance, will you be increasing the number of ambulances or the first aid they give immediately after accident, which one will you be tackling?

- Participant: Yeah, I think the number of ambulances is supposed to be enough in case of accident, because sometimes we call them and they are not coming as expected, people will be lying down in pain have you seen so, the Buipe ambulance is only one and that is a problem, so if I have the power I would {inaudible}… increased the number of ambulances here and it is a big problem

Interviewer: Looking at Ghana, are accidents are they a big problem?

- Participant: Ohai! Is a big problem, is it too much in this road, it has been occurring all the time in this road that is why I told you if I have the power by 7pm all vehicles whether you are in Yapei or Buipe or Burkina Faso and you have just reach Buipe that is where you will sleep when day break you will continue your journey, have you seen, if I have my power that is what I will let them do because the accidents are caused by the night travels and that is the problem

Interviewer: The way government is doing their work road safety, do they come and listen to you before they do their work or they just do how they feel like doing?

- Participant: They don’t come like that, government they don’t come like that no, they just doing their work they don’t come like that not at all, they just work because they own everything so who would they consult again, they just do whatever they want

Interviewer: So, looking at Ghana, what is the government doing to reduce road accidents, what have they done that you have seen and you can see this is what they are doing to reduce road accidents?

- Participant: Yes, truly they said they are doing speed ramps to reduce accidents and the speed ramps they are doing it in town, so the speed if you go out of town you just increase your speed again, so the way see government is not doing anything to reduce the accidents, what I think government can do is what I told you by 7pm they shouldn’t allow any car to move. All these accidents will not have been seen again.

Interviewer: So the speed rump government is doing in town and leaving some place including outside town, why are they doing that, is it lack of money or those ones they are doing is what they can afford?

- Participant: they doing it in the town is because of children, children and pedestrians to cross the road that is why they are doing it in town, but if you move outside the town no one is there to cross unless cows that will cross you, no one is outskirt town.

Interviewer: Looking at government doing speed rumps, things to reduce road accidents, do they look at the money involved when they are doing these things or they don’t consider money when doing these things, do they consider money, or they don’t?

- Participant: Okay they look at money when they are doing, you know if it is not money they are looking at while they are working, they can’t do the work.

Interviewer: Looking at government doing these road safety measures, where do they get their ideas from, do they look at other countries or they do their research?

- Participant: {inaudible}…here in Ghana, is it Ghana own money they are using, or you don’t know, so the accidents here is not small, there are a lot of accidents.

Interviewer: So some countries have speed cameras, if you over speed the cameras will know and they will catch the driver, if they bring such a tool to Ghana will it work?

- Participant: Yes that thing will work, that thing is in Saudia Arabia, ok here everyone with his vehicle, you know Saudi Arabia it is company company, if you take your company trailer and go speed they are monitoring you and you cant over run have you seen so because of that they are no accidents there. Even if you have your own vehicle, you can’t go beyond at is recommended speed but if in Ghana here government can he do this thing? Will we agree? I don’t think we will agree I told you the speed is too much, I told you it is the speed that causes the accidents.

Interviewer: If they say you should give government mark from 1 to 10 which number, will you give, the meaning is government hasn’t done anything and the 10 is perfect work done by government, what mark will you give?

- Participant: The government has done something well looking at this road sometimes the problem is coming from we the drivers because is where the problem is coming from.

Interviewer: So what mark will you give, one is he has done well if you give him 10 he has done perfectly, what mark will you give?

- Participant: I will give him 10

Interviewer: You can give numbers in between 2,3,4,6 up to 10.

- Participant: Government has done well, because what they are doing for us regarding this road, they have done well.

Interviewer: We are just finishing like that, if you have the power and you are the one in charge, what will you do regarding people crossing the road, so that accidents will reduce,for motor riders, and children crossing roads?

- Participant: Yes, we know that accidents are the will of God but if I have the power, just as I told you if I was a government or have the power, the night travel by vehicles is what causes a lot of accidents, if I was government or have power then we will stop all vehicles from night travels

Interviewer: Apart from the night travels, pedestrians, and children crossings what will you do for them?

- Participant: All those ones are better, like children crossing road like a town like this the speed ramps are there because of the children crossing and the elderly crossings that’s why do them, the government has done well in terms of the town protecting people, if you go to Npaha area there is no village you won’t see speed rump, you will see a lot of speed rumps up to 4, 5, and 6 but it use not to be there, they came and worked on the road and put those speed ramps over there because of school children, and because of the elderly, If I have power….inaudible}

Interviewer: How about the motor riders?

- Participant: Is because of all those people that is why they did the speed rumps, if you get to the speed rump you reduce but if you go outside the town then you go your going.

Interviewer: What do you have to add to this conversation, anything to add?

- Participant: What I have to add is you people should talk to government so that the government will work regarding our vehicles, if you go to Ouagadougou I have forgotten the name of the town by 7pm no vehicle moves again, every vehicle will be parked unless daybreak then everyone will continue their journey, so that is what I have for government, by 7pm every vehicle big vehicles, small vehicles by 7pm wherever you are you should remain there daybreak then everyone will continue their journeys, the accidents will have reduce.

Interviewer: Thank you so much, we really appreciate.
