# Supplementary material for: “We are pleading for the government to do more”: Road user perspectives on the magnitude, contributing factors, and potential solutions to road traffic injuries and deaths in Ghana
Source: PLoS One. 2024 May 24;19(5):e0300458. doi: 10.1371/journal.pone.0300458 (PMC11125548; doi:10.1371/journal.pone.0300458)
Supplement: S2 File — (ZIP) [file pone.0300458.s002.zip › Transcripts to share/Participant_119_non_vulnerable.docx]

**Participant Number: 119**

**Language: Twi**

**Type of hot spot: Urban**

**Sex: Male**

**Road user type: Driver**

Interviewer: How do you get to work? For example walking, public transport (trotros), motorcycles, cars, taxis, trucks, riding a bike, tricycles (i.e., pragya)

- Participant: I am a driver who works at Offinso Cement factory and I drive from Ofinso to Tamale.

Interviewer: How would you describe this area to others as far as accidents are concern? Is this road busy?

- Participant: Over here accidents often happen. Sometimes a car might be behind and may blast the tire before you realized that car will be somersaulting. Some, too overtaken then eventually collide with another car.

Interviewer: How big of a problem do you think accidents are here?

- Participant: Human lives are lost.

Interviewer: What do you think causes accidents here? Road conditions (such as potholes, lack of sidewalks), abandoned/broken down vehicles, over speeding, wrong overtaking, traffic.

- Participant: some of the accidents are due to potholes. Also, with speed bumps, the road sign that will alert you to know that there is a speed bump ahead may be right there where the speed bump is. So, while on top speed before you realized you are right at the spot of the speed bump, and when it veers you it can cause an accident. Sometimes too overtaken can be one of the causes of the accident here. Some drivers ~~too, more~~ are careless, they overtake anyhow. The car that I am using, I mean this daf trailer parked over there, some drivers can overtake you whiles maybe you’ve ~~neared~~ drawn closer to him but will still overtake you.

Interviewer: What about fatigue, ~~maybe~~ you may be before the steering wheel but will be tired

- Participant: Sometimes too, accident is caused by fatigue and sleeping while driving. Some of us may be sleeping and instead of him stopping and resting, he’ll force his way through. In such case when sleeping elude you it’s either you pass through the bush or ~~to~~ crash head on with a car without your consent.

Interviewer: What do you think decreases the risk of an accident?

- Participant; what can reduce accident here is that anytime, ~~we~~ the drivers have to be careful, overspeeding and wrong overtaking and at sometimes you are tired but still you will be driving. If you are tired you have stop and rest. I believe if we are able to do all these accidents will reduce a bit for us.

Interviewer: Are there some people who are more likely to get into an accident (for example: children, hawkers)?

- Participant; Over here the farmers especially, some of the accidents too affect motor riders who have been crossing us and also the passengers involved in a crash.

Interviewer: Please are children involved in the accident?

- Participant: children are among the affected people.

Interviewer: Can you tell me of a story about a child getting in an accident on the roads, if you have one?

- Participant: Yes, sometimes ago but not all that long. Where you were standing before there was a sprinter by the roadside. We happen to meet at the scene immediately after the accident. There were children and adults all seriously injured.

Interviewer: Which age of children?

- Participant: Some were very small including a toddler, some were breastfeeding babies, age-wise they were about one year, two years, and above three years and more. Some were in their crowing ages.

Interviewer: Sometimes personal stories can make road traffic problems more real. However, we know this can be sensitive. If you feel comfortable, can you share a story from an accident with me? Your own or someone else you know?

- Participant; As for us, since we have been roaming, sometimes we will only see the car laying by the roadside with its passengers wailing and shouting. Or sometimes too you may even pass by and it happens after you. In such case you can’t do anything to help the passengers except to call the police.

Interviewer: Now, let’s talk now about the police and their role. What do you think about the police’s enforcement of laws now? For example, overspeeding, motorcycle helmets, unlicensed driving, broken vehicles. Do you think this affects crashes?

- Participant; The police do their best to enforce the law but after that the rest lies on the drivers. As am saying if there is a barrier here, the police will check everything for you to pass. What about after you have pass? He’s behind you so the rest lies on you the driver who have to use your mind.

Interviewer: For example, over speeding, motorcycle helmets, unlicensed driving, broken vehicles. Do you think this affects crashes?

- Participant: the above is the major contributor to road accident here. Also, broken-down vehicles contribute to some road accident. somebody may be overtaking and it may be that a car has broken-down ahead of him on the road without a warning sign, before you realized. an accident occurs [So, what do you think the police should do about it] So, what I want the police to do every time a vehicle breaks down on the road the police should tow that vehicle from the road or make sure there is a warning triangle to warn any approaching vehicle on the impending danger. That one will help us.

Interviewer: If you had the power, what would you do to change the situation here?

- Participant: if I get that power, I will see to it that the police by the road side anytime either the patrol or MTTU team to be on their toes. Still, we have machine they use to check over speed that one too can help a bit (speed gun). It is up the police to see to it that any time they will work with it. Or a passing car this is its speed limit. Also, a broken-down vehicle they will see to that it is towed. Or the potholes too I will see to it that the urban road has fill them so as to help us reduce accident.

Interviewer: Once an accident does happen, What do you think causes people to die or get hurt, compared to just getting into a crash without getting hurt? For example, what about the condition of the vehicle or trotro makes it more likely for a severe injury or death? Like seat belts not working in cars/trotros, cars being old and not having air bags, position of seats, crowding

- Participant: In car crashes, speeding is part, even if the car is brand-new and the speed is great in a crash life can be lost. At sometimes too, some cars are very week so much so that a little crash you see that the car will damage seriously or part broken into pieces or scramble and in turn causes severe injury to the passengers. That one too is part.

Interviewer: Generally, which people typically to get injured or die in an accident? For example, pedestrians, children, motorcyclists, bicyclists, hawkers those without a helmet, those who do not use seat belts

- Participant; Over here what I haven’t seen. Any time there’s an accident it’s the passengers onboard who are mostly affected.

Interviewer: What about the environment (such as the roads) makes it more likely for severe injury or death? For example, abandoned/broken down vehicles on the road, lack of sidewalks, potholes, traffic volume on roads

- Participant; Well, as for the road that am using is very good except that there’s small pothole and the pedestrians’ walkways are there too. So, it’s the potholes that disturbs us.

Interviewer: What can be done to reduce the number of severe injuries and deaths here?

- Participant; If we check the cars on the road it will help. Some cars don’t deserve to be on the road but yet they are. [why are you saying that] because the car is extremely weak. At times they don’t have good tires. Just as you said, maybe the driver don’t have license too so whiles going if anything that happens on the road, he careless in his mind. Also, the seatbelt too the drivers have to wear any time they are driving so that if something happens, they can control the car. So, if we make this a necessity, it will reduce accident on our road.

Interviewer: When people get into an accident, or get hurt, what happens? For example, do people call the police? Do people come help? Does an ambulance come? Tell me about what happens.

- Participant: Yes, people call the police.

Interviewer: When you call them do they come?

- Participant: Yes, they come.

Interviewer: When you call an ambulance, do they come?

- Participant: Yes, they come.

Interviewer: How long would an ambulance take to arrive?

- Participant; O! it does not take long. When you call they respond within seconds.

Interviewer: Who gets an ambulance and who doesn’t? For example, does it depend on if you are in an urban or rural area? Or the conditions of the road? Or if it’s a major road and it causes congestion?

- Participant; They don’t do that, they care for all.

Interviewer: If you had the power, what would you do to improve care after an accident? For example, increasing number of ambulances, training people around in first aid.

- Participant: At sometimes the ambulance is small

Interviewer: So, if you have the power what will you do?

- Participant: so if get the power I will have to increase the number of ambulances here. And after the accident, how to remove the injured passengers from the car I will see to it perfection. For some if you don’t handle them with care, it may aggravate their injury. So, for that aspect its emergency situation so we have to be careful when dealing with such casualties, for some of the accident you have cut the wreckages to save the passengers. So, in such case if you happen to meet that accident and you don’t handle with care you even end up the life of that passenger.

Interviewer: In your opinion, how much of a problem are accidents in Ghana?

- Participant: Accident issues in the country are horrible and problematic to the entire nation. To me a lot of human life is lost. Sometimes when you see the wreckages of the car you cannot even cut any part to save the injured passenger. So over here when accident happens it is fatal here.

Interviewer: Does the government consider your views when they make decisions on road safety?

- Participant: Yes, he listens.

Interviewer: What is the government currently doing to reduce accidents? For example, speed bumps, enforcement by police, pedestrian bridges, education campaigns

- Participant: To my knowledge, the government constructs speed bumps to control the speed of drivers and ultimately decrease the occurrence of accidents.

Interviewer: Have you heard of those?

- Participant: Yes.

Interviewer: Have you seen those?

- Participant: Yes.

Interviewer: Why do you think the government chooses these? For example, speed bumps, enforcement by police, pedestrian bridges, education campaigns

Interviewer: Are they considered better?

- Participant: Yes, the government considers that to be better.

Interviewer: Are they cheaper? Do you think the government considers cost when they pick what to do?

- Participant: he sometimes considers the cost.

Interviewer: Where do ideas about road safety come from? Do you think the government looks to other countries? Or at research?

- Participant; Based on the research that you are doing;

Interviewer: We know other countries use enforcement cameras, where people get a fine immediately if they speed or run a red light – do you think we can do such a thing in Ghana?

- Participant: Yes, we can and it will help us a lot. I wish it will be implemented very soon

Interviewer: Why?

- Participant The reason I said it will help is that, when you first asked me, I said over-speeding is the cause of most accidents in the country. Because in over speeding if you blast your tire there is no way for you control the car. So, if the speed camera comes it will help us.

Interviewer: What mark will you give the government on a scale of 1-10 with 10 being the best?

- Participant; Five

Interviewer: Why that mark?

- Participant: They have a lot to be done to improve accident issues in the country.

Interviewer: Finally, our last question for you is, if you had the power, what would you do to reduce accidents, injuries, and deaths on the roads nationally? What would you do for pedestrians?

- Participant; For pedestrians I have to do something so that it will show the pedestrian that here you have to cross and here you don’t have to cross.

Interviewer: What about motorcyclists?

- Participant: I will see to it that always they have to wear their helmet before they ride.

Interviewer: What about for children?

- Participant: I will see to it that any place children cross I will do zebra crossing at that place.

Interviewer: Is there anything else about crashes, injuries, or deaths on the roads that we haven’t discussed today that you would like to tell me?

- Participant: What I have notice about the road that is dangerous is at times the speed bump we construct to protect us. For some there is no speed bump that is what I see is disturbing us a bit. If it is there, they build it very close to the speed bump. Instead, they should have built it with a little distance away from it so that it will alert the drivers before they get there. But it is not done like that so, they happen to get to it on the spot. Also, they should construct the speed bump with reflected tripes so that it will alert the drivers from a distance. This will help reduce accident.

Interviewer: Thank you for your time and participation in this important work.
