# Supplementary material for: “We are pleading for the government to do more”: Road user perspectives on the magnitude, contributing factors, and potential solutions to road traffic injuries and deaths in Ghana
Source: PLoS One. 2024 May 24;19(5):e0300458. doi: 10.1371/journal.pone.0300458 (PMC11125548; doi:10.1371/journal.pone.0300458)
Supplement: S2 File — (ZIP) [file pone.0300458.s002.zip › Transcripts to share/Participant_120_non_vulnerable.docx]

**Participant Number: 120**

**Language: English**

**Type of hot spot: Rural**

**Sex: Male**

**Road user type: Driver**

Interviewer: Hi, my Boss can you tell me what time of work you do, I mean how do you move around?

- Participant: I am a driver, I use this road all the time, I use this road from here to Tamale, Bolga and even to Kumasi and Accra. Sometimes I do go from here to Kintampo.

Interviewer: How do you describe this road? Is it a busy road?

- Participant: This road is a very busy road, it is a busy road sometimes accidents occurs here all the time.

Interviewer: Okay, how big is the problem with accidents here?

- Participant: Accidents are a big problem the bus drivers, the Yutong bus drivers some of them when they are feeling sleeping, they won’t pack they are forcing to reach where they are going so that causes a lot of accidents here

Interviewer: So, what do you think will decrease the risk of accidents here?

- Participant: What me I think will decrease the risk of accidents here the Yutong buses at least they should give them two two drivers it will help them. One driver when he is tired it can cause an accident. One may feel sleeping so when they are two drivers if one feeling sleeping then one will also take errh.

Interviewer: Are there some people who are more likely to get into an accident for example children, hawkers or who?

- Participant: This our village deer, at least we have two speed rumps so when car passing here they are not speeding so accident is not happening in the town but going to Tamale side about two kilometres far away from this place accidents do causing a lot. Even last year four buses, here the same thing here to Tamale and from here to Buipe accidents do causing a lot on this road.

Interviewer: How about children? Do you have any occasion where children were involved here?

- Participant: No no accidents involving children on the village no, once a while when it happens two to three four years before it happened again

Interviewer: Do you have a story where a child was involved in an accident here?

- Participant: Yes, I have about two or three like this car packing like this it was break time around 10 0cklock the student came to break so they were crossing, so one car pack like this a small girl just come to cross so the car from Tamale going to Buipe doesn’t know that a child wants to cross and it knock her down. About five years old girl.

Interviewer: Okay great! do you have any personal story of someone an adult who got involved in an accident here that you want to share with us?

- Participant: Yes, yes, last two years ago one motorbike like this it carried two people from Npaha coming. It was speeding, one trailer too was going to Accra. So it came here it didn’t stop then knocked the trailer and they fell inside so the trailer passed on the two people and they died. One was about forty-three years and one is twenty ~~three~~ years.

Interviewer: I see, so let talk about the police and their role in terms of law enforcement, what do you think there are? Are they enforcing the laws?

- Participant: The police actually I didn’t trust the police. The police are not doing their work. Even last year one trailer and then articulator car knocked here. So the tractor was on the road. Me myself personally I used my phone to call the police to come and see how we manage so that the tractor will come out small so that the road be there when car passing they refuse to come. So a kia rhino came carrying water melon and knocked it that the same day and fell down.

Interviewer: Do you think the police not doing their work affects the number of accidents here?

- Participant: Yes, the police is not doing their work and that one is causing accidents because they packed at one place they are not patrolling and when accidents too happen seriously, and you call them they won’t come early. That one they used to be doing it all the time here and we are seeing it.

Interviewer: Ok thanks, if you have the power and you are the one at forefront of issues here, what would you do to change the situation here?

- Participant: If I have power deer I will do my best because all the time I am on the road so on the road if I have power I will do a lot to help people so that accidents will reduce.

Interviewer: So what do you think causes people to get hurt or die if there is an accident? Example, is the condition of the vehicle like seat belts working or not working, overcrowding or cars being old?

- Participant: You know mostly this northern region deer… people don’t check the seatbelts so when they sit in the car inside they wont use seat belts so when an accident happens we use to die more. Last year, about thirty something lives lost at this place just last year only. It was first 207 bus and about 14 people and then Yutong buses came and crashed about 19 people. Another Yutong buses the same week and crashed about 19 people. Sarkikoraa from here to Npaha junction about 2 kilometers from this place we called that place Kanbonakoraa. Even this time they set police barrier over there because of that accidents.

Interviewer: Thanks, ok generally which people typically get injured or die in an accident? For example are pedestrians, children, or motorists likely to get died in an accidents or those with/without helmets?

- Participant: No, it is the people travelling in the car, travelling from Kumasi, last year these buses last year one bus load from Zebilla accident here the same bus from Zebilla. That 207 bus also load from Kumasi to Tamale even this year self, one sprinter car is lying down at this place, they didn’t take the sprinter self it kills people a lot. Those travelling from get accidents most.

Interviewer: So let talk about the road conditions, how about things like potholes makes it more likely for a severe injury or death?

- Participant: I believe some of the drivers they are sleeping the road deer… is fine because no potholes from here to Tamale from here to Kintampo no potholes. The drivers they used to sleep and that is why I said if I have power I would have let them use two two drivers. Most of them if one is tired, he is sleeping no one to collect him.

Interviewer: So what can we do to reduce the number of severe injuries and deaths here?

- Participant: I believed if the authority people can stand and look at all this things and advise the drivers when you are tired you can pack it will reduce the accidents and the crossing too how they are crossing by heart and car knocking them we advise our women those selling on the streets when they are coming they should leave their children in the house and come because they are not matured. So children too they don’t know how to cross they wont watch they will be crossing by heart so we advise them not to be bringing their children on the streets.

Interviewer: So tell me when accident happens do they call the police, do they call ambulance, do people come to help? Tell me what happens after an accident occurs?

- Participant: When accident happens we call police, we do call the police if we go there early then we will be helping then we call the police to come if they come they will also call ambulance or fire service. Some of the car it will cause accident the passengers you cant take them out of the car unless fire service people come and help so we do call them.

Interviewer: How about the ambulance? Do you call them, and do they come? And how long do they take to come?

- Participant: Sometimes they come fast and sometimes they delay eer…sometimes you call and they say the ambulance has already travel sometimes too they come very early.

Interviewer: If you call the ambulance, do they just come because you have called them, or they are looking at the caliber of the one that call them or the vehicle that call them or they just come?

- Participant: Hmmm that one deer… I don’t know their big mens but all the time when accident happens when we call them and they come who’s with severe injuries they pick them first, those they have minor minor ones they will leave them and go and come back and pick them too.

Interviewer: And this rural area, do ambulance come or only in the cities?

- Participant: Yes they come. We have one ambulance at Buipe and we have one at Yapei so when at times we call they do come together to pick the peoples who injured.

Interviewer: So you said that sometimes when you call the ambulance they delay before coming or they are at somewhere. So, if you had the power, what would you do to improve care after an accident has occurred? Will you be increasing the number of ambulances or training more staff to give first aid?

- Participant: Assuming at least you know this is northern region most people they fear God when accident happens like this we do stop some cars even we understand the passengers they will alight so that they will take those they get injured to the hospital so that they will also help because cars will do accident and even one ambulance can not take them so will we want to increase the number of ambulance so that it will take everything once some they will take the injury people and go some will die.

Interviewer: So, we are talking in general, in your opinion and your own estimation are accidents a much problem in Ghana?

- Participant: Oh Ghana deer… accidents is a problem because even our village here what I’m witnessing not Kumasi Accra road, so accidents is having problem in Ghana too much.

Interviewer: So, looking at all these, if government is doing something about road safety do government consider your views and opinions when they make decisions on road safety?

- Participant: They just do… apart from today what I see you doing I didn’t see any road safety came here to advise something or no noo…

Interviewer: What do you think government is doing currently to reduce accidents in Ghana?

- Participant: That one deer.. I don’t know but most of the villages they are doing speed rumps because this place they have done about two even they brought eerrh… chippings self they said they want to module one but have not started yet but this one is helping us small small accidents has reduce at this village

Interviewer: What do you think government decides to do speed ramps in some places and not all places? Do you think government considers cost or what?

- Participant: Oh they just do the speed rumps any how some of the speed rumps it doesn’t last when the feeder roads people come they wont do the cement enough inside so that when car passes on it again then it got broken.

Interviewer: Where do you think government get their ideas on road safety? Is it that they look to other countries or at research?

- Participant: Ntoa! That one deer… I don’t know but at first this place accidents was causing too much, we decided to make our own speed rump so it was very high they brought soldiers here so we all run away and leave the village before they were able to come and make this small ones so how they make I don’t know how it cost.

Interviewer: In some countries they use enforcement cameras to monitor the speed of vehicles on the road, and people get fine immediately if they over speed or run a red light- do you think we can do such a thing in Ghana?

- Participant: You know Ghana when you bring MTTU or MTTD they will pack at one place, they will be collecting money they wont do the work if car is speeding self and they catch the driver they will just collect money and leave him. They will not let the law deal with him, if only they get their money finish. So accidents here before accidents will reduce in Ghana unless our top top official talk to the police people those their MTTD or MTTU because MTTU they are not doing the work they are stopping motorkings taking in money when it is speeding they will take money they wont deal with him so this thing you know Ghana.

Interviewer: So If you are going to rate Government, what mark will you give government on a scale of 1-10 with 10 being the best and 1 being the poorest? What mark will you give government?

- Participant: I will just give them out of 10 I will give them three (3), I will give them 3% because they are not doing well.

Interviewer: If you have your own power, what will you do to reduce accidents, injuries, and deaths on the roads in Ghana? What will you do for pedestrians, motorists and children?

- Participant: Where will I get this power hehehe…. Can I get this power, assuming they give me the power I will let the police the MTTD patrol, they should patrol or if a driver driving careless or speeding they should deal with him or take him to the law or this place we don’t obey the law, drivers some of them they don’t have license and they are driving but when police catch them and say pay 300 or 400 cedis and they pay then they are going. Here accidents do some cars will get accident and kill people before the driver will go and pay money to the licenses office and say they should back date the license so that he will use to cover himself it happens all the time at this place. Most of the drivers are not having license. When they go too they will do the license for them and back date the license for them. I see one or two three four, I know.

Interviewer: Is there anything else that you want to add to this our conversation regarding crashes, injuries, and deaths on the road that we haven’t talked about today?

- Participant: What I want to say or what I will advise like if government look into this police because they are road safety people at least the MTTD my advise to them is some of the drivers they don’t have license and they are driving, and some of them too they are drunk they drink before they drive, here somebody will be driving careless and passenger stop then they will start fighting so here our law doesn’t work so I will advise my senior officers if they will try to solve all this things it will have help us.

Interviewer: Thank you for your time and participation in this important work, I thank you so much your patience.
