# Supplementary material for: “We are pleading for the government to do more”: Road user perspectives on the magnitude, contributing factors, and potential solutions to road traffic injuries and deaths in Ghana
Source: PLoS One. 2024 May 24;19(5):e0300458. doi: 10.1371/journal.pone.0300458 (PMC11125548; doi:10.1371/journal.pone.0300458)
Supplement: S2 File — (ZIP) [file pone.0300458.s002.zip › Transcripts to share/Participant_122_vulnerable.docx]

**Participant Number: 122**

**Language: Dagbani**

**Type of hot spot: Rural**

**Sex: Male**

**Road user type: Motorcyclist**

Interviewer: You said you are always on this road, right?

- Participant: Yes, I use the road to Damango to Busunu, and Yapei to Buipe

Interviewer: So, looking at this road is it busy?

- Participant: Yes, all the time is busy

Interviewer: So are accidents a problem here?

- Participant: Yes, accidents always happen here due to undue overtaking

Interviewer: {Inaudible}…And what again?

- Participant: like potholes sometimes someone can dodge a pothole and go and fell down or he would go and collide on someone and then cause trouble.

Interviewer: So, what can decrease the risk of an accident here?

- Participant: What will reduce accident is the police being around {inaudible}… if the police around that one will also help and the accidents decrease

Interviewer: So, if you look which people are mostly affected by accidents here, which people is it children, women or who?

- Participant: Children around 10 years, and around 5 years trying to cross the road has been bringing accidents, the person will want to cross, and the vehicle will with speed and knocked down the person.

Interviewer: Do you have any story of an accident here that you can share with us ….{inaudible}?

- Participant: A knocked down some here in town, the person was crossing the road and the car knocked down and killed the person.

Interviewer: How old was the person?

- Participant: Around 10 years old child.

Interviewer: Do you have another incident of an accident here?

- Participant: inaudible…yes, one vehicle coming and when he got to the corner and fell and one person died.

Interviewer: So, if you look at the police work how do you assessed their work and accidents?

- Participant: Their work regarding accidents…aahhh over speeding and it is occasionally that they do like Mondays, and they don’t patrol around they are sitting at their place and be stopping people.

Interviewer: So, because of what they are not doing does that affect the number of accidents?

- Participant: They are not all concern about helmets, and someone can go and fall down and because the person is not wearing accident oh! I mean helmets the person can die.

Interviewer: So, if you have the power, what will you do to reduce the number of accidents here?

- Participant: if I have the power I will have increased the police presence here so that they be around here and be patrolling in order to reduce the speed here, or some of the motors riders sometimes they don’t have helmet or he will be riding or instead of him to know that there are police in this road so I should reduce my speed if they catch me they can do something to me. so all this if I have power I will make sure they are around.

Interviewer: So anytime there is an accident what makes people get more injured, is how the passengers are made to sit in the vehicle, what makes people more injured?

- Participant: What is in this place is the dying in the vehicles cause overloading in the buses that is our problem.

Interviewer: How about seat belts presences in the vehicles and number of loadings?

- Participant: Yes, if it seat is for you they will double two people on it, all this the vehicle loading over can cause accident.

Interviewer: So, if you look at accidents which people are more likely to be involved in an accident, is it children, farmers, inaudible}?

- Participant: sometimes some people can be going to their farms and accidents will reach them, it has been happening, motor riders, bicycle riders it has been happening in this road?

Interviewer: How about the way the road is made, does it bring about accidents I mean potholes?

- Participant: Pothole like on this road sometimes someone can be coming, and you are in front and you don’t know about the person and he will come and dodge a pothole and collide on you all this one has been causing accidents.

Interviewer: So, what will you do if you have the power to reduce the number of accidents here?

- Participant: if I have the power, like the potholes it is supposed to be filled and that one will reduce the number of accidents.

Interviewer: So anytime there is an accident do you call the police, do you call ambulance, what do you do?

- Participant: if a car gets accident we call ambulance people and sometime ago it was not here but now we have it so when you call them they will come if they don’t come it could be petrol like shortage of petrol especially like this our village.

Interviewer: So, if you call them and they are coming do they look at the caliber of the person involved or they are just coming?

- Participant: We will just call them, and they will come If you call them, we will call them and they will come.

Interviewer: So, if you have the power what will you do regarding the situation, the fuel shortages you mention, with regards to the ambulances having fuel shortages if you have the power what will you have done?

- Participant: If I have the power with regards to this information, I would help the situation.

Interviewer: Will you be increasing the number of ambulances and looking at the fuel situation?

- Participant: I would have increased the number of ambulance and make sure they have fuel all the time.

Interviewer: So, looking at Ghana are accidents a big problem for us?

- Participant: Big,

Interviewer: Why do you say so?

- Participant: The child is a future somebody and he close from school and car will come and knocked mean while he is future person don’t you see all these things send us back

Interviewer: So, if government is doing their work on road do they come to listen to your concerns and do road works?

- Participant: government government can be doing let just take village for example {inaudible} we want ambulance oh! Sorry speed rumps like three or four but we only have one all this if there was any help and you have the power we will have done four or three.

Interviewer: So, you see speed rumps here in this place?

- Participant: yes, it is only one it is not up to was it was supposed to be.

Interviewer: So, in your opinion, is it that government doesn’t have money that is why they did only one speed rump or what do you think why you were given one speed rump?

- Participant: In my thinking the government doesn’t see us in this our village to be anything, they don’t respect our lives.

Interviewer: So the speed rump and the other things in your opinion where do government go and get those ideas from, is it that they went to some countries or they do their own research in your opinion what do you think they get the ideas from and they speed rumps {inaudible}?

- Participant: Those ambulances oh! Speed rumps

Interviewer: Yes?

- Participant: To the best of my knowledge anytime people lay complains to the district assembly, it was the assembly we complain to and they came and did the speed rump for us.

Interviewer: In some they have speed cameras, and it monitors how drivers are going with vehicles, if you go more than what is expected it will capture you and you will be arrested, if they bring such cameras to Ghana will it work?

- Participant: like speed rumps,

Interviewer: Speed cameras that they will bring and if drivers and it will monitor how drivers are moving with their vehicles on the road, if you over speed they will know and catch you if they bring it to Ghana will it work?

- Participant: it will work, for example just see the vehicle that came and passed here, does it look like we are in town? It doesn’t look like so if the speed camera should catch someone and the law deals with the person, and someone sees that will he do the same thing?

Interviewer: If they say you should mark government from 1 to 10, and 1 is he hasn’t done anything and 2 maybe has done something small up to 10 and 10 is what you wanted that is how he has performed what number will you give?

- Participant:{ Laugh} is it this government?

Interviewer: No all successive governments and road safety personnel what number will you give?

- Participant: What we are happy with is this current government said if they win, they will give us free education, so we were thinking it would better, so that is what we believe.

Interviewer: So, what number will you give, 1, 2, 3 0r 10?

- Participant: oh the number that I will give

Interviewer: Yes?

- Participant: I will give 1

Interviewer: Why do you say 1?

- Participant: I will give 2

Interviewer: Why do you say 2?

- Participant: because after they won government, Savanna region, they have given us Savanna region

Interviewer: If you have your own power, what will you do for people crossing the roads, and children going to school and farmers using the roads to reduce accidents on the roads?

- Participant: If I have the power, one I will do enough speed rumps here so that it will reduce the accidents and If I have the power the police patrolling so if I have the power, I will let them be serious going around and be checking over speeding.

Interviewer: So, do you have anything to add to this our conversations, do you have anything to add in terms of accidents, motor riders or drivers do you have something to add?

- Participant: I have said it all because the over speeding is the thing that is causing the accidents, if you don’t over speed you won’t be involved in an accident.

Interviewer: We thank you so much.
