# Supplementary material for: “We are pleading for the government to do more”: Road user perspectives on the magnitude, contributing factors, and potential solutions to road traffic injuries and deaths in Ghana
Source: PLoS One. 2024 May 24;19(5):e0300458. doi: 10.1371/journal.pone.0300458 (PMC11125548; doi:10.1371/journal.pone.0300458)
Supplement: S2 File — (ZIP) [file pone.0300458.s002.zip › Transcripts to share/Participant_123_non_vulnerable.rtf]

Participant Number: 123
Language: Twi 
Type of hot spot: Rural
Sex: Male 
Road user type: Driver
Interviewer: How do you get to work? For example walking, public transport (trotros), motorcycles, cars, taxis, trucks, riding a bike, tricycles (i.e., pragya)
·	Participant: I use car
Interviewer: How would you describe this area to others? Is this road busy? 
·	Participant: Sometimes Very busy Sometimes too not so busy, one thing I can say is that the journey is very far. It may depend on where you are coming from. For me I drive from Tema to Tamale, so maybe while driving I may feel tired, quickly I have to park the car and rest. So that nothing bad will happen to me. The journey is very long for me.
Interviewer: How big of a problem do you think accidents are here? 
·	Participant: Very big problem to us.
 
Interviewer: What do you think causes accidents here?  Road conditions (such as potholes, lack of sidewalks), abandoned/broken down vehicles, over speeding, wrong overtaking, traffic 
·	Participant: As for me the whole thing I can say is that may be over speed.
Interviewer: Again, what do you think causes accidents here? Road conditions (such as potholes, lack of sidewalks), abandoned/broken down vehicles, over speeding, wrong overtaking, traffic. 
·	Participant: Over speeding, abandoned/broken down vehicles, wrong overtaking and motor riders without helmet all among the reasons for accident occur here.
Interviewer: What do you think decreases the risk of an accident?
·	Participant: what I can say is that unless you make the road double road.
Interviewer: Are there some people who are more likely to get into an accident (for example: children, hawkers)? 
·	Participant: The passengers in the car.
Interviewer: Sometimes personal stories can make road traffic problems more real. However, we know this can be sensitive.  If you feel comfortable, can you share a story from an accident with me? Your own or someone else you know? 
·	Participant: I have heard and seen some before but myself I have never experience some before. What I can say is that even with these goods, on my way coming when I reached Tekyiman I saw some motor guy involve in an accident which has cause a traffic jam. So, I stop the car until the cars started moving. From there where they said that someone on a motor bike fell off from his moto bike and a car behind run over him. His head got broken.
Interviewer: Can you tell me of a story about a child getting in an accident on the roads, if you have one? 
·	Participant: No, I haven't seen some before.

Interviewer: Now, let's talk now about the police and their role. What do you think about the police's enforcement of laws now?  For example, speed, motorcycle helmets, unlicensed driving, broken vehicles  Do you think this affects crashes?
·	Participant: What matters most on the side of the police is that when you see that the car has a problem just park the car, there is no need for them to take money from the driver and allow him to go. I remember sometimes a go; I was an apprentice in track driving. We use to load from Accra to Takwa and there was a tanker driver who was feeling sleepy whiles driving instead of him to park the car and rest, he did not and was forcing his way out. So, my master forces our car to overtake that car. Our car had a problem but yet my master force to overtake that car and then reported that car to the next police barrier. Whiles on the way we spotted the car coming from behind. So, on this note I can say that sometimes the police activities too contribute to accident on the road. Also, those who ride without helmet the police has to ensure that they wear their helmet. If they are two, both of them should wear their helmet. Therefore, with what I saw on my way coming if he was wearing his helmet his head would not have broken. 
Interviewer: What about those without license
·	As for cars, before you move a car you should have your license with you. The company had no right to take your license and say go and come back for your license. If you drive without license the police can stop and ask you where is your license and you reply company has taken my license, he will collect money from for nothing and live you to go. Some of the drivers has lost their license for about three months when police ask and find they have, they only pay money and go away.
Interviewer: what about broken down vehicles
·	Participant: Again, on broken down vehicle; I will use my case as example my car had a problem while in tamale. I call the office and they said I should manage until I reach Kumasi but on my way the car broke down in the middle of the road. I stop another track to use its tale to push me to the proper side of the road. If I had known this track will leave me in the middle of the road, I would have left it in Tamale. It has never happened to me before. So therefore, the activities of the police can contribute to road accident.
Interviewer: If you had the power, what would you do to change the situation here? 
·	Participant: The best thing I will do is get one towing in this place. So, any time a car broke down within thirty minutes that car has to be remove from the road. So that the road will be free. You where my car has broken down, one might think it is in a good position but it's not. It looks save only because of the speed bump which surround it but in actual Sense it not in the right position. So therefore, if we had a tawing car here, they would been tow it. The police should think about the life of the people and stop taken those small-small coins and others. 
Interviewer: Once an accident does happen, what do you think causes people to die or get hurt, compared to just getting into a crash without getting hurt? For example, what about the condition of the vehicle or trotro makes it more likely for a severe injury or death? Like seat belts not working in cars/trotros, cars being old and not having air bags, position of seats, crowding 
·	Participant: Our road too contributes to accident. The car too you see, if the car is old and the front tires is not all that good, very old. So, when you drive through a road which is not all that good and you hit a pothole all that you see is that the front tire got burst. From there you can't control the steer again and the car will rather control you whether to the bush, ditch or whichever place. And if there is speed in the car at the time when the tire burst no matter what people will die. When the speed is down and the front tire is good if you hit a pothole for the steer to get off your hands it will be difficult for people to be injured or die and lack of  maintenance that causes people die when there's accident.
Interviewer: What if the car is old.
·	Participant: Humm that is what I said the maintenance, when the car is old and lacks maintenance, he may say let me go and come back to maintain it. You may go and come back and go again but the third time or next you might no know what will happen next.
Interviewer: What about car without airbag?
·	Participant: That one too is part because when there is airbag and there happens to be a crash the balloon will pop up towards your face to save you and your head from crashing the windscreen.
Interviewer: What about the car with their seat close to each other's?
·	Participant: All is among the cause of accident on the road. You see the driver who sit before the steering wheel without seat belt. Even with the seat belt if something happens it can hold you fast to the seat. If the seat belt is functional and the shake you could see that the seat holds you fast attach to the seat so that you can't move forward too much.
Interviewer: Generally, which people typically to get injured or die in an accident?   For example, pedestrians, children, motorcyclists, bicyclists, hawkers, those without a helmet, those who do not use seat belts
·	Participant: If the accident happens on the high way the driver suffers more but when it happens in the town is where the accident sometimes affects those by the road side or those who sell by the road side.
Interviewer: What about the environment (such as the roads) makes it more likely for a severe injury or death? For example, abandoned/broken down vehicles on the road, lack of sidewalks, potholes, traffic volume on roads
·	Participant: Over here what I can say is that if a broke down on the road without triangle that is the cause of most accident here. Even if there is a triangle and it does not reflect as it supposed to reflect, a car with speed can crash that broken car on the road. For the road is good except that if it were to be double road accident would have reduced.
Interviewer: What can be done to reduce the number of severe injuries and deaths here?
·	Participant: Just as I said, the position of my car is good but not all that good if we had a towing care here within thirty to an hour, he would have straitened me well. Whatever he takes then he takes and go his way. 
Interviewer: When people get into an accident, or get hurt, what happens? For example, do people call the police? Do people come help? Does an ambulance come? Tell me about what happens. 
·	Participant: Yes, the people around the villages that called the police. 
Interviewer: When you call an ambulance, do they come? 
·	Participant: Yes, they come but it last longer before they come.
Interviewer: How long would an ambulance take to arrive?
·	Participant: They take a long time to come. The last time, my master said he happens to meet a crash when they call an ambulance it was about an hour and there was no ambulance there to carry the casualties. So, he carries them in his container cargo car to a distance before ambulance chase him for them. 
Interviewer: Who gets an ambulance and who doesn't? For example, does it depend on if you are in an urban or rural area?  Or the conditions of the road? Or if it's a major road and it causes congestion?
·	Participant: O! they don't do that. Just that the time within which they take to arrive is the problem.   
Interviewer: If you had the power, what would you do to improve care after an accident? For example, increasing number of ambulances, training people around in first aid.
·	Participant: I will increase the number of ambulances. we have announced that any child less than 10 years should not be allowed to walk by the street alone or cross the road. I will also admonish anyone who drives and meets an accident to try and help convey the person to the hospital or the mortuary if he can to save lives.
Interviewer: In your opinion, how much of a problem are accidents in Ghana?
·	Participant: Accident issue in the country is unbearable. Every year the number keeps on increasing.
Interviewer: 
·	Participant: They listen but they don't work with it.
Interviewer: What is the government currently doing to reduce accidents? For example, speed bumps, enforcement by police, pedestrian bridges, education campaigns. Have you heard of those? 
·	Participant: NO
Interviewer: Have you seen those? 
·	Participant: NO
Interviewer: Why do you think the government chooses these? Are they considered better? 
·	Participant: Yes they are consider to be better.
Interviewer: Are they cheaper? Do you think the government considers cost when they pick what to do?
·	Participant: No, he does not but he does what is good for the community. 
Interviewer: Where do ideas about road safety come from? Do you think the government looks to other countries? Or at research?
·	Participant: From research and also, he learns from other country.
Interviewer: We know other countries use enforcement cameras, where people get a fine immediately if they speed or run a red light – do you think we can do such a thing in Ghana? 
·	Participant: Yes

Interviewer: Why? 
·	Participant: It will really help a lot. If some is installed here it will check over speeding. 
Interviewer: What mark will you give the government on a scale of 1-10 with 10 being the best? 
·	Participant: one 
Interviewer: Why that mark? 
·	Participant: Because they have not performed well to me. Also, some interventions they've done already like the speed bumps, are questionable. They don't last and sometimes when they don't attend to it can even cause accidents and damage vehicles.
Interviewer: Finally, our last question for you is: If you had the power, what would you do to reduce accidents, injuries, and deaths on the roads nationally? What would you do for pedestrians?
·	Participant: The whole thing is speed limit camera should install on all the road to check over speeding cars. So that we will know the speed limit to use when we get to town and speed limit to use while on high way.
Interviewer: What about motorcyclists? 
·	Participant: if some ride a motor bike without a helmet when you are caught you should fine that is the best thing.
Interviewer: What about for children?

·	Participant: We have to make an announcement that no child below the age of ten to fifteen years should be allowed to play around the road or cross the road. If a car knocks that child, his parent should be arrested. That one is very important but the road too there is no zebra crossing here except speed bump and so if we get zebra crossing about two or three here it will be better.  since there is no zebra crossing here where ever anyone want to cross they just cross.
Interviewer: Is there anything else about crashes, injuries, or deaths on the roads that we haven't discussed today that you would like to tell me? 
·	Participant: Accident is always there so we should careful on the road so as to avoid accident.
Interviewer: Thank you for your time and participation in this important work.
 
