# Supplementary material for: “We are pleading for the government to do more”: Road user perspectives on the magnitude, contributing factors, and potential solutions to road traffic injuries and deaths in Ghana
Source: PLoS One. 2024 May 24;19(5):e0300458. doi: 10.1371/journal.pone.0300458 (PMC11125548; doi:10.1371/journal.pone.0300458)
Supplement: S2 File — (ZIP) [file pone.0300458.s002.zip › Transcripts to share/Participant_124_vulnerable.docx]

**Participant Number: 124**

**Language: English**

**Type of hot spot: Rural**

**Sex: Male**

**Road user type: Motorcyclist**

Interviewer: How often do you use this road?

- Participant: I use it all the time.

Interviewer: For what purpose?

- Participant: To carry loads

Interviewer: How do you usually get around? How do you get to work? Do you use this road often? For example, walking, public transport (trotros), motorcycles, cars, taxis, trucks, riding a bike, tricycles (i.e., pragya)

- Participant: I use moto king, tricycle moto.

Interviewer: How would you describe this area to others? Is this road busy?

- Participant: Actually, is not all that serious but it is scary because at times the way the cars normally come from Accra side to Tamale side. At times when they to the community they don’t actually normally slow down if not because of this one speed rump but the time this one was not there always accident was just happening even killing with animals and then those things.

Interviewer: Is this road busy?

- Participant: Yes its always busy.

Interviewer: How big of a problem do you think accidents are here? How dangerous is it?

- Participant. Actually, is not all that dangerous but with the speed of the cars it’s scared to cross.

Interviewer: What do you think causes accidents here? Road conditions (such as potholes, lack of sidewalks), abandoned/broken down vehicles, over speeding, wrong overtaking, traffic Whats your opinion?

- Participant: It’s over speeding that normally cause the accident here.

Interviewer: What do you think decreases the risk of an accident?

- Participant: Elm by given a sign to the drivers to notice that when they getting to the community, they should slow down their speed to may be some forty limit or may be fifty limits.

Interviewer: Are there some people who are more likely to get into an accident or more likely to get injured? (for example: children, hawkers)?

- Participant: Yeah, it’s likely accident to be occur because students is crossing the road all the time and there are at times some too whose sight are not all that clear them. They normally cross the road to the mosque so when they are crossing it’s a little bit difficult for them.

Interviewer: Does accident occur to children or do vehicle knocks children when they are crossing the road.

- Participant: yeah, some time ago it does knocks them but because of this ramp that it has reduces those accident.

Interviewer: Which age of children? Participant: From eight, seven, nine there about.

Interviewer: Sometimes personal stories can make road traffic problems more real. However, we know this can be sensitive. If you feel comfortable, can you share a story from an accident with me? Your own or someone else you know?

- Participant: mmm the accident that has been occur here because at times cars and moto bike normally crash on this ramp. At times some one may come with over speed and hit the ramp and then fall.

Interviewer: I want you to tell me a story about an accident you have heard or you have seen. Tell me how it happened.

- Participant: An accident that occurred that I have heard is just like yesterday’s own a woman was coming from the farm with a bicycle then a moto rider full stick lotto’s (is a brand of lotto company) and was going to Tamale with his lotto’s and then all of the sudden hit the woman on the way and the woman died.

Interviewer: O! sorry

Interviewer: Can you tell me of a story about a child getting in an accident on the roads, if you have one?

- Participant: For that one it does happen some time ago because even across the road here a car last hit a child like that even the child is there, she has even grown up but at times too we’ve seen some sign of like he’s having a mental problem.

Interviewer: how old was that child who was hit by the car?

- Participant: At that time, he may be around nine to ten years.

Interviewer: Now, let’s talk now about the police and their role. What do you think the police do that reduces road accidents?

Interviewer: What do you think about the police’s enforcement of laws now? For example, speed, motorcycle helmets, unlicensed driving, broken vehicles Do you think this affects crashes?

- Participant: For the police I think maybe they should because this area there is no any police around may be if the police check point was here may be that one too will also reduce the accident that has been happening because here there is no any police check point around and due to that the road is free for them to speed up, or to speed but if they see that there are police ahead they will also reduce their speed because they don’t want also to involve with the case of police that they are over speeding.

Interviewer: What about people riding motorbike without helmet? what do you think the police should about it.

- Participant: Mmm that one too the police should arrest those without helmet and let them know that the helmet is very-very important for them to use. And also let them to know that their life is at stake if they are going without the helmet.

Interviewer: What about drivers driving without license

- Participant: for them I think the police should just arrest those that are not driving without license because for that one you are not qualify to be a driver because nothing shows that you are a driver in Ghana.

Interviewer: What about broken down vehicle on the middle of the road without a warning sign.

- Participant: actually, to me the police should always patrol on the road to also say that may be any car that spoil on the road they should have a tawing car that maybe it seems that this car the way its on the road it is very dangerous it can cause accident those one too helps to remove those one from the road.

Interviewer: If you had the power, what would you do to change the situation here as far as road accidents are concerned?

- Participant: Well, if it was up to me, I would suggest that those cars that are over-speeding, they should have something that will be checking on the speed whether this car that is coming from far distance is using this kind of speed that is coming so those ones if something is there to checking those ones that one too will help to reduce the accident.

Interviewer: Once an accident does happen, What do you think causes people to die or get hurt, compared to just getting into a crash without getting hurt? For example, what about the condition of the cars used here? Are they good or bad? Or motorcycle? Or the condition of the motorcycle? Like seat belts not working in cars/trotros, cars being old and not having air bags, position of seats, crowding

- Participant: Actually, some are good some are not good because some may have some like you will see the car self the car doesn’t deserve to be on the road. But because of Ghanian situation black man situation (??) he’s moving the car around with anyone concern.

Interviewer: For example, cars without seatbelts? Or cars with old age or the cars which have been very very old? To extend a little crash just become a scratch product? Do you think all these things contribute to an accident?

Interviewer: what about old cars does it contribute to road accident.

- Participant: Yes, because those cars that has been too old because the metal that was use to make the car is also weak especially this cargo cars so you see that the way the car, the structure of the car doesn’t deserve to be on the road and mostly some those passenger cars there are most of them don’t have seat belt and that one any little shake from the car it can also cause or may be a flat tire maybe the car may not even fall but people may get injured in the car.

Interviewer: What about the seats that in the car which are overcrowded?

- Participant: that one normally causes accident most if the car had an accident like that passenger that are in because of overcrowded in the car, they will just crash with the other seat and many will be injured.

Interviewer: Generally, which people typically to get injured or die in an accident? Example, children, walking around selling, motorcyclists that do not use helmets?For example, pedestrians, children, motorcyclists, bicyclists, hawkers peoplethose without a helmet, those who do not use seat belts

- Participant: it’s the moto rider that normally involve in the accident.

Interviewer: Lets talk about the road. Is the road good? To a standard? Is the road condition good? Are there any potholes? Does the road contribute to the accident in this area?

Interviewer: What about the environment (such as the roads) makes it more likely for a severe injury or death? For example, abandoned/broken down vehicles on the road, lack of sidewalks, potholes, traffic volume on roads

- Participant: No, the road condition is good but the driver’s carelessness that normally causes overtaking an those things Let’s say for an example, a broken vehicle like this, which is spoiled in the middle of the road without a warning triangle, abandoned vehicle, then they will leave it without a warning sign, potholes, a road without a sidewalk where by you and me can walk comfortably by the roadside. Here motorcycles are taking the pedestrians road.

Interviewer: So what do you think? If you had the opportunity what are you going to do to avoid all these things?

- Participant: so to me, I would have think that those motos and then the cars should be on one way because its not like a city that they will just be following each other with a gap. So for me, I would suggest that the motos leave the pedestrian way and be on the main road.

Interviewer: So you, if you have the authority, what can you do to reduce the number of severe injuries and deaths and accidents in this area? If you have the power?

- Participant To me I would have pass a law that because most accident that normally happens at times it does happens may be someone may be walking around and maybe someone may alking on the pedestrian way and the moto bike will also be forcing to pass may be thinking that the person should also come in the gutter and give him way to pass. So, to me I would have suggest that those with the machines to be on the road and then those with bicycles and pedestrians to be on the side of the road.

Interviewer: When people get into an accident, and get hurt, what happens? For example, do people call the police? Or do the police hear it themselves and come? Do the people call the ambulance? Or does the ambulance hear it themselves, hear it, and come?

- Participant: they normally call the ambulance to come.
- Participant: Yes, people from our neighborhood come to help.

Interviewer: When you call them do they come?

- Participant: Yeah, they do come.

Interviewer: Is it the police or the ambulance?

- Participant: the ambulance do come and the police too they do come

Interviewer: Can you give me a timeframe by which the police and ambulance do come?

- Participant: when accident happen like that maybe it will take about some let me just say some ten minutes at times maybe some fifteen minutes for them to come.

Interviewer: Ok now on the ambulance, when the ambulance comes, do they kind of select the kind of people they have to help? Do they look at the background of the injured person? Is he from a rich family? Is he a millionaire? Or does he come from a royal family?

- Participant: No, they don’t look at any person may be whom you are where you are coming from, they do help if its anyone at all.

Interviewer: Or do they look whether the fellow is coming from an urban area or from a city? Do they look at those things?

- Participant: No, they don’t check those things. They will just help that person to get to hospital.

Interviewer: If you had the power, what would you do to improve the care after an accident as far as ambulances are concerned?

Interviewer: So if you had the power, what would you do to improve care after an accident? As far as ambulances are concerned? For example, increasing the number of ambulances, training lay people in first aid to decrease the number of accidents. If you had the power what would you do?

- Participant: So, to me I will have first of all train the people around the community to have first aid for those people before the ambulance team will come and take over that one would have help to reduce the pain of the injured person.

Interviewer: If you had the power, would you reduce the number of ambulances in this area?

- Yes please, because those ambulances, there are not too many.

Interviewer: Now lets go to high level questions, for the entire country, in Ghana here.

Interviewer: In Ghana here, how much of a problem are accidents? Is it worrisome? Is it disturbing us? Is it scaring us?

- Participant: Actually, its scaring. Because of the accident when you are to traveling self your heart is beaten whether you will get to your destination or not because as you are going always you’ve been hearing accident happened here and there even some times on your way going you will see some. As you are also going whether it will happen to you on the way, you don’t know. So, it’s scarry.

Interviewer: Does the government consider your views or hear and listen to you when they make decisions?

- Participant: So, actually, when they make a decision like that, even government look something or maybe it will take much time.

Interviewer: Does the government take decisions when something is troubling you, like when you travel and is scary, and you make a complaint does the government listen?

- Participant: For that one, I would just say they don’t listen.

Interviewer: For example, when you make a complaint about a speed bump or a speed ramp, do they listen?

- Participant: They don’t listen. Because, Actually, the accident that has been happen police themselves normally come when and complaints have been given to them but nothing happens. They will just say it was all. And when it will pass that’s all.

Interviewer: When it comes to education? Do the government give you education? Concerning road education and how to manage things and road users? Do the government give you education?

- Participant: No please.

Interviewer: Okay, have you ever heard of government constructing road speed ramp, footbridge, and educating people on how to use the road? Have you ever heard it?

- Participant: Yes, I’ve seen heard it But for my area we do make complaint and complaint and complaint and this is the only one that has been made here.

Interviewer: Have you ever see yourself, speed humps, pedestrian or foot bridge, educating people on road accidents? How to use the road? How you should cross the road? Or the police enforcing laws on you? Have you ever seen it?

- Participant: Yeah, I have ever seen it. Because some times in places when you are to cross the road, you have to cross through the zebra crossing past that way across the road.

Interviewer: Why do you think the government chooses zebra crossings, speed ramps, why do you think the government chooses those interventions so that it will help to reduce accidents on the highways? Do you think they are cheap? Or do you think its better? That’s why the government chooses those interventions?

- Participant: Yes, its good its good but to me I would suggest that the speed ramp and the zebra crossing sometimes the driver doesn’t follow it but maybe if he hits it with his car the car will get affected

Interviewer: Do you think the foot bridge, the government educating people concerning road uses, educating us so that when we are crossing the road, we will know how to cross so that in our case we will not crash then. This education, and speed ramps, and zebra crossings, do you think the government considers them to be better, or to be cheap rather than picking other else things? Do you understand the question?

- Participant: the footbridge to me is good because that one, the cars will be under and you will also cross on top. So to me, the footbridge if is there will help than the other ones.

Interviewer: Okay, where do the ideas about safety come from, do you think the government go and travel outside and get that knowledge and bring it the country? Or do you think the research that we are doing that we submit to the government and he learn and apply it?

- Participant: I would think that people suggest to them and they will learn something from it.

Interviewer: Now, do you know, and have you heard of speed cameras? What are they used for?

- Participant: So it used for capturingthose cars with the speed. Because when the car is coming it normally take the speed that car is using and maybe those in the office too to get to know watching those cameras will get to know that this cars is over speed here so they have the say to them to reduce their speed when they are using the roads.

Interviewer: Okay now good, so do you think if these cameras were brought to this country, would they help reduce road accidents?

- Participant: This will help to reduce accident because no one want to be a victim. Because when they had you with speed like that maybe the will penalize you. Because you don’t want to also be maybe or use the money that you will also use for household purposes maybe to the police or something like that.

Interviewer: So, okay so on a scale of one to ten, I mean you want to mark the government’s performance [I understand] on road accidents and road safety to improve it. On a scale of one to ten with one being the lowest and ten being the highest, which mark would you give the government?

- Participant: One being the lowest, ten being the highest, Five in the middle. I would give give to him.

Interviewer: Why five?

- Participant: Because accident do happen all the time.

Interviewer: Maybe you did not understand my question well. I am saying mark the government with one being the lowest, one meaning he did not well. Two he is doing something. Three he is doing better. Five he is better. Ten he is done well and its true that accidents have reduced drastically in our country. So how much mark would you give to the government?

- Participant: I would give him five, because accidents do happen too. Even not in my area some certain places because of bad roads.

Interviewer: What has he done to reduce those accidents – okay our last question is if you had the power, what would you do to reduce accidents and deaths in our country nationwide especially people by the road side and cars knocking them again motorcycles without helmets and children crossing the road without care what would you do to reduce that?

- Participants: To me some places where the road is busy so to me, I will make sure there is a, what is the name, a foot bridge and there are certain places maybe either to make sure that the pedestrian way is different from it, may be on the same way but it should have a sign on the road directing the pedestrians that this is their way. Or to reduce pedestrian way so that car may not have their way. Or moto bike that is been mingle with them on the same way will reduce.

Interviewer: What about motorcyclists? Those who are using motos without a helmet?

- Participant That one, I would made sure that moto bike is seized. Because if your moto bike is being collected and struggle to get it or you spent some thing to get the moto bike you will make sure that for what you have spent will even buy more two helmets so you will buy the helmet in order not to fall that situation.

Interviewer: Thank you very much. Now we want your opinion, of all the questions we left behind when we bring it in it will help our next questions. Is there anything else we didn’t say about crashes that you think we should have said, bring it out.

- Participant: For me, I would say and I think that there should be some government that have some people that will always maybe remember people about the road accidents in the communities and the cities so that everyone will have an awareness of the accidents on the road maybe if you are crossing you need to do this or do that or look at your left and your right before crossing the road and have some people who are going around and doing those things maybe once in a month or three times in a year. So that people will always have mind on how to cross the road in order to reduce the accidents.

Interviewer: Okay thank you very much.
